# 16^th^ International Ecdysone Workshop: July 10–14, 2006, Ghent University, Belgium

**DOI:** 10.1673/031.007.1301

**Published:** 2007-03-08

**Authors:** Organized by:, Xavier Bellés, Isabelle Billas, Peter Cherbas, Jean-Paul Delbecque, Tarlochan Dhadialla, Haruchiko Fujiwara, Ronald Hill, Kiyoshi Hiruma, R Hermann, Kostas Iatrou, Jan Koolman, René Lafont, Jean-Antoine Lepesant, Yoshiaki Nakagawa, Reddy Palli, Alexander Raikhel, Lynn Riddiford, Huw Rees, Frantisek Sehnal, Karl Slama, Guy Smagghe, Kluas-Dieter Spindler, Colin Steel, Luc Swevers, Carl Thummel

**Affiliations:** ^1^ InStitute of Molecular Biology, Barcelona, Spain; ^2^ University of Strasbourg, France; ^3^ Indiana University, Bloomington, Indiana, USA; ^4^ University Bordeaux, France; ^5^ Dow AgroSciences, Indianapolis, Indiana, USA; ^6^ University of Tokyo, Kashiwa, Japan; ^7^ CSIRO, North Ryde, Australia; ^8^ Hirosaki University, Japan; ^9^ RheoGene, Philadelphia, USA; ^10^ NCSR Demokritos, Athens, Greece; ^11^ University of Marburg, Germany; ^12^ Université Paris VI, France; ^13^ Insitut Jacques Monod/CNRS, Paris, France; ^14^ Kyoto University, Japan; ^15^ University of Kentucky, USA; ^16^ University of California, Riverside, USA; ^17^ University of Washington, Seattle, USA; ^18^ University of Liverpool, UK; ^19^ University of South Bohemia, Czech Republic; ^20^ Academy of Sciences, Prague, Czech Republic; ^21^ Ghent University, Belgium; ^22^ University of Ulm, Germany; ^23^ York University, North York, Ontario, Canada; ^24^ NCSR Demokritos, Athens, Greece; ^25^ University of Utah, Salt Lake City, USA

Chitin synthesis and degradation are recurrent, fundamental events of insect development. The final step of chitin anabolism is the polymerization of UDP-*N*-acetylglucosamine units which requires chitin synthase activity. We present here results on the cloning and characterization of the chitin synthase 1 (CfChS1) gene from the spruce budworm (SBW; *Choristoneura fumiferana*), an important North American forest pest insect. Using degenerate primers from conserved regions of other insect ChS, a *CfChS1* fragment was isolated by PCR on a cDNA library made from freshly ecdysed, L6 SBW larvae. The full length of the *CfChS1* cDNA was determined to be 5.3 kb by the sequencing of overlapping cDNA clones and 5-RACE experiments. The encoded enzyme is 1565 amino acid long and possesses the two catalytic domain signature sequences found in all insect ChS (EDR, position 858 to 860 and QRRW, position 895 to 898). Computer-assisted analysis also predicts the existence of 16 transmembrane helices in the amino acid sequence, implying that CfChS1 N-and C- termini share the same (extracellular) topological space. The expression of *CfChS1* mRNA was closely associated with larval-larval and larval-pupal molts as well as with the formation of adult cuticle. In larvae, mRNA was generally absent during intermolt periods, but accumulated to high levels immediately after ecdysis, consistent with renewed chitin synthesis. Accumulation was also observed 24h before and after this event, and even up to 48h after ecdysis to the 6^th^ larval instar. *CfChS1* expression was restricted to the epidermis and did not accumulate in fat body or midgut tissues. Treatment of larvae with ecdysone and with the non-steroidal ecdysone agonist tebufenozide, repressed the transcription of *CfChS1*, within 6 to 12h of application. These results indicate that *CfChS1* expression could be stimulated by the falling 20-hydroxyecdysone titers that characterize the late phase of the molting process. We are currently investigating the interplay between *CfChS1* and other genes expressed during molting in the SBW.

