# Supplementary material for: 16th International Ecdysone Workshop: July 10–14, 2006, Ghent University, Belgium
Source: J Insect Sci. 2007 Mar 8;7:13. doi: 10.1673/031.007.1301 (PMC2999422; doi:10.1673/031.007.1301)
Supplement: Related texts

# Research article: Ftz-F1 homolog of *C. elegans* regulates cell fate decision by interacting with β-catenin signaling

**DOI:** 10.1673/031.007.1303

**Published:** 2007-03-08

**Authors:** M Asahina, T Valenta, M Silhankova, V Korinek, M Jindra

**Affiliations:** ^1^ Biology Center, Czech Academy of Sciences, Branišovská 1160/31, České Budějovice, Czech Republic.; ^2^ Department of Molecular Biology, University of South Bohemia, České Budějovice, Czech Republic.; ^3^ Institute of Molecular Genetics, CAS, Prague, Czech Republic.

The *Drosophila* nuclear receptor βFtz-F1 is well studied as a central regulator of metamorphosis in response to ecdysone signaling. However, the molecular mode of βFtz-F1 action remains only partly understood. Interestingly, recent studies have shown that mammalian Ftz-F1 homologs SF-1 and LRH-1 interact with Wnt/β-catenin signaling to modulate expression of specific target genes. Consequently the crosstalk between the nuclear receptor and Wnt/β-catenin signaling pathways determines important decisions such as cell differentiation, proliferation or malignant growth. We have examined interaction between a Ftz-F1 homolog NHR-25 and a β-catenin signaling pathway during a specific cell fate decision with the advantage of the *C. elegans* model. The Wnt nuclear effector TCF/POP-1, a NEMO-like kinase LIT-1 and two distinct *C. elegans* β-catenins, WRM-1 and SYS-1, are all required for asymmetric cell division of two somatic gonad precursors (SGPs), which produces a pair of distal tip cells (DTCs) that lead differentiation of the gonadal arms in hermaphrodites. Impaired function of any of the respective genes causes a Sys (Symmetrical sisters) phenotype, when all SGPs adopt the same, proximal fate. Thus, no DTCs and no gonadal arms are formed. Here, we show that loss of NHR-25 causes an extra DTC phenotype, where up to four DTCs form at the expense of the proximally fated cells. This “all-distal” Sys phenotype is thus opposite to known loss-of-function phenotypes of genes in the β-catenin pathway. Our data show that a balance between the β-catenin/POP-1 activity and the action of NHR-25 is required for the proper establishment of both the distal and proximal cell fates. *pop*-*1*(*q645*) mutants that never form DTCs and consequently lack gonadal arms develop DTCs, extend the gonadal arms, and become fertile when *nhr*-*25* is silenced in them. By using cell transfection techniques, co-immunoprecipitation, yeast two-hybrid and GST pulldown assays, we show that this interaction is coordinated by mutual modulation of NHR-25 and POP-1 activities through direct contacts of NHR-25 with the distinct β-catenins WRM-1 and SYS-1. The *C. elegans* gonad thus serves as a unique example of how evolutionarily conserved interplay between nuclear receptor and β-catenin signaling pathways governs developmental cell fate decisions. Supported by projects Z60220518, Czech Acad. Sci., and 6007665801 from the Czech Ministry of Education.

# Research article: Characterization of hormone binding to the ecdysone receptor

**DOI:** 10.1673/031.007.1304

**Published:** 2007-03-08

**Authors:** A Azoitei, M Spindler-Barth

**Affiliations:** Department of General Zoology and Endocrinology, University of Ulm, Germany.

Three isoforms of the ecdysteroid receptor (EcR) with different AB domains are described for *Drosophila*. The isoforms are functionally not equivalent and can not replace each other during development (Bender et al. 1997. Cell 91: 777–788). Ligand binding is not influenced by the different AB domains of EcR isoforms significantly, but is modified by the heterodimerization partner, Ultraspiracle (Usp). As reported previously the ligand binding domain of Usp, increases ligand binding to EcR about 90 fold (Grebe et al. 2004. Insect Biochem. Molec. Biol. 34: 982–989). Scatchard plot analysis of ligand binding data show that the B-domain of Usp reduces affinity of ponasterone A to EcR/Usp modestly (about 2–4 fold) and the DNA binding domain impairs ligand affinity to EcR considerably (about 3–8 fold). Replacement of the AB domain of Usp with the activation domain of VP16, which is routinely used in transactivation studies, has no effect on ligand binding of the heterodimeric complex compared to wild type Usp. Some hormone-induced biological responses of EcR / Usp involve the DNA binding domain (DBD) of Usp, whereas others are already observed in the absence of a functional Usp-DBD (Ghbeish et al. 2001. Proc. Acad. Sci USA 98: 3867–3872). We therefore tested the influence of DNA on ligand binding. Monomeric hsp27 used for studies of receptor DNA interaction by gel mobility shift assays has no significant effect on hormone binding, whereas pentameric hsp27 routinely used for transactivation studies increases ponasterone A affinity to the heterodimeric complex considerably depending on the EcR isoform. For comparison of the activity of EcR isoforms determination of receptor concentrations is mandatory to ensure that the same amount of receptor is responsible for the observed effects. Normalization of receptor concentrations determined by Scatchard plot and Western blot do not coincide with ß-galactosidase activity, which is routinely used for normalization of transactivation capability. This means that determination of transfection efficiency by ß-galactosidase does not reflect the concentration of the expressed receptor proteins, presumably due to differences in the turnover and the stability of receptors and ß-galactosidase.

# Research article: Biosynthesis of phytoecdysteroids in spinach

**DOI:** 10.1673/031.007.1305

**Published:** 2007-03-08

**Authors:** A Bakrim, A Maria, R Lafont, N Takvorian

**Affiliations:** ^1^ Université Pierre et Marie Curie, Laboratoire Protéines: Biochimie Structurale et Fonctionnelle, CNRS FRE 2852, 7 Quai St. Bernard, CC29, 75252 Paris 05, France.; ^2^ Université Abdelmalek Essâadi, Faculté des Sciences et Techniques, Laboratoire de Biologie Appliquée, P.O Box 416, Tangier 90 000 Morocco.

Many plant species produce insect molting hormones (phytoecdysteroids - PEs). There is increasing evidence that PEs represent a chemical defence of plants against non-adapted insects and nematodes. The wide distribution of PEs-containing species in the plant kingdom (Dinan L. 2001a. *Phytochemistry* 57: 325–339), the presence of significant PE levels in some specimens of Arabidopsis (a non accumulating plant) (Dinan L. 2001b. *Cell Mol. Life Sci*. 58: 1121–1132), and the ability the maize, a non-accumulating plant species to synthesize some 20-hydroxyecdysone (20E) from labeled precursors (Devarenne T.P et al.1995. *Phytochemistry* 40: 1125–1131) support the idea that most, if not all plant species have the genetic ability to produce PEs, but that the biosynthetic pathway is not active. Most crop species do not accumulate PEs (as a consequence of their selection for a higher yield?). We believe that an environmentally safe approach to crop protection against phytophagous insects could be based on increasing their PE levels, by modifying the activity of genes involved in the control of PE biosynthesis. The achievement of this goal requires a better understanding of the PE biosynthetic pathway and of its regulation. Unfortunately, our current knowledge of this pathway in plants is very limited. We developed a research programme on the PE biosynthetic pathway and its regulation in spinach, one of the few accumulating crop species. We have performed labelling experiments with excised spinach leaves at different ontogenetic stages (or sub-cellular fractions of them) using both very early (mevalonic acid) or late (3-oxo-ketodiol, 2-deoxy-ecdysone, 2-deoxy-20E, ecdysone) 20E precursors. The latter are classically used with Arthropods (Gilbert LI et al. 2002. *Annu. Rev. Entomol*., 47: 883–916). Our results confirm earlier findings that young leaves are unable to produce PEs and accumulate those produced by older ones (Grebenok RJ, Adler JH. 1991. *Phytochemistry* 30: 2905–2910). The metabolic activity of leaves shows large fluctuations in relation with the substrate used and with their developmental stage. They point out major differences with Arthropods regarding both the sub-cellular localization of enzymes and the probable sequence of the reactions (e.g. the possible involvement of 20-hydroxylation at an early stage). The mechanisms regulating PEs biosynthesis were investigated further by excised leaf labelling experiments after removal of the young apical leaves (i.e. sink organs) or after loading with large amounts of 20E.

# Research article: Transcriptional activity of the *Drosophila melanogaster* EcR/USP heterodimer in a heterologous cell culture system

**DOI:** 10.1673/031.007.1306

**Published:** 2007-03-08

**Authors:** JM Beatty, J Callender, C Weinberger, VC Henrich

**Affiliations:** ^1^ Biotechnology and Genomic Research Center, University of North Carolina-Greensboro, Greensboro, NC 27402.

*Drosophila melanogaster* ecdysone receptor (EcR) isoforms and Ultraspiracle (USP), when transfected and expressed in Chinese hamster ovary (CHO) cells, confer these cells with ecdysteroid-inducible transcriptional activity which can be measured by a luciferase reporter gene under the control of a tandemly repeated, canonical *hsp27* ecdysone response element. This capability has formed the basis for a series of experiments that demonstrate that the three EcR isoforms and specific structural modifications of USP, result in altered activities that reveal specific receptor subfunctions. Site-directed mutagenesis has further demonstrated the presence of specific regulatory activities and led to hypotheses for subsequent in vivo tests. These experiments have further demonstrated that juvenile hormone (JHIII) potentiates ecdysteroid responsiveness, that is, it reduces the dosage of ecdysteroid necessary for a maximum transcriptional response by about tenfold. Further, a variety of intermediates in the insect mevalonate pathway from farnesyl diphosphate to JHIII also display potentiation activity. Several JH analogues, including methoprene and pyriproxyfen also potentiate this response, as do some insecticides and phytochemicals implicated as JH mimics by previous studies. From these experiments, it is suggested that JH and other compounds interfere with growth by disrupting the normal control of isoprenoid synthesis by a mechanism that is mediated by the ecdysteroid receptor.

# Research article: Interspecies and isoform comparison of ecdysteroid receptor activity in a heterologous cell culture system

**DOI:** 10.1673/031.007.1307

**Published:** 2007-03-08

**Authors:** JM Beatty, C Weinberger, T Ogura, T Soin, G Smagghe, Y Nakagawa, VC Henrich

**Affiliations:** ^1^ Biotechnology and Genomic Research Center, University of North Carolina-Greensboro, Greensboro, NC 27402.; ^2^ Division of Applied Life Sciences, Graduate School of Agriculture, Kyoto University, Kyoto 606-0022, Japan; ^3^ Laboratory of AgroZoology, Department of Crop Protection, Ghent University, Coupoure Links 653, B-9000 Ghent, Belgium.

Previous studies in a mammalian cell culture system have demonstrated that the three natural isoforms of the *Drosophila melanogaster* ecdysone receptor (EcR) exhibit differences in transcriptional activity via the canonical *hsp27* ecdysone response element when tested with either vertebrate retinoid X receptor or *Drosophila* Ultraspiracle (USP) as a heterodimeric partner. Further, modification of EcR and USP by site-directed mutagenesis or the deletion of specific domains in these nuclear receptors reveal that the composite activity of the ecdysteroid receptor is comprised of numerous and specific subfunctions. The experiments reported here will systematically compare the activity, biochemical properties, and responsiveness to ecdysteroids and juvenoids of the *Leptinotarasa decemlineata* (Colorado potato beetle) ecdysteroid receptor with those of *D. melanogaster* in the same system. This Coleoptera species encodes two known isoforms of EcR, A and B, along with a single form of USP. Interestingly, the USPs of *L. decemlineata*, other Coleoptera species, and other primitive insect orders show greater overall similarity with the ligand-binding domain of the vertebrate RXR than with the equivalent Dipteran USP domain. Previous studies have indicated that RXR and USP differ considerably in their role as a heterodimeric partner for *D. melanogaster* EcR, suggesting that the modulatory role of these homologues is important for overall ecdysteroid responsive transcriptional activity. The potential significance of these functional differences and the implications of these characteristics for insecticidal targeting will be addressed.

# Research article: Functional studies of RXR and USP using transgenic fruitflies *Drosophila melanogaster*

**DOI:** 10.1673/031.007.1308

**Published:** 2007-03-08

**Authors:** Y Beck, T Iwema, G Richards, IML Billas, D Moras

**Affiliations:** Departement de Génomique et de Biologie Structurales, IGBMC, CNRS/INSERM/Université Louis Pasteur, B.P. 10142, 67404 Illkirch Cedex.; * Present address: The International Human Frontier Science Program Organization, B.P. 10034, 67080 Strasbourg Cedex

The fruitfly *Drosophila melanogaster* is a model organism for which a complete genome sequence is available. Due to its well-known genetics, this animal represents an ideal model organism for the study of various developmental processes and for monitoring temporal and spatial gene activation patterns. We have started a project that aims at getting further insight into the ligand binding properties of retinoid X receptor (RXR) and the Ultraspiracle protein (USP), the ubiquitous heterodimerization partner of nuclear receptors such as the vitamin D receptor or the ecdysone receptor. While ligands have been identified for the vertebrate RXRs, the identification of ligands for USP is still a highly debated matter. We have established transgenic fruitflies *Drosophila melanogaster* that express a fusion of the GAL4 DNA binding domain and the ligand binding domain (LBD) of USP or RXR, combined with a GAL4-dependent GFP reporter gene. The pattern of GFP expression upon ligand activation are recorded for different USP LBDs and compared to human RXR. Results of these in vivo experiments will be discussed in light of structural data and compared to in vitro assays.

# Research article: Functions of the ecdysone receptor isoform-A in the hemimetabolous insect *Blattella germanica* revealed by systemic RNAi *in vivo*

**DOI:** 10.1673/031.007.1309

**Published:** 2007-03-08

**Authors:** X Bellés, J Cruz, D Mané-Padrós, D Martín

**Affiliations:** Department of Physiology and Molecular Biodiversity, Institut de Biologia Molecular de Barcelona (CID, CSIC), Jordi Girona 18–26, 08034 Barcelona, Spain.

The molecular basis of ecdysteroid function during development has been analyzed in detail in holometabolous insects, especially in the fruit fly, *Drosophila melanogaster*, but rarely in hemimetabolous species. Therefore, we have cloned the homologue of the *D. melanogaster* ecdysone receptor isoform A (EcR-A) in the hemimetabolous species *Blattella germanica* (the German cockroach), naming it BgEcR-A. The characterization of the developmental expression profile shows that BgEcR-A mRNA is present throughout the penultimate and last nymphal instars in the three tissues analyzed: prothoracic gland, epidermis and fat body. In vitro studies have shown that 20 hydroxyecdysone (20E) has no effect upon the expression of BgEcR-A in incubated portions of epidermis plus adhered fat body tissue from last instar female nymphs. To investigate the functions of BgEcR-A in the German cockroach, we reduced its expression using systemic RNAi in vivo. Accordingly, double strand RNA targeted to the A/B region specific of BgEcR (dsBgEcR-A) was injected in freshly emerged last nymphal instar females. A total of 58% of the dsBgEcR-A treated specimens were unable to molt and showed a *double mouthooks*-*like* phenotype, with duplicated ectodermic structures (like in the mandibles or the laciniae, for example), as occurs in *D. melanogaster* mutants of genes involved in steroidogenesis, like *molting defective* and *ecdysoneless*. In addition, the BgEcR-A knockdown specimens had lower circulating ecdysteroid levels. This can be associated to the fact that the same specimens showed reduced levels of BgE75A mRNA, a nuclear receptor belonging to the genetic cascade triggered by 20E which, at least in *D. melanogaster,* promotes ecdysteroid synthesis. Finally, the dsBgEcR-A treated nymphs showed defects in cell proliferation in the follicular epithelium of nymphal basal oocytes. Given that 42% of BgEcR-A knockdown nymphs were able to complete the imaginal molt, this allowed us to study the functions of BgEcR-A in the adult. The features of these specimens indicate that BgEcR-A is required for adult-specific developmental processes such as wing development, prothoracic gland degeneration and choriogenesis.

# Research article: Analysis of ecdysteroids in eggs of the mealworm: effect of two insect growth regulators

**DOI:** 10.1673/031.007.1310

**Published:** 2007-03-08

**Authors:** H Berghiche, M Houamria, G Smagghe, N Soltani

**Affiliations:** ^1^ Laboratoire de Biologie Animale Appliquée, Département de Biologie, Faculté des Sciences, Université d'Annaba, 23000-Annaba, Algéria.; ^2^ Laboratory of Agrozoology, Department of Crop Protection, Faculty of Bioscience Engineering, Ghent University, B-9000 Ghent, Belgium

RH-0345 (halofenozide), a bisacylhydrazine derivative, is a nonsteroidal ecdysteroid agonist that mimics the action of the moulting hormones, while KK-42, an imidazole compound, is a potent inhibitor of ecdysteroid biosynthesis. Previous results suggested that the reduction of ecdysteroid titer, leading to a reduction of reproductive capacity in *Tenebrio molitor*, is due to a direct and rapid action of KK-42 on ecdysteroid biosynthesis. Moreover, RH-0345 was found to increase the ecdysteroid production and to restore partly the effects on reproductive events induced by KK-42. Therefore, the present study evaluates these two insect growth regulators (IGRs) on egg ecdysteroids in mealworms. The IGRs were applied topically (10 µg/insect) on newly emerged adult females. A qualitative and quantitative analysis of ecdysteroids from pooled freshly laid eggs was made by an enzyme-immunoassay (EIA) using two specific antibodies, the rat monoclonal EC 19 antibody showing a high affinity for 20-hydroxyecdysone (20E) and the rabbit polyclonal B antibody with a strong affinity for ecdysone (E). EIA measurements revealed the presence of two main hormones in control and treated series: E and 20. E was the major hormone in all extracts from control and treated series (75–95 %). In addition, the conjugated ecdysteroids were predominant in mealworm eggs. Halofenozide increased the amounts of both total and free ecdysteroids and 20E. Moreover, KK-42 reduced the amounts of total ecdysteroids and increased the amount of free ecdysteroids, as compared to control series. However, KK-42 had no significant effect in the hormonal composition.

# Research article: Differences in nuclear export determine intracellular localization of ecdysteroid receptor and Ultraspiracle

**DOI:** 10.1673/031.007.1311

**Published:** 2007-03-08

**Authors:** K Betanska, M Spindler-Barth, K Spindler

**Affiliations:** Department of General Zoology and Endocrinology, University of Ulm, Germany.

The heterodimer of the ecdysteroid receptor (EcR) and Ultraspiracle (Usp) is considered as the functional ecdysteroid receptor, which binds to the ecdysone response elements on DNA and modulates the activity of hormone dependent genes. The intracellular distribution of EcR and Usp was investigated: Usp is localised almost exclusively in the nucleus, whereas EcR is observed in the nucleus and cytoplasm (Nieva C et al. 2005 Biol. Chem. 386: 463–70). Here we analysed the mechanisms, which regulate intracellular trafficking of these nuclear receptors. Co-immunoprecipitation of EcR and Usp with transport proteins were performed in vertebrate cell lines, which do not contain endogenous EcR and Usp. EcR and Usp interact with karyopherin α / Rch-1 also in the absence of the heterodimerization partner, which indicates that both receptors can be transported into the nucleus independently. We showed also that nuclear export of EcR, but not Usp involves binding of exportin-1 / CRM1, which is in accordance with the fact, that EcR is distributed between cytoplasm and nucleus, whereas Usp is exclusively present in the nucleus. Additional experiments suggest that Usp can weaken the interaction between EcR and exportin-1 and render the ecdysteroid receptor inaccessible to exportin-1, resulting in almost exclusively nuclear localisation of the heterodimeric complex. The results indicate that different intracellular localization of EcR and Usp is achieved by differences in the nuclear export mechanism.

# Research article: Nucleocytoplasmic shuttling of the ecdysteroid receptor and of ultraspiracle

**DOI:** 10.1673/031.007.1312

**Published:** 2007-03-08

**Authors:** K Betanska, C Nieva, H Ruff, MV Cronauer, K-D Spindler

**Affiliations:** Department of General Zoology and Endocrinology, University of Ulm, Germany.

Import and export of ecdysteroid receptor (EcR) and ultraspiracle (Usp) were investigated in vertebrate cells using fusions with fluorescent proteins. Nucleocytoplasmic shuttling of EcR and Usp are regulated differentially. Both heterodimerization partners can be imported separately, but to different degrees: Usp appears nearly exclusively in the nucleus, whereas EcR is distributed heterogeneously between cytoplasm and nucleus (Nieva et al. 2005. Biol. Chem. 386:463–470). The import of EcR is strongly promoted by Usp and/or by ligand. The import is mediated by karyopherin α (importin α), which forms a complex with EcR and Usp. This import is energy-dependent and can be mediated by the small G-protein RAN, present in the cells. CRM1 (exportin 1) can be coimmunoprecipitated with EcR. Export can be inhibited specifically by leptomycin B. Mutational analysis confirms that export of EcR is exportin-dependent and mediated by helix 3 of the ligand binding domain of EcR. Usp, nearly exclusively localized in nuclei does not interact with exportin. Heterodimerization of EcR with Usp reduces exportin binding to EcR considerably (8 fold), which is in accordance with the predominant nuclear localization of EcR/Usp. EcR and Usp, present in nuclei do not interact with DNA. Hormone or heterodimerization promotes DNA binding, as shown by destruction of Zn-fingers with NO. In conclusion, nuclear localization of EcR is regulated by nuclear export and is modified by heterodimerization with Usp. Interaction with DNA affords hormone or a heterodimerization partner and is mediated mainly by the C- domain of EcR.

# Research article: Structure-function studies of the ecdysone receptor and its partner Ultraspiracle

**DOI:** 10.1673/031.007.1313

**Published:** 2007-03-08

**Authors:** IML Billas, T Iwema, Y Beck, H Nierengarten, C Browning, A Mitschler, D Moras

**Affiliations:** Département de Génomique et de Biologie Structurales, IGBMC, CNRS/INSERM/Université Louis Pasteur, B.P. 10142, 67404 Illkirch Cedex.

The ecdysone receptor EcR is the molecular target of ecdysteroids, the insect and crustacean steroid hormones. EcR functions as a heterodimer with the Ultraspiracle protein USP, the homolog of the retinoid X receptor RXR. The structures of EcR/USP ligand binding domains (LBDs) of the moth *Heliothis virescens* in complex with ecdysteroids and with synthetic bisacylhydrazine ligands will be presented and discussed. Remarkably, we observe an induced fit between receptor and ligand, with different and only partially overlapping ligand binding pockets corresponding to the two classes of compounds. Furthermore, we recently solved the structure of EcR/USP LBDs of a different insect species, the red flour beetle *Tribolium castaneum*. While the LBD sequence of *Tribolium* EcR is closely related to that of *Heliothis* EcR, the LBD sequence of its USP is closer to human RXR than to *Heliothis* USP. Accordingly, the *Tribolium* EcR/USP heterodimer interface is more similar to that observed for vertebrate heterodimers (such as RAR/RXR or LXR/RXR) than to the *Heliothis* EcR/USP interface. Major structural differences are observed for *Tribolium* USP compared to *Heliothis* USP. Functional *in vitro* and *in vivo* assays were undertaken to validate the structural observations. These results on USP have general implications on fundamental aspects of nuclear receptor evolution and the role of RXR and USP as ubiquitous heterodimerization partner.

# Research article: Interaction of EcR/Usp with DNA

**DOI:** 10.1673/031.007.1314

**Published:** 2007-03-08

**Authors:** S Braun, T Fauth, J Beatty, VC Henrich, M Spindler-Barth

**Affiliations:** ^1^ Dept. of General Zoology and Endocrinology, University of Ulm, 89081 Ulm, Germany.; ^2^ Institute for Health, Science, and Society; 209 Forney Building, University of North Carolina Greensboro, Greensboro, NC 27402-6170 USA

Interaction of EcR/Usp with hormone responsive elements is a prerequisite for ecdysteroid dependend transactivation. However transactivtion potency is not in accordance with the affinity of the ecdysone receptor complex to DNA. We studied the interaction of EcR isoforms, Usp variants used in transactivation studies to elucidate the DNA binding of the ecdysone receptor complexes in more detail. We used three different Usp variants, all of them fused to a VP16 activation domain instead of the original Usp AB domain. Usp I contains an additional hexapeptide of the Usp B domain, Usp II consists only of the VP 16 activation domain and C to F domain of Usp, in Usp III the C domain is deleted. The EcR isoforms alone do not interact with hsp27 in the absence of hormone. No band shift is observed with Usp alone. For the EcR isoforms alone in the presence of muristerone A DNA binding is affected differently (A > B1 > B2). All heterodimeric complexes of EcR isoforms and Usp variants bind to DNA even in the absence of hormone. The relative affinities to hsp27 are modulated by the AB domain of EcR and the B-and C-domains of Usp (EcR B2 > EcR A > EcR B1). In the presence of hormone DNA binding is reduced by the hexapeptide adjacent to the C-domain of Usp for heterodimers with all EcR isoforms. Deletion of the C-domain of Usp reduces DNA binding in the presence of hormone for the isoforms B1 and B2 but not for EcR A.

# Research article: Structural studies of EcR/USP bound to bisacylhydrazine ligands

**DOI:** 10.1673/031.007.1315

**Published:** 2007-03-08

**Authors:** C Browning, IML Billas, D Moras

**Affiliations:** Département de Génomique et de Biologie Structurales, IGBMC, CNRS/INSERM/Université Louis Pasteur, B.P. 10142, 67404 Illkirch Cedex.

Two further structures of the heterodimer EcR/USP LBDs of the moth *Heliothis virescens* have been solved in complex with two other bisacylhydrazine insecticide compounds. The structures of EcR/USP in complex with these molecules are similar to the structure of EcR/USP bound to BYI06830 (Billas IML *et al*. 2003. Nature 426: 91–96). Similarly, the binding modes and protein-ligand interactions are comparable, which bring about the structural difference in EcR as compared to the ponasterone A bound HvEcR/USP structure. These two new structures exemplify the evidence that EcR undergoes a remarkable structural modification in order to bind bisacylhydrazine ligan

# Research article: Structural and computational studies of EcR/USP bound to its natural ligand 20-hydroxyecdysone

**DOI:** 10.1673/031.007.1316

**Published:** 2007-03-08

**Authors:** C Browning, C Loch, E Martin, J-M Wurtz, A Dejaegere, R Stote, D Moras, IML Billas

**Affiliations:** Département de Génomique et de Biologie Structurales, IGBMC, CNRS/INSERM/Université Louis Pasteur, B.P. 10142, 67404 Illkirch Cedex.

To date, the EcR/USP LBD heterodimer structure has only been published with the phytoecdysteroid ponasterone A (ponA) (Billas IML *et al*. 2003. Nature 426: 91–96). However, for most insects and arthropods, the active ecdysteroid is 20-hydroxyecdysone (20E) which is identical to ponA, except that it contains a hydroxyl group at the C-25 position. We will present and discuss the EcR/USP LBD heterodimer in complex with the natural ligand 20E. Computational studies were also performed based on the crystallographic structures which then allow to rationalize the lower binding affinity of 20E for its receptor EcR compared to ponA.

# Research article: Intraspecies biochemical variability in ecdysteroid-containing plants *Silene tatarica*

**DOI:** 10.1673/031.007.1317

**Published:** 2007-03-08

**Authors:** IF Chadin

**Affiliations:** Institute of Biology, Russian Academy of Sciences, 28 Kommunisticheskaya Str., Syktyvkar, 167982, Russian Federation.

We carried out investigation of individual variability of *Salene tatarica* on 20-hydroxyecdysone accumulation levels. The several problems of studding plant secondary metabolite individual variability in natural population were resolved. Basic description statistics 20-hydroxyecdysone (20E) accumulation levels in plants of *S. tatarica* population was determined. The correlation between 20E accumulation in flowers and leaves of *S. tatarica* was revealed. Strong correlation between 20E concentrations in generative organs and the mass of this organs was detected. It was shown that there is high 20E level variability even among plants with same stage of growth. Our results well correspond with theoretical model of plant-insect interaction proposed by Karban et al, 1997. This model showed that variability can decrease herbivore performance if herbivore performance is a concave function of the level of resistance. In particular, if herbivores can choose among different plants and plant tissues, then high level of variability may benefit plants under attack and hence may be favored by selection. This research was supported by a grant from the Program of collaboration between Ural and Siberian Divisions of Russian Academy of Sciences, Project N 151.

# Research article: Is the neuropeptide precursor NPLP1 involved in ecdysis?

**DOI:** 10.1673/031.007.1318

**Published:** 2007-03-08

**Authors:** X Chen, P Verleyen, E Clynen, I Mertens, YJ Hua, L Schoofs

**Affiliations:** ^1^ Laboratory of Developmental Physiology, Genomics and Proteomics, KU.Leuven, Leuven, Belgium.; ^2^ Department of Applied Biosciences, University of Zhejiang, Hangzhou, China.

A pioneer peptidomics experiment by Baggerman et al. (2002, JBC, 277:40368–74) revealed novel peptides in the larval central nervous system of the fruit fly, *Drosophila melanogaster.* Three of these peptides, named MTYamide, IPNamide and NAP peptide, belong to the same precursor which was annotated in the flybase as CG3441, and designated as neuropeptide like precursor 1(NPLP1). Since no homologues of NPLP1 are known and the peptides were not sequenced following a traditional purification monitored by a bioassay, there was no indication towards a physiological role for NPLP1. By means of specific antisera the distribution pattern of these 3 NPLP1 peptides in the central nervous system of the fruit fly was studied. All three peptides appear to be present in 28 cells throughout the thoracic and abdominal ganglia (Verleyen P et al. 2004. Journal of Neurochemistry 88: 311–319). Based on this localisation, it became clear that NPLP1 peptides are produced by the 28 so-called Ap-let cells. These Ap-let cells express a unique combination of prohormone converting enzymes, among which PC2. Because animals deficient in this PC2 show an abnormal larval ecdysis phenotype, a role for NPLP1 in ecdysis was proposed (Park DK *et al*. 2004. Developmental Biology 269: 95–108). In addition, mRNA levels of NPLP1 increase significantly before pupariation (Arbeitman MN et al. 2002. Science 297: 2270–75). In order to investigate the role of NPLP1 in ecdysis and/or pupariation, we initiated the identification of the NPLP1 precursor in the flesh fly *Neobelliena* (*Sarcophaga*) *bullata*. This fly is closely related to the fruit fly and because of its larger size an ideal model for research on the physiology of development and metamorphosis. The same species was e.g. used to study the correlated effects of ecdysone and pupariation factors (Zdarek J and Fraenkel G 1969. Proceedings of the National Academy of Sciences USA 64: 565–72). By means of whole mount immunocytochemistry with the 3 antisera raised against the *Drosophila* peptides, we detected an identical distribution pattern in the central nervous system of *Neobelliena*. By affinity purification and mass spectrometry we tried to sequence the 3 *Neobellieria* NPLP1 homologues. Because we obtained only 1 partial sequence, the cloning of the *Neobellieria* NPLP1 precursor was initiated via a RACE PCR protocol. Most part of the cDNA of NPLP1 could be cloned, allowing us to predict and verify the peptides by mass spectrometry. The two most abundant peptides, PQNamide and MGYamide, were synthesized and will be used in a variety of bioassays. At least in the pupariation assay no significant influence could be observed. We plan additional experiments in the future, to establish potential effects of NPLP1 on ecdysis and ecdysone levels.

# Research article: 20-hydroxyecdysone, juvenile hormone and reproduction in *Drosophila virilis* under nutritional stress

**DOI:** 10.1673/031.007.1319

**Published:** 2007-03-08

**Authors:** NA Chentsova, NE Gruntenko, EK Karpova, NV Adonyeva, M Bownes, I.Yu Rauschenbach

**Affiliations:** ^1^ Institute of Cytology and Genetics SD RAS, Novosibirsk, Russia.; ^2^ Institute of Cell and Molecular Biology, University of Edinburgh, Edinburgh, UK

Titre of 20-hydroxyecdysone (20E), metabolism of juvenile hormone (JH), oogenesis and fecundity have been studied under starvation and upon 20E and JH treatment in wild type females of *Drosophila virilis*. A 24-hour starvation has been shown to lead to (i) an increase of 20E titre; (ii) a decrease of JH degradation (an increase of its level); (iii) a drop of the total number of oocytes of stages 8–14; (iv) degradation of early vitellogenic oocytes of stages 9–10; (v) a delay of oocyte transfer through stage 10; (vi) accumulation of mature eggs; (vii) 24-hour oviposition arrest; (viii) a decrease of fecundity for a few days after oviposition resumption. These data suggest that *Drosophila* has a unified mechanism of response of the reproductive system to the action of stress factors of various origins because earlier we showed the same response of *D. virilis* reproductive system to heat stress (Gruntenko N et al. 2003. Insect Mol Biol 12: 393–404). We demonstrate that JH treatment (the hormone application) of fed flies arrests oviposition for 24 hrs, but does not decrease fecundity after oviposition starts again. 20E treatment of fed flies (addition of the hormone to nutrient medium) does not result in oviposition arrest, but decreases fecundity for a few days. These data confirm our previous conclusion (Gruntenko N et al. 2003. Insect Mol Biol 12: 393–404) that an increase of 20E titre under stress results in the degradation of vitellogenic oocytes and a fecundity decrease, while an increase of JH titre leads to oviposition arrest and accumulation of mature eggs. JH application to females prior to starvation has been shown to lead to an increase in fecundity within the first 24 hrs after the end of starvation, which agrees well with the data of Soller M et al. 1999 (Dev Biol 208: 337–351) who showed that JH protected early vitellogenic oocytes from 20E-induced resorption. An experimental rise of 20E titre under starvation has been shown to intensify the negative effect of the nutritional stress on fecundity. The work was supported by RFBR grants ## 04-04-48273, 06-04-48357 and grant for young prominent scientists from Presidium of SD RAS.

# Research article: Regulation of *Schistocerca gregaria* neuroparsin transcript levels by juvenile hormone and 20-hydroxyecdysone

**DOI:** 10.1673/031.007.1320

**Published:** 2007-03-08

**Authors:** I Claeys, G Simonet, B Breugelmans, L Badisco, A De Loof, J Vanden Broeck

**Affiliations:** K.U.Leuven, Department of Biology, Zoological Institute, Laboratory of Developmental Biology, Genomics and Proteomics, Naamsestraat 59, 3000 Leuven, Belgium.

Neuroparsins (NPs) are small proteins that were originally discovered in the *pars intercerebralis corpus cardiacum* neurosecretory complex of the migratory locust brain. NPs are believed to exhibit anti-juvenile, antidiuretic, hyperglycemic, hyperlipemic and neuritogenic activities in the migratory locust, *Locusta migratoria*. From the desert locust, *Schistocerca gregaria*, we recently cloned four different transcripts, each coding for a distinct NP-related peptide. The deduced amino acid sequences range in length between 103 and 107 residues and differ from each other in their carboxyterminal halves. In addition, locust neuroparsins display sequence similarities with related peptides in a variety of other arthropod species, as well as with the conserved N-terminal part of vertebrate IGF-binding proteins (IGFBP) that serves as a protein-protein interaction module responsible for growth factor binding. In addition to the brain, some NP-like precursor (*Scg*-NPP) transcripts also occur in a number of peripheral tissues, and their expression levels are controlled in a gender- and stage-dependent manner. Previous studies revealed a close correlation between NP transcript levels and the gonotrophic cycle. We demonstrate that certain NP transcript levels are significantly altered upon injection of juvenile hormone (JH) or 20-hydroxyecdysone (20E) in adult gregarious desert locusts (five days after final ecdysis). While *Scg*-NPP1 transcript levels did not significantly change as a result of hormone treatment (animals were analyzed 24h after injection), *Scg*-NPP2, *Scg*-NPP3 and *Scg*-NPP4 displayed hormone-dependent regulation in various tissues. *Scg*-NPP2 and *Scg*-NPP3 transcript levels significantly increased in the brain of JH-treated locusts. In addition, JH induction of *Scg*-NPP3 and *Scg*-NPP4 transcripts was observed in male fat body and in male and female gonads. Furthermore, 20E injection also induced *Scg*-NPP2, *Scg*-NPP3 and *Scg*-NPP4 transcripts in desert locust gonads. This is the first time NP-like precursor gene expression is shown in insect ovaries. Our study indicates that the expression levels of some locust NP transcripts are regulated by developmental hormones, suggesting a close correlation between NP expression and the endocrine control of the reproductive cycle.

# Research article: New phytoecdysteroids from cultured plants of *Ajuga nipponensis* with unprecedented functionality

**DOI:** 10.1673/031.007.1321

**Published:** 2007-03-08

**Authors:** J Coll, Y Tandron, XN Zeng

**Affiliations:** ^1^ Departament de Química Orgànica Biològica, Institut d'Investigacions Químiques i Ambientals de Barcelona “Josep Pascual Vila”, C.S.I.C., J. Girona 18, Barcelona, Spain.; ^2^ Laboratory of Insect Toxicology, South China Agricultural University, Wushan Road 483, Guangzhou, China.

The labiatae *Ajuga nipponensis* Makino is widespread in China and is used in sericulture because of its 20-hydroxyecdysone (20E) content. However, large scale extraction in factories with materials collected in different growing locations and seasons, resulted in a varied content of 20E. In some of the analyzed plants 20E was not found either as major neither as minor ecdysteroid. It was also reported then that in order to obtain a sustainable source of plant material (for 20E extraction) a selection of the cultivated variety and appropriate harvesting time appeared necessary. The plant species growing in LIT/SCAU garden was recently reported to contain three major and one minor phytoecdysteroids namely cyasterone, ajugasterone C, cyasterone 22-acetate and 22-dehydrocyasterone, respectively, pointing out a very different profile for this Chinese species. A thorough HPLC analysis of the contents of *A. nipponensis* revealed the presence of three minor bands with the expected ecdysteroid-like UV absorption, along with those previously found. The structures of the isolates were unambiguously elucidated as new naturally occurring phytoecdysteroids based on extensive NMR spectral studies (one and two-dimensional experiments), IR and MS spectroscopy. One of the new compounds, 22-dehydrocyasterone 2-glucoside is just the second example of a C-2 glucosyl derivative. The other two compounds displayed unprecedented acetal functions in the side chain and were named ajugacetalsterone A and B.

# Research article: Is DNA binding important for nuclear localization of the ecdysone receptor?

**DOI:** 10.1673/031.007.1322

**Published:** 2007-03-08

**Authors:** M Cronauer, Ch Tremmel, M Laschak, M Spindler-Barth

**Affiliations:** Department of General Zoology and Endocrinology, Albert-Einstein- Allee 11, 89081 Ulm, Germany.

Intracellular localization of nuclear receptor proteins is the result of continuous nucleoplasmatic shuttling, which may be shifted in favour of the nucleus by interaction with DNA, since DNA-bound proteins may not be fully available to the nuclear export machinery. EcR is distributed between cytoplasm and nucleus, but is quantitatively shifted to the nucleus in the presence of Usp (Nieva C et al. 2005. Biol Chem 386: 463–470). To study the impact of DNA binding on nuclear localization of EcR and Usp. we selectively destroyed the 3-D structure of the C- domain of both receptors with nitric oxide (NO), which is known to remove zinc from the cysteine bonds in a reversible manner, without affecting the amino acid sequence (Kröncke K-D et al. 2000. FASEB J 13: 166–173; Cronauer M et al, submitted). When transfected separately in vertebrate CHO cells in the absence of hormone, EcR and Usp, although present in the nuclei, do not significantly interact with DNA Hormone-binding and/or heterodimerization promote DNA binding. DNA binding of the heterodimeric complex is mediated mainly by the C-domain of EcR. Deletion of the C- domain of Usp is without effect under these conditions. In this study NO was used as an experimental tool only. Its presence and functional importance e.g. during development in *Drosophila melanogaster* rises the question, whether regulation of DNA binding of Zn-finger proteins by NO is also of physiological importance. The tight regulation of NO synthase in insects supports this hypothesis (Kuzin B et al. 2000. Curr Biol 10: 4594–62; Regulski M et al. 2004. Curr Biol. 14, R881–2).

# Research article: Production and purification of polyclonal antibodies against ecdysone receptor (EcR) from the salivary gland of *Bradysia hygida*

**DOI:** 10.1673/031.007.1323

**Published:** 2007-03-08

**Authors:** JAC da Silva, JC de Almeida

**Affiliations:** Departamento de Biologia Celular e Molecular, Faculdade de Medicina de Ribeirão Preto, USP, Brasil.

In *Bradysia hygida* (Diptera, Sciaridae), the salivary glands present three morphologically distinct regions named S1, S2 and S3. At the end of the 4^th^larval instar, in S1 and S3 regions, in about eight different chromosomal sites, gene amplification occurs. This process is triggered by 20-OH ecdysone (20-ecd) and results in DNA puff formation. The amplified genes are activated in two distinct groups, the activity of the first group is dependent on high levels of 20-ecd, while the activation of the second group demands very low hormone levels. So, the salivary glands of *B*.*hygida* constitute an interesting biological model to study the functions of the steroid hormone and its receptors on the processes of gene amplification and control of the amplified genes activity. In our laboratory, we have detected two main transcripts from *BhEcR* gene (5.8 and 1.3 kb) in the salivary glands. In previous studies, we have cloned and partially characterized cDNAs from both transcripts. The minor transcript is noteworthily the more interesting subject, besides its unusual small size, it codes for a small protein that presents the ligand binding domain (LBD) in a position never described in other steroid hormone receptors: in relation to the N terminal of the protein, it comes before the DNA binding domain (DBD). A cDNA from the major transcript (5.8 kb), that includes part of the A/B domain, the entire DNA binding and D domains and part of the LBD, in that order; was cloned into an expression vector. The biotin-tagged truncated ecdysone receptor protein (BhEcR AB^1^/LBD^31^) was expressed in *Escherichia coli* strain JM109 in different culturing conditions. Different approaches for purification were used. Rabbits were injected with the purified BhEcR AB^1^/LBD^31^ for polyclonal antibodies production. The polyclonal antibodies were purified in an immune-affinity chromatography column and used in Western blots and immunocytochemistry for detection of endogenous EcR. On immunoblots of salivary glands from larvae at different ages at the end of 4^th^instar, a band of about 66 kDa was detected (at present, with this lot of antibodies, we could not, yet, to detect a protein that could be the product of the smaller transcript). Immunocytochemistry was performed in whole salivary glands from larvae at age ES (when the 1^st^ group of DNA puffs begins to expand) showing nuclear labeling only in S1 and S3 regions. In this work we describe the synthesis of BhEcR AB^1^/LBD^31^protein in bacteria, its purification and the production and purification of polyclonal antibodies against BhEcR protein. Financial support: FAPESP (proc. 01/13232–0) and FAEPA. J.A.C.S. is a recipient of a PhD fellowship from CNpQ.

# Research article: An unsuspected ecdysteroid/steroid phosphatase activity in the key T-cell regulator, Sts-1: Surprising relationship to insect ecdysteroid phosphate phosphatase

**DOI:** 10.1673/031.007.1324

**Published:** 2007-03-08

**Authors:** L Davies, IP Anderson, PC Turner, AD Shirras, HH Rees, DJ Rigden

**Affiliations:** ^1^ School of Biological Sciences, University of Liverpool, Biosciences Building, Liverpool, L69 7ZB, UK.; ^2^ Department of Biological Sciences, Lancaster University, Lancaster, LA1 4YQ.

In insect females, ecdysteroids are synthesised in the ovarian follicle cells and in many species, it has been shown that much of the hormone is converted into the 22-phosphate conjugate and passed into the eggs. This serves as an inactive storage form of hormone for utilization, following enzymic hydrolysis, during the early stages of embryogenesis, before differentiation of the prothoracic glands. The enzyme catalysing hydrolysis of 22-phosphate conjugate, ecdysteroid phosphate phosphatase (EPP), has been purified and cloned from *Bombyx mori* embryos. This was assigned to a novel class of proteins, but it is shown here that it resides in the large ‘histidine phosphatase’ superfamily related to ‘cofactor-dependent phosphosphoglycerate mutases’ (PGM), a superfamily containing notably diverse catalytic activities. Molecular modelling revealed a plausible substrate-binding mode for EPP. Analysis of genomic and transcript data for a number of insect species show that EPP may exist in both the single domain form previously characterized and in a longer, multi-domain form (UBA, H2 phosphoesterase, SH3, and catalytic phosphatase domains from N-to C-terminus). This latter form bears a quite unexpected relationship in sequence and domain architecture to vertebrate proteins including Sts-1, characterized as a key regulator of T-cell activity. Long form *Drosophila melanogaster* EPP, human Sts-1 and a related protein from *Caenorhabditis. elegans* have all been cloned, expressed in *Drosophila* S2 cells, assayed, and shown to catalyse the hydrolysis of ecdysteroid and steroid phosphates. The surprising relationship considered here between EPP and Sts-1 has implications for our understanding of the function(s) of both.

# Research article: Different Ca^2+^signalling cascades manifested by mastoparan in the prothoracic glands of the tobacco hornworm, *Manduca sexta*, and the silkworm, *Bombyx mori*

**DOI:** 10.1673/031.007.1325

**Published:** 2007-03-08

**Authors:** SG Dedos, D Wicher, H Birkenbeil

**Affiliations:** Saxon Academy of Sciences at Leipzig, Dept. Neurohormones, Jena, Germany.

Measurements of Ca^2+^influx in Fura 2/AM-loaded prothoracic glands (PGs) of the tobacco hornworm, *Manduca sexta*, and the silkworm, *Bombyx mori*, after application of the tetradecapeptide, mastoparan, showed that this peptide can activate dose-dependent increases in intracellular Ca^2+^levels. A previous study [Birkenbeil H. 2000. J. Insect Physiol. 46: 1409–1414] established a link between pertussis toxin-sensitive G proteins and the mastoparan-mediated increase in [Ca^2+^]i levels in the PG cells of M. sexta, but there is no involvement of G proteins in the mastoparan-mediated increase in [Ca^2+^]i levels of PG cells from *B. mori*. Mastoparan could increase the [Ca^2+^]i levels even in the absence of extracellular Ca^2+^in *B. mori* PGs but not in *M. sexta* PGs. Pharmacological manipulation of the Ca^2+^signalling cascades in the PGs of both insects suggests that in *M. sexta* PGs, mastoparan's first site of action is influx of Ca^2+^from plasma membrane Ca^2+^channels while in *B. mori* PGs, mastoparan's first site of action is mobilization of Ca^2+^from ryanodine-sensitive and inositol 1,4,5-trisphosphate (IP3)-insensitive Ca^2+^stores. In *M. sexta*, the combined results indicate the presence of mastoparan-sensitive plasma membrane Ca^2+^channels, distinct from those activated by prothoracicotropic hormone or the IP3 signalling cascade, that coordinate spatial increases in Ca^2+^levels in PG cells. In *B. mori*, considering the ability of mastoparan to bind and modulate glycogen phosporylase that is associated with the ryanodine receptor (RyR; Hirata Y et al., 2003. Biochem. J. 371: 81–88), we propose that mastoparan mediates Ca^2+^mobilization from ryanodine-sensitive and IP3-insensitive intracellular Ca^2+^stores of PG cells leading to specialized Ca^2+^homeostasis in the PGs of *B. mori*.

# Research article: The humoral control of growth by ecdysone and insulin signalling pathways

**DOI:** 10.1673/031.007.1326

**Published:** 2007-03-08

**Authors:** R Delanoue, M Slaidina, S Layalle, P Léopold

**Affiliations:** CNRS/University of Nice, UMR 6543, Pare Valrose, 06108 Nice cedex 2, France.

The control of growth at the level of a whole organism involves a series of intricate humoral regulations allowing the coordination of growth programs in all tissues. One key feature of this global control is the integration of extrinsic influences, like nutrition, as well as intrinsic mechanisms that are linked to the program of development. Our research utilizes *Drosophila* to unravel the mechanisms that link organismal growth to these different parameters. More specifically, we focus on the links between the timing of development and the speed of growth or growth rate. Growth rate is controlled by Insulin/IGF signalling (US) in most metazoans, whereas steroid hormones serve as important developmental timers. Ecdysone plays a key role in timing the different developmental transitions during *Drosophila* development. We recently demonstrated that, in addition to its role as a developmental timer, ecdysone also controls animal growth rate through an antagonistic interaction with IIS, involving changes in dFOXO localization as well as increased transcription of one of its downstream target, *4EBP,* which encodes a translational repressor. We also showed that the larval fat body, a functional equivalent of the vertebrate liver and fat tissue, is a key target of the ecdysone signal, acting as a relay element for ecdysone-dependent growth inhibition. Our goal is to understand the crosstalk between these two hormonal signalling pathways in linking growth and metabolic rates with the developmental timing. We will present some of our recent work defining specific involvement of the various organs participating in this regulatory network.

# Research article: Molecular basis of juvenile hormone action in *Drosophila melanogaster*

**DOI:** 10.1673/031.007.1327

**Published:** 2007-03-08

**Authors:** E Dubrovsky, V Dubrovsky, E Berger

**Affiliations:** Department of Biology, Dartmouth College, Hanover, NH 03755, USA.

Juvenile hormone (JH) is an important regulator of both insect development and reproductive maturation. Although discovered over 70 years ago, the mechanism of JH action remains unknown. Previously we have identified a group of genes, whose expression is induced by JH in *Drosophila* cultured cells. One of those genes encodes a nuclear hormone receptor, *E75A*. It is the first known transcription factor that is induced directly by JH. The *Drosophila E75A* promoter is a primary JH target, it is extremely sensitive to low levels of JH, its activation is rapid and shows decent fold of induction. Consistent with JH regulation, *E75A* mRNA levels are reduced in ovaries of the *apterous4* mutant defective in JH secretion. The expression is effectively rescued by topical methoprene application. *E75* hormonal regulation is conserved in other insect species, such as mosquito *Anopheles gambiae* and moth *Manduca sexta*, which suggests a critical role for the E75 protein in JH signaling. Based on studies in *Drosophila* cultured cells, we propose a model that integrates the E75A protein into the JH signaling pathway. In our model, *E75A* is a primary response gene activated either by ecdysone or JH. In the JH signaling pathway, E75A can perform in several functions. First, it can participate in the downregulation of its own transcription through a mechanism that likely involves autorepression. Second, E75A can facilitate the JH inducibility of secondary response genes. And third, E75A can repress ecdysone activation of a group of early genes including the *BR-C* gene. This last function provides insight into a potential mechanism that underlies the JH “status quo” action. Our current studies focus on searching additional regulatory proteins involved in the JH signaling pathway. The completion of the *Drosophila* genome-sequencing project allowed identifying a group of genes encoding the nuclear receptor superfamily members. Many of these genes are expressed in 52 cells. By employing RNA interference, we perform a functional screen for genes whose products are required for JH activation of gene transcription in 52 cells.

# Research article: Role of ecdysteroids in the control of diapause in *Sesamia nonagrioides*

**DOI:** 10.1673/031.007.1328

**Published:** 2007-03-08

**Authors:** M Eizaguirre, C López, C Schafellner, F Sehnal

**Affiliations:** ^1^ Centre R&D de Lleida (Udl-IRTA), Rovira Roure 191, 2S198 Lleida, Spain.; ^2^ Department of Forest and Soil Sciences, University of Natural Resources and Applied Life Sciences, Hasenauerstrasse 38, A-1190 Vienna, Austria; ^3^ Entomological Institute AS CR, Ceské Budejovice, Czech Republic.

Caterpillars of the Mediterranean com borer, *Sesamia nonagrioides,* that develop under long day (16:8 h day:night) conditions pupate at the end of the 5th or 6th instar but those growing at a short photoperiod (12:12 h day:night) enter a larval diapause during which they undergo several extra larval molts. Diapause is apparently caused by elevated juvenile hormone titer (Eizaguirre M et al. 2005. J. Insect Physiol. 51, 1127–1134) and is associated with a change in molting frequency. Under both photoperiods the caterpillars molt in short intervals of 3 – 4 days until the 6^th^ instar that is extended to about 10 days in the non-diapausing and to about 14 days in the diapausing larvae. We examined possible role of ecdysteroids in the control of instar length and diapause maintenance. Ecdysteroid titers of the non-diapausing and diapausing larvae were compared in the 4^th^ and 6^th^ larval instars. No significant difference was discerned in the 4^th^ instar. The titer rose in both non-diapausing and diapausing larvae from a low initial level to a peak on day 2, followed by a decline to a minimum in newly ecdysed 5^th^instar larvae 2 days later. In the 6^th^instar, the titer was initially low but in the non-diapausing larvae it increased between days 5 and 6, i.e. about one day before some of the insects ceased feeding. At the start of larval-pupal apolysis (day 8) the titer rose to over 500 ng 20E equiv./ml and in pharate pupae (day 9) to about 700 ng, but it dropped sharply during the pupal ecdysis. In the diapausing 6^th^instar larvae, ecdysteroids increased only on day 9 to more than 400 ng 20E equiv./ml but declined to about 100 ng in pharate larvae of the 7 instar. Ecdysteroid titer remained low in subsequent instars of the diapausing larvae save for the molt-inducing increases that occurred in long intervals. For example, in the 8^th^instar the ecdysteroids typically rose on days 12 to 14. It should be mentioned that the molt-inducing ecdysteroid increases in the 6^th^ and later instars of the diapausing larvae were paralleled by temporal reduction of the juvenile hormone esterase (JHE) activity. Ecdysteroid titer decline in newly ecdysed larvae was associated with a rise of JHE, indicating a causal relationship. Similar correlation between ecdysteroid titer and JHE activity was detected also in the course of larval molting from the 5^th^to the 6^th^instar. Before pupation, however, JHE activity grows concomitantly with the ecdysteroid increase on day 6, suggesting that JHE changes at the end of the instar (rise before pupation and decline before a larval ecdysis) depend also on the juvenile hormone titer.

# Research article: Profile of ecdysteroids in Mediterranean flour moth: effects of two insect growth regulators RH-0345 and pyriproxyfen

**DOI:** 10.1673/031.007.1329

**Published:** 2007-03-08

**Authors:** I El Ouar, N Aribi, G Smagghe, N Soltani-Mazouni

**Affiliations:** ^1^ Laboratoire de Biologie Animale Appliquée, Département de Biologie, Faculté des Sciences, Université d'Annaba, 23000- Annaba (Algérie).; ^2^ Laboratory of Agrozoology, Department of Crop Protection, Faculty of Biosciences Engineering, Ghent University, B-9000 Ghent (Belgium)

Ecdysteroids and juvenile hormones regulate many developmental and physiological processes in insects and are considered as potential specific target site for pest control. Two insect growth regulators, halofenozide and pyriproxyfen, agonists of ecdysteroids and juvenile hormone respectively, were tested, in simple or combined treatment, during the pupal stage of *Ephestia kuehniella* (Lepidoptera: Pyralidae). These compounds were applied topically (RH 0345, 10 µg; pyriproxyfen, 10 ng on newly ecdysed pupae and evaluated on ecdysteroid profile. Enzyme-immunoassay (EIA) measurements (using a specific antibody, showing a high affinity for 20-hydroxyecdysone (20E)) showed the presence of a single hormonal peak in both sexes, located at day 6. In addition, the female controls present a specific increase of ecdysteroid titers between days 2 and 4. RH-0345 treatment shifts back, the position of the pupal ecdysteroid peak (to day 4) in the males. Whereas, this peak was suppressed when RH-0345 was followed by pyriproxyfen. In females, the RH-0345, suppressed the specific increase of ecdysteroid (between days 2 and 4), but the hormonal augmentation was maintained when the RH-0345 treatment was followed by pyriproxyfen. Treatment with pyriproxyfen increased ecdysteroid titers in both sexes and caused a precocious delay of the common peak and suppressed the specific increase of ecdysteroid in females. Pyriproxyfen followed by RH-0345, shifted the peak from day 6 to 8 in males and suppressed the specific ecdysteroid increase in females.

# Research article: Role of Broad and E75 in early embryogenesis and nymphal development of the milkweed bug, *Oncopeltus fasciatus*

**DOI:** 10.1673/031.007.1330

**Published:** 2007-03-08

**Authors:** DF Erezyilmaz, H Kelstrup, JW Truman, LM Riddiford

**Affiliations:** Department of Biology, University of Washington, Seattle, WA 98195-1800, USA.

Broad, the pupal specifying transcription factor in holometabolous insects, appears transiently in early embryos of the milkweed bug, *Oncopeltus fasciatus*, then reappears during nymph formation in late embryogenesis. Subsequently, *br* is expressed throughout nymphal life, but disappears in the final nymphal instar for the adult molt (Erezyilmaz DF *et al. Proc. Nat. Acad*. *Sci. USA,* 103, 6925–6930, 2006). When *br* RNAi was injected into females, it was incorporated into the eggs and caused posterior truncation of the embryos, with the most severely affected showing only the head with differentiated eyes. *In situ* analysis showed that *br* is expressed transiently as the germ band is moving into the yolk and forming the abdominal segments. To decipher *br*'*s* role in segment formation, we are currently determining whether it is co-expressed with *wingless*,*decapentaplegic*, and other patterning genes that are important in segment addition. To elucidate its role in postembryonic development, we injected *br* RNAi into either 3^rd^ or 4^th^ instar nymphs and found that when given early in the instar, it caused a stationary molt in that the subsequent nymphal instar retained the pigmentation patterns and wing pad proportions of the stage that was injected. The treated animals grew and molted either 3 or 2 times respectively to become adults with normal adult pigmentation but foreshortened and abnormally shaped wings. Thus, Broad is necessary for wing morphogenesis and heteromorphosis during nymphal molting. We also have shown that its expression during nymphal life is maintained by juvenile hormone (JH). The ecdysone-induced transcription factor E75A also appears to have two different roles in embryonic and postembryonic life of *Oncopeltus*. We obtained by PCR the E75A isoform from embryonic day 1 cDNA using oligos to a portion of the DNA-binding domain and then extending the amplified piece by 3′ and 5′ RACE. The A/B domain shows 32% identity and 54% similarity with the lepidopteran E75A domains, the C region 97% identity, and the hinge region 77% identity and 86% similarity with E75. RNAi injections into the females caused formation of embryos that lacked the labium and the second thoracic (T2), third thoracic (T3), and the first abdominal (A1) segments. *In situ* analysis of known segmentation patterning genes showed that the labium forms but apparently is subsumed into T1 and T2 is subsumed into T3 which disappears in the most severely affected; the Ubx and AbdA boundaries are normal in the mildly affected phenotypes. Further analysis of the basis of the disappearance of A1 is underway. Thus, E75A may be regulating genes that stabilize the segmental identity. E75 RNAi injections into 3^rd^, 4^th^ or 5^th^ instar nymphs prevented further molting although the nymphs grew to the normal size for their stage and lived more than 2 months. Initial studies showed that injection of one dose of 20-hydroxyecdysone was insufficient to restore molting, so further studies are currently underway to determine if this lack of E75A leads to decreased ecdysteroid titers for molting in *Oncopeltus* as it does in *Drosophila*. Supported by NIH R01-GM060122.

# Research article: Rapid, nongenomic responses to ecdysteroids and catecholamines mediated by a novel *Drosophila* G-protein-coupled receptor

**DOI:** 10.1673/031.007.1331

**Published:** 2007-03-08

**Authors:** PD Evans, DP Srivastava, V Reale

**Affiliations:** The Inositide Laboratory, The Babraham Institute, Cambridge, CB2 4AT, UK.

Classically, steroid hormones have been thought to mediate their actions by binding to intracellular proteins that migrate to the nucleus and induce changes in gene expression. However, it is now becoming clear that steroids may also induce rapid actions through the activation of various second messenger pathways by cell surface receptors. Considerable controversy exists over the mechanisms underlying these non-genomic effects. Thus, some effects may be attributable to the allosteric actions of steroids on ligand-gated ion channels, such as GABAA receptors or NMDA receptors. Other effects may be mediated via the activation of G-protein coupled second messenger pathways that can change the activity of adenylyl or guanylyl cyclase, modulate the actions of ion channels, increase mitogen-activated protein kinase (MAPK) or phosphoinositide 3-kinase (PI3K) activity, or change intracellular calcium levels. In addition, mechanisms may exist for exposure of conventional nuclear steroid receptor proteins to extracellular signals at the plasma membrane so that they can interact with such G-protein- coupled pathways. However, in a few cases direct actions of vertebrate steroids have been shown with specific G-protein coupled receptors (GPCRs). Thus, progesterone activates a novel cloned GPCR from the sea trout which activates MAPK activity and inhibits adenylyl cyclase activity (Zhu Y et al., 2003. Proc. Natl. Acad. Sci., USA, 100:2231–2236). In addition, estrogen can activate an orphan GPCR, GPR30, in many cell lines and tissues to activate the extracellular signal-related kinase (ERK1/2) via the transactivation of the epidermal growth factor receptor through the release of heparin-bound epidermal growth factor (Filardo EJ et al., 2000. Mol Endocrinol., 14:1649–1660). We have recently cloned and characterized a novel Drosophila GPCR, DmDopEcR (CG018314), which can be activated by both ecdysteroids and catecholamines (Srivastava DP et al. 2005. J. Neuroscience, 25:6145–6155). This receptor shows sequence homology with vertebrate β-adrenergic receptors and is activated by dopamine to increase cAMP levels and to activate the PI3K pathway. Conversely, ecdysone, and to a lesser extent, 20-hydroxyecdysone, show a high affinity for the receptor in binding studies and can inhibit the effects of dopamine, as well as coupling the receptor to a rapid activation of the MAPK pathway. The latter effect appears to be mediated via the transactivation of cell surface growth factor receptors. The receptor may thus represent the Drosophila homolog of the vertebrate “γ-adrenergic receptors” responsible for the modulation of various activities in brain, blood vessels and pancreas. The receptor is highly expressed in nervous tissue in both adults and embryos as well as being transiently expressed in salivary glands and the gut during embryonic development. Thus, DmDopEcR may function as a cell surface GPCR that is responsible for some of the rapid nongenomic actions of ecdysteroids, during both development and signalling in the mature adult nervous system.

# Research article: Modulation of chromatin access for transcription and remodelling factors by cytoskeletal proteins during ecdysone-triggered cell death of *Drosophila* salivary glands

**DOI:** 10.1673/031.007.1332

**Published:** 2007-03-08

**Authors:** R Farkas, S Kucharova-Mahmood, L Medvedova-Mentelova, I Raska, BM Mechler

**Affiliations:** ^1^ Department of Developmental Genetics, Institute of Experimental Endocrinology, Slovak Academy of Sciences, Vlarska 3, 83306 Bratislava, Slovakia; ^2^ Department of Developmental Genetics, Deutsches Krebsforschungszentrum, IMF 280, D-69120 Heidelberg, Germany.; ^3^ Department of Genetics, Faculty of Science, Comenius University, 84215 Bratislava, Slovakia; ^4^ Department of Cell Biology, Faculty of Medicine, Charles University, 128 00 Prague, Czech Republic

At the onset of *Drosophila* metamorphosis the steroid hormone ecdysone activates a cell death program that leads larval salivary glands (SGs) to rapidly disintegrate about 14–16 hr after puparium formation. During this process ecdysone acts through the ecdysone receptor (EcR/Usp) heterodimer that regulates primary response genes, including the *Broad-Complex* (*BR-C*) gene encoding a family of zinc finger-BTB transcription factors, among which the Z1 isoform is critical for SG death. The timing of SG histolysis depends upon the level of p127^l(2)gl^, a cytoskeletal tumor suppressor that interacts with nonmuscle myosin II heavy chain, nmMHC, encoded by the *zipper* (zip) gene. Reduced *l*(*2*)*gl* expression delays SG histolysis whereas over-expression accelerates this process without affecting larval and pupal development. Recent analysis showed that p127 encoded by *l*(*2*)*gl* and nmMHC regulate chromatin access of BR-C Z, E74 and a series of remodeling factors including SIN3, RPD3 and SMRTER. In wild-type SGs, these factors bind to chromatin but in *l(2)gl* SGs they accumulate in the cytoplasm and the cortical nuclear zone (CNZ) and are unable to associate with chromatin. Similar chromatin exclusion can be achieved by overexpression of nmMHC and occurs also in SGs of developmentally delayed *zip^E(br)^*/+ larvae. Screen for phenotype modifiers has revealed genetic interaction between *l*(*2*)*gl* and *BR*-*C*, as well as between *l*(*2*)*gl* and *zip^E(br)^*. Ectopic expression of p127 from *UAS*-*l*(*2*)*gl* transgene fully rescues *l*(*2*)*gl*- phenotype. Using co-immunoprecipitation we have shown that these nuclear factors can form immunocomplex with cytoskeletal proteins, supporting idea that interaction is physical. Thus, p127^l(2)gl^ and nmMHC act jointly to control chromatin access of BR-C Z1, SIN3, RPD3, SMRTER and possibly several other factors, and in this way regulate the early stages of ecdysone cascade leading to SG histolysis. A similar mechanism may occur in *l*(*2*)*gl* tumour cells and can explained the partial genome silencing occurring in these cells. This work was supported by grants APVT-51-027402, VEGA-2/3025/23 and NATO-CRG-972173 and -LST.CLG-977559.

# Research article: The laminarin-induced nodulation reaction in larvae of the flesh fly, *Neobellieria bullata*, is mediated by 20-hydroxyecdysone and juvenile hormone

**DOI:** 10.1673/031.007.1333

**Published:** 2007-03-08

**Authors:** V Franssens, G Smagghe, G Simonet, I Claeys, B Breugelmans, A De Loof, J Vanden Broeck

**Affiliations:** ^1^ Laboratory of Developmental Physiology, Genomics and Proteomics, Department of Animal Physiology and Neurobiology, Zoological Institute, K.U. Leuven, Naamsestraat 59, B-3000 Leuven, Belgium.; ^2^ Laboratory of Agrozoology, Department of Crop Protection, Faculty of Bioscience Engineering, Ghent University, Coupure Links 653 B-9000 Ghent, Belgium.

Insects have a very efficient innate immune system, including humoral and cellular components. The cellular immune responses refer to the hemocyte-mediated processes such as phagocytosis, nodulation and encapsulation. Nodulation, which is considered the predominant defense reaction to infection in insects, is a complex process influenced by various endogenous factors. However, the precise mechanisms underlying nodulation remain largely unkown. In the present study, we examined the influence of the insect hormones 20-hydroxyecdysone (20E) and juvenile hormone (JH) on the laminarin-induced nodulation reaction in larvae of the flesh fly, *Neobellieria bullata*. Treating third-instar larvae of *N. bullata* with 20E prior to laminarin injection enhanced the nodulation response in a dose-dependent manner. However, when the incubation period between 20E and laminarin injection was less than 4 h, 20E did not influence the formation of nodules. The non-steroidal ecdysone agonists, RH2485, RH5849 and RH0345, similarly enhanced the nodulation reaction, although they were less active than 20E, since they required a higher dose and a longer incubation period. In contrast to ecdysone stimulation, supplying larvae with JH or the juvenile hormone analogs (JHA), fenoxycarb and pyriproxyfen, significantly impaired their ability to form nodules in response to laminarin. These findings demonstrate for the first time that 20E and JH play an important regulatory role in the nodulation process.

# Research article: Modulation of ecdysteroid action by juvenile hormone in wing development and larval marking pattern formation in Lepidoptera

**DOI:** 10.1673/031.007.1334

**Published:** 2007-03-08

**Authors:** H Fujiwara, S Lobbia, R Futahashi

**Affiliations:** Graduate School of Frontier Sciences, University of Tokyo, Kashiwa 277-8562, JAPAN.

Ecdysteroid is involved in various developmental processes during molts and metamorphosis. Juvenile hormone (JH) modulates the ecdysteroid-induced cellular responses and developmental fates, although the molecular mechanisms of JH modulation on ecdysteroid actions are largely unknown. To elucidate the mechanisms, we have studied two developmental phenomena; (1) female-specific wing degeneration in the tussock moth, *Orgyia recens* (2) alteration of larval marking patterns in the swallowtail butterfly, *Papilio xuthus*. We found that JH alters the original developmental fates in these ecdysteroid-dependent events, (1) Females of the tussock moth *Orgyia recens* have only vestigial wings, whereas the males have normal wings. We previously found that ecdysteroid induces cell death in whole area of pupal wings in female but only in peripheral region in male. We have studied expression of EcR-A and EcR-B1 mRNAs on *Orgyia* pupal wings by *in situ* hybridization. However, we did not detect a large difference of EcR isoforms between male and female wings to explain well the sex-specific responses. We further tested the effects of juvenile hormone (JH) on cultured pupal wings of *O. recens* and found that the addition of both JH and 20E in culture induced wing degeneration not only in females but also in males. In addition, pre-treatment of higher concentration of JH in the cultured pupal wings of the silkworm, *Bombyx mori* (male and female adult has normal wings), also caused the wing degeneration by the ecdysteroid treatment. These results indicate that JH modulates the ecdysteroid action to induce cell death on pupal wings not only in *O. recens* but also in other Lepidoptera. (2) The larva of swallowtail butterfly *Papilio xuthus* changes its body markings markedly during the fourth ecdysis, from the bird-dropping type to the greenish leave like markings. We found that earlier treatment of ecdysteroid during 4^th^ instar produces the precocious 5^th^ instar larva which marking is similar to the original 4^th^instar larva. The marking patterns by this treatment is gradually altered dependent on the timing during 4^th^instar, suggesting that some factors involved in the marking fate is changed during the 4^th^ instar. We applied juvenile hormone analogs to 4^th^instar and found that JH treatment within 1day after the 3^rd^ecdysis produces the 4^th^instar-like (bird dropping type) 5^th^instar larva after the 4^th^ecdysis. This suggests that JH titer is changing during the 4^th^instar in *P. xuthus* and may determine the marking fate in the early 4^th^instar.

# Research article: Expression analysis of BmSH3, a putative modulator of the function of the ecdysone-regulated orphan nuclear receptor BmE75C, during silkmoth oogenesis

**DOI:** 10.1673/031.007.1335

**Published:** 2007-03-08

**Authors:** Th Georgomanolis, K Iatrou, L Swevers

**Affiliations:** Insect Molecular Genetics and Biotechnology Group, Insitute of Biology, National Center for Scientific Research “Demokritos”, Aghia Paraskevi Attikis, Athens, Greece.

Silkmoth oogenesis occurs principally during pupal and pharate adult development and is controlled by the molting hormone, 20-hydroxy-ecdysone (20E). Studies that employed the use of the ecdysone agonist tebufenozide demonstrated that the initiation of vitellogenesis is dependent on a rise in the titers of 20E while the transition to choriogenesis requires a down-regulation of 20E signaling. In the latter phase of oogenesis, the orphan nuclear receptor BmE75C becomes upregulated at the end of vitellogenesis and the beginning of choriogenesis. In order to clarify the role of BmE75C during the vitellogenesis-choriogenesis transition, yeast two-hybrid experiments were conducted to isolate regulatory factors that might modulate the function of BmE75C. One of the factors that were isolated is BmSH3, a protein with three SH3 domains at the C-terminus and one domain with homology to the sorbin peptide at the N-terminus. Our aim is to clarify the possible role of BmSH3 during ovarian development, more specifically its interaction with BmE75C. Using antibodies that were raised against specific regions of BmSH3, a protein with a mass of approximately 75–85 kDa was identified in Western blot. BmSH3 is expressed in the cells of the follicular epithelium of the ovarian follicles at all developmental stages examined, from early vitellogenesis to late choriogenesis. A peak of expression was observed, during the transition from vitellogenesis to choriogenesis, as proved both by Western and RT-PCR analysis. To study the subcellular localization of BmSH3, immunofluorescence experiments were carried out. BmSH3 is localized in the cytoplasm where it is concentrated at the cellular boundaries. Interestingly, BmSH3 protein is also localized in distinct large spots at the apical site of the follicular cells. Also in Bm5 tissue culture cells transfected with plasmids that express GFP-BmSH3 chimeras, the chimeric protein localizes in the cytoplasm in distinct foci. C- and N-terminal deletion mutants of GFP-BmSH3 show domain-dependent localization patterns. The C-terminus chimeric fragment, containing the SH3 domains, is located diffusely only at the cytoplasm, whereas the N-terminus chimeric fragment, containing the SoHo domain, is located diffusely throughout the cell and at distinct foci in the nucleus. Future studies aim at the elucidation of the functional role of BmSH3 during silkmoth oogenesis, in particular its possible role in the modulation of the function of BmE75C. It can also be noted that mammalian homologs of BmSH3 have been implicated in insulin signaling, cytoskeleton organization and cell-cell or cell-extracellular matrix interactions. BmSH3 could be involved in similar processes during silkmoth oogenesis.

# Research article: Invertebrate-specific effects of endocrine disruptors on molting, embryogenesis, and vitellogenesis in mysids

**DOI:** 10.1673/031.007.1336

**Published:** 2007-03-08

**Authors:** A Ghekiere, N Fockedey, T Verslycke, CR Janssen

**Affiliations:** ^1^ Ghent University, Laboratory of Environmental Toxicology and Aquatic Ecology, J. Plateaustraat 22, B-9000 Ghent, Belgium.; ^2^ Ghent University, Marine Biology Section, Krijgslaan 281/88, B-9000 Gent, Belgium; * Current address: EURAS, Rijvisschestraat 118 bus 3, B-9052 Zwijnaarde, Belgium; ** Current address: VLIZ, Wandelaarkaai 7, B-8400 Oostende, Belgium; ^∘^ Current address: Biology Department MS#32, Woods Hole Oceanographic Institution, Woods Hole, MA 02543, USA.

Investigative efforts on the effects of endocrine disrupters have mainly concentrated on the evaluation of these effects in vertebrates. Significantly less attention is giving to understanding potential environmental disruption in invertebrates. Given that invertebrates account for roughly 95% of all animal species, and are critical to ecosystem structure and function, it is essential to close this gap in knowledge and research The relatively large body of information on arthropod endocrinology makes insects and crustaceans excellent models for evaluating chemically-induced endocrine disruption. Ecdysteroids (molting hormones) and juvenoids (juvenile hormones), represent two classes of hormones in arthropods that regulate many aspects of their development, growth, and reproduction. Environmental chemicals that have the potential to disrupt ecdysteroid and juvenoid signaling may elicit toxicity unique to invertebrates. In this context, we evaluated the effect of the insecticide methoprene, a juvenile hormone analog, on three important endocrine regulated processes: molting, embryogenesis and vitellogenesis in the mysid *Neomysis integer*. First, the effect of methoprene on mysid molting was studied by individually exposing juveniles (<24-h) for 5 succesive molts. In a second series of experiments embryos were removed from unexposed gravid females and exposed to methoprene. The effects on mysid *in vitro* embryogenesis were evaluated until hatching. Finally, we developed a mysid vitellin enzyme-linked immunosorbent assay (ELISA) and quantified the effect of methoprene on vitellogenesis: gravid females containing stage 1 embryos were exposed to the test substance for 96-h. Methoprene at concentrations of 1 to 100 µg/l had significant effects on molting, reproduction and embryogenesis in *N. integer*. We conclude that chemicals designed to disrupt normal ecdysteroid/juvenoid signaling in insects, can have profound effects on growth and reproduction in non-target organisms, while not necessarily affecting vertebrates.

# Research article: The identification and characterization of the Halloween genes of Drosophila: regulation and quantification of expression in Diptera and Lepidoptera leading to the biosynthesis of 20-hydroxyecdysone

**DOI:** 10.1673/031.007.1337

**Published:** 2007-03-08

**Authors:** LI Gilbert

**Affiliations:** Department of Biology, CB 3280, University of North Carolina, Chapel Hill, North Carolina 27599-3280, USA.

It is now more than five decades since Butenandt and Karlson crystallized ecdysone leading to its structural elucidation a few years later, and almost five decades since Clever and Karlson examined the effect of ecdysone on the polytene chromosomes of Chironomus leading to the first proposal that steroid hormones act at the level of the chromosome (gene). Despite those critical and exciting findings, we still do not understand how the insect synthesizes ecdysone from cholesterol i.e. the biosynthetic scheme remains elusive. This major void exists despite a great deal of research, the lack of success likely due to the very low concentration of intermediates and in some cases, their lability. In the last several years we* have readdressed the problem of the biosynthesis of ecdysone and 20-hydroxyecdysone (20E) utilizing: a genetic approach with Drosophila melanogaster; biochemistry (functional genomics); and the fly data base to clone, sequence and then express the four Halloween genes responsible for encoding the P450 enzymes that mediate the last four steps in ecdysteroidogenesis leading to 20E. These genes encode the 22-hydroxlase (disembodied;Cyp302a1); the 2-hydroxylase (shadow; Cyp314a1); the 25 hydroxylase (phantom;Cyp306a1 ) and the 20-hydroxylase (shade;Cyp315a1). Confocal microscopic analysis revealed that these P450S reside in the mitochondria or ER and one, shade, resides in either organelle, perhaps depending on physiological circumstances. This work has now been extended to the Lepidoptera (Manduca and Bombyx) using qPCR to examine changes in gene expression in several tissues including the well-studied prothoracic glands. We are presently investigating the “Black Box”, that reaction or series of reactions leading to the first ecdysteroid-like intermediate, the ketodiol, and believe that this is the key to elucidating the entire biosynthetic scheme. In Drosophila the remaining uncharacterized Halloween gene product is spook, which when mutated results in embryonic lethality due to a very low ecdysteroid titer, as is the case with the four characterized Halloween genes. However, although spook Cyp307a1 is present in the yolk nuclei and amnioserosa as determined by in situ analysis, it is not present in the ring glands of late embryos or third instar larvae. This has been a conundrum for us for several years until our finding that a paralogue of spook exists in the heterochromatin, called spookier (Cyp307a2) which is indeed expressed in the prothoracic gland cells of both the embryonic and larval ring glands. Since no mutants are available for this heterochromatin gene, functional genomic analysis has been slow. It is our feeling, and hope, that spookier acts within the “Black Box” and that its analysis will ultimately lead to the complete elucidation of the ecdysteroidogenic pathway. This work was conducted in collaboration with Michael O'Connor (University of Minnesota), Chantal Dauphin-Villemant (Universite P. and M. Curie), Tetsu Shinoda (National Institute of Agrobiological Sciences), and their colleagues.

# Research article: Biogenic amines act as intermediary between juvenile hormone and 20-hydroxyecdysone in the control of drosophila reproduction

**DOI:** 10.1673/031.007.1338

**Published:** 2007-03-08

**Authors:** NE Gruntenko, NA Chentsova, EK Karpova, AA Alekseev, TN Komarova, M Bownes, I Rauschenbach

**Affiliations:** ^1^ Institute of Cytology and Genetics SD RAS, Novosibirsk, Russia.; ^2^ Institute of Chemical Kinetics and Combustion SD RAS, Novosibirsk, Russia.; ^3^ Novosibirsk Institute of Organic Chemistry SD RAS, Novosibirsk, Russia.; ^4^ Institute of Cell Biology, University of Edinburgh, Scotland, UK

To reveal interaction mechanisms of gonadotropins and biogenic amines in the control of drosophila reproduction, we studied changes in the metabolic systems of dopamine (DA), octopamine (OA), 20-hydroxyecdysone (20E) and juvenile hormone (JH) upon alteration of levels of these four hormones in *D. virilis* and *D. melanogaster*. DA content was increased by feeding flies with DA predecessor, L-DOPA, and decreased by feeding with an inhibitor of DA synthesis, 3-iodotyrosine. The effect of elevated OA content was examined in the flies fed with the amine and the influence of decreased OA content - in the octopamineless mutants *Tβ^hnm18^*(Monastirioti M. 1996. J Neurosci 16:3900–3911). 20E level was hightened by feeding the flies with exogenous 20E and lowered by exposing *ecdysoneless1* mutants to the restrictive temperature, which leads to the arrest of ecdysone synthesis (Audit-Lamour C, Busson D. 1981. J Insect Physiol 27:829–837). The effect of a changed JH content was studied in wild type flies upon application of exogenous JH and in *apterous^56f^* mutants with a drastically decreased JH level (Altaratz M. 1991. Mol Cell Endocrinol 81:205–216). We demonstrate that DA inhibits JH degradation (and apparently increases its titre) in the young females and stimulates JH degradation (decreasing JH titre) in the mature ones. There is a feedback in this regulation: an elevation of JH titre decreases DA content in young females and increases it in the mature ones. The increase of OA content has been shown to rise the titers of 20E and JH (JH degradation goes down); the decrease of OA content gives the opposite effect. We demonstrate that an elevation of 20E titre results in a increase of DA level in young females and its decrease in the mature ones, which in both cases leads to a decrease of JH degradation (an increase of its titre). The fall of 20E titre causes a DA drop in young females and its rise in the mature ones, and JH degradation increase (JH titre decrease) in both. The increase of DA content in young females has been shown to result in a rise of 20E titre, which suggests a regulation mediated by JH (if DA had a direct effect on 20E metabolism by feedback mechanism, 20E titre would have decreased). An increase of 20E titre in young females upon the experimental rise of JH titre confirms this suggestion. Thus the following hormone interactions have been found: (i) OA regulates JH and 20E titres; (ii) DA controls the titres of JH and 20E (indirectly, *via* JH metabolic system); (iii) JH regulates DA content and 20E titre; (iv) 20E controls DA content and JH titre (indirectly, *via* DA metabolic system). Based on the data obtained, we conclude that the above interactions allow to maintain the balance of gonadotropins necessary, according to Seller M et al. 1999. (Dev Biol 208:337–351), for the normal oogenesis. This conclusion has been verified by the demonstration of the effect of 20E on JH-deficit flies (*apterous^56f^*) whose fecundity is drastically lower than that of wild type and oviposition onset is delayed (Wilson T. 1981. Dev Biol 85:425–433). 20E feeding led to a rise of DA content in these mutants and, as a consequence, to a decrease of JH degradation to the wild type level (an increase of JH titre) and to earlier oviposition onset and higher fecundity of young females. The study was supported by RFBR grants ##06-04-48357, 04-04-48273.

# Research article: Autocrine activation of ecdysteroidogenesis in prothoracic glands of the silkworm, *Bombyx mori*

**DOI:** 10.1673/031.007.1339

**Published:** 2007-03-08

**Authors:** S-H Gu

**Affiliations:** Department of Zoology, National Museum of Natural Science, 1 Kuan Chien Road, Taichung, Taiwan, Republic of China.

Ecdysteroidogenesis in the prothoracic glands is activated by neuropeptide prothoracictropic hormone. The present study demonstrated autocrine activation of ecdysteroidogenesis in prothoracic glands of the silkworm, *Bombyx mori*. Using both a long term in vitro organ culture system and ecdysteroid radioimmunoassay, it was found that either decreasing the incubation volume, from 100 to 5 µl, or increasing the number of glands incubated per drop (50 µl) from 1 to 5 significantly increased ecdysteroid secretion. The prothoracic gland-conditioned medium was used to clarify the autocrine factor. The results showed that activation of ecdysteroidogenesis by the prothoracic gland-conditioned medium appears to be dose-dependent and that the dramatic increase in ecdysteroid secretion was observed after 6 h incubation with conditioned medium. The tropic factor was further characterized and it was found that the factor seems to be heat-stable, with its molecular weight being estimated to reside between 1000 Da and 3000 Da. Injection of concentrated putative autocrine factor into day 5 last instar larvae greatly increased ecdysteroidogenic activity of the prothoracic glands as compared with those injected with saline, indicating the in vivo function of the present factor. To my knowledge, this is the first study to demonstrate that prothoracic glands secrete an autocrine ecdysiotropic factor in vitro which has a biological function in vivo. From the similar characteristics of the above factor as the autocrine growth factor reported in the previous study, it was supposed that it may be the same molecular that has both growth-promoting and ecdysiotropic functions.

# Research article: Ecdysone receptor function and structure

**DOI:** 10.1673/031.007.1340

**Published:** 2007-03-08

**Authors:** GN Hannan, L D Graham, A Pawlak, L Noyce, D Tohidi-Esfahani, M Pollard, L Howell, G Lovrecz, L Lu, J Carmichael, P Pilling, WM Johnson, M Bliese, MC Lawrence, RJ Hill

**Affiliations:** ^1^ CSIRO Molecular and Health Technologies, Po Box 184, North Ryde, NSW 1670, Australia.; ^2^ CSIRO Molecular and Health Technologies, 342 Royal Parade, Parkville, VIC 3052, Australia; ^3^ CSIRO Molecular and Health Technologies, Bayview Avenue, Clayton, VIC 3168; ^4^ Department of Biological Sciences, Macquarie University, NSW 2109, Australia

We have cloned and characterised full-length cDNAs encoding both EcR and USP subunits for ecdysone receptors from insect pests spanning three orders: the cotton bollworm *Helicoverpa armigera* (Lepidoptera), the Australian sheep blowfly *Lucilia cuprina* (Diptera) and three members of the Hemiptera, the peach aphid *Myzus persicae*, the silverleaf whitefly *Bemesia tabaci* and the green vegetable bug *Nezara vindula*. The cDNAs have been placed into expression vectors and transfected into CHO and CV1 mammalian cells along with reporter genes functionally linked to ecdysteroid responsive promoters. The ability of the receptors to activate reporter genes in response to insect ecdysteroids and phytoecdysteroids has been studied. The responses of our cloned ecdysone receptors to different ligands will be discussed. Full-length receptor encoding cDNAs have been expressed in an *in vitro* transcription and translation system to examine the protein products of their open reading frames. Regions from within both EcR and USP subunits of our cloned receptors have also been subcloned for expression in a baculovirus system. Studies of the properties of the expressed protein segments, including competition ligand binding *in vitro*, will be described. A new fluorescence polarisation binding assay, suitable for high-throughput screening, has been developed employing sub-cloned receptor segments and a novel fluorescent ecdysteroid conjugate. The three-dimensional structure of the *B. tabaci*, ecdysone receptor heterodimeric ligand-binding domain has been solved by X-ray diffraction (Carmichael JA *et al.* 2005. J. Biol. Chem. 280: 22258–22269). This is the first crystal structure of an ecdysone receptor from a pest insect that is not susceptible to the bisacylhydrazine insecticides. Comparison between this structure and that of the *Heliothis virescens* receptor ligand-binding domain (Billas I *et al*. 2003. Nature 426: 91–96) allows hypotheses relating to the molecular mechanisms underlying the taxonomic order selectivity of these insecticides to be considered.

# Research article: Immunomodulatory properties of selected natural phytoecdysteroids

**DOI:** 10.1673/031.007.1341

**Published:** 2007-03-08

**Authors:** J Harmatha, E Kmonícková, Z Zídek

**Affiliations:** ^1^ Institute of Organic Chemistry and Biochemistry, Academy of Sciences, Laboratory of Natural products. 166 10 - Prague 6, Czech Republic.; ^2^ Institute of Experimental Medicine, Academy of Sciences, Laboratory of Immunopharmacology; 142 20 - Prague 4, Czech Republic.

Effects of phytoecdysteroids on immunobiological responses triggered by lipopolysaccharide and interferon-γ were tested under *in vitro* conditions using murine resident peritoneal macrophages. Namely, production of nitric oxide and secretion of specific cytokines and chemokines were investigated. The series of test agents encompassed ecdysteroids occurring often as major components of the ecdysteroid fraction in plant extracts: 20-hydroxyecdysone, polypodine B, ajugasterone C, ponasterone A and inokosterone. Their structural variability concerns only variation of the number and position of hydroxyls. Two additional ecdysteroids: makisterone A (with a methyl substituent at position 24) and carthamosterone (with oxidised 24-ethyl substituent, fixed to the hydroxyl at position 25, forming a cyclic side-chain lactone). All tested compounds represent natural substances isolated from the medicinal plant *Leuzea carthamoides* (Pis J. et al. 1994 *Phytochemistry* 37: 707–711; Vokác K. 2002. *Collect. Czech. Chem. Commun*. 67: 124–139) and are supposed to be significant for the often reported pharmacological activities of preparations derived from this species (Lafont R, Dinan L. 2003. *J. Insect Sci*. 3:7, online: insectscience.org/3.7; Lafont R et al. 2002. *The Ecdysone Handbook*, online http://ecdybase.org (http://http://ecdybase.org) ). Relation between the molecular structure and immunobiological activity was investigated, and implication of the side-chain moiety was assessed. However, the tested ecdysteroids did not interfere with the immunobiological activity of the immunocompetent cells. Only small activity was recorded in high concentrations of inokosterone and ponasterone A. Thus, our results differ from the information presented at the 14^th^Ecdysone Workshop in Rapperswil (Kholodova Y. 2001 *Ukrainskij biokhimicheskij zhurnal* 73: 21). The efficacy of ecdysteroids was compared with lignans and N-feruloylserotonins from *L. carthamoides*, exhibiting higher immunomodulatory activity (Harmatha J. 2004, In: *Polyphenols Communications 2004*. University of Helsinki, pp. 217). It indicates that phenylpropanoids are more competent for the immunopharmacological feature of the species than ecdysteroids. Supported by GACR grants No. 203/04/0298, No. 305/03/1470, in part also by research projects AV0Z40550506 and AV0Z5008914.

# Research article: Isolation, expression and functional characterisation of a novel ecdysteroid receptor complex from the marine copepod, *Calanus finmarchicus*

**DOI:** 10.1673/031.007.1342

**Published:** 2007-03-08

**Authors:** R Henderson, D Pond, G Smerdon, L Dinan

**Affiliations:** ^1^ Department of Biological Sciences, University of Exeter, Devon, EX4 4QD, UK.; ^2^ British Antarctic Survey, Cambridge, CB3 OET, UK; ^3^ Plymouth Marine Laboratory, Plymouth, PL1 3DH, UK; ^4^ 30 Hederman Close, Silverton, EX5 4HW, UK.

Copepods are small, yet extremely abundant, lower crustaceans found in almost all aquatic environments. The marine copepod *Calanus finmarchicus* is the dominant zooplankton species in the North Atlantic Ocean and plays a key role in the food-chain as the major prey item for many commercially important fish species (Runge JA. 1988. In: Boxshall, G.A., Schminke, H.K. editors, Biology of Copepods. Proceedings 3. International Conference on Copepoda, London (UK), Aug. 1987. Hydrobiologia, vol. 167–168, pp. 61–67). Despite their ecological importance and increased application as a model organism for toxicological testing, practically nothing is known about the endocrinology of copepods. Using published sequence data from other arthropods and molecular cloning techniques, we have isolated the full-length ecdysteroid receptor (*CfiEcR*) and retinoid-X-receptor (*CfiRXR*) cDNA homologues from *C. finmarchicus*. Analysis of the deduced amino acid sequences comprising the conserved DNA and ligand-binding domains (DBD and LBDs) has revealed that the DBD of the CfiRXR shows high similarity to insect USPs, while the LBD most closely resembles vertebrate RXRs. In contrast, the CfiEcR appears to be quite unique. The LBD is similar to insect EcRs, but the DBD does not display the high level of conservation characteristic of any other EcR previously described, providing a valuable insight into the evolution of this receptor in arthropods. In order to demonstrate the functionality of the *C. finmarchicus* receptor complex, we have over-expressed both copepod receptors as His-tagged fusion proteins in *E. coli* and have been able to demonstrate that, as a complex, CfiEcR and CfiUSP bind [^3^H]ponasterone A with high affinity. We have also found that the CfiEcR is capable of interacting with the human RXRα to bind ecdysteroids. By screening a wide range of purified ecdysteroid agonists and antagonists we have been able to assess the binding specificity of this novel copepod ecdysteroid receptor complex. Details of the isolation, expression and binding specificity will be presented.

# Research article: The coordination of the sequential appearance of MHR4 and dopa decarboxylase during the decline of the ecdysteroid titer at the end of the molt

**DOI:** 10.1673/031.007.1343

**Published:** 2007-03-08

**Authors:** K Hiruma, LM Riddiford

**Affiliations:** ^a^ Faculty of Agriculture and Life Sciences, Hirosaki University, Hirosaki 036-8561, Japan.; ^b^ Department of Biology, University of Washington, Seattle, WA 98195-1800, USA

Dopa decarboxylase (DDC) is a key enzyme responsible for sclerotization and cuticular melanization. DDC expression occurs shortly before the last larval molt in the tobacco hornworm, *Manduca sexta*, on the decline of the ecdysteroid titer. During the larval molt as the ecdysteroid titer peaks, MHR3 mRNA appears followed by E75B mRNA. MHR4 mRNA then appears as the titer declines. Previous studies [Hiruma K and Riddiford LM. 2001. Dev. Biol. 232: 265–274) showed that the expression of MHR4 mRNA in the epidermis is directly induced by 20-hydroxyecdysone (20E), but does not occur until a 20E-induced protein(s) disappears. In the *Manduca* GV1 cell line, MHR3 expression was induced by 20E but neither E75B nor MHR4 mRNA appeared. When cells were transfected with MHR3 RNAi, MHR4 mRNA appeared in response to 20E, indicating that MHR3 is one of the 20E-induced inhibitory factors. When pIE^hr^/E75B was transfected into the cells to express E75B constitutively, MHR4 mRNA also appeared in response to 20E. Use of the BacterioMatch II two-hybrid assay showed that E75B forms a heterodimer with MHR3, thereby preventing the inhibitory action of MHR3. The constitutive expression of E75B and MHR4 in the cells suppressed the activation of the 3.2 kb DDC promoter, but 20E had little effect on these actions. Thus, E75B and MHR4 are 20E-induced inhibitory factors that suppress DDC expression and therefore act as ecdysteroid-regulated timers to coordinate the onset of DDC expression at the end of the molt. Supported by the USDA, JSPS and PROBRAIN.

# Research article: Occurrence of phytoecdysteroids in *Microsorum* species (Polypodiaceae) of French Polynesia

**DOI:** 10.1673/031.007.1344

**Published:** 2007-03-08

**Authors:** R Ho, T Teai, D Loquet, J-P Bianchini, P Raharivelomanana

**Affiliations:** Laboratoire de Chimie des Substances Naturelles, Université de la Polynésie Française BP 6570, 98702 Faa'a Tahiti, Polynésie française.

Ferns have always played an important part in the everyday lives of the Polynesians as ornamental and medicinal plants. The use of plants has quite drastically decreased and only some species are still in use. Among them we can find the *Microsorum scolopendria*, better known as ‘metua pua'a’ in Tahiti. This fern belongs to the Polypodiaceae family which is known to contain phytoecdysteroids, and is employed in the medicinal preparations of many Polynesian traditional remedies. This species grows at high and low altitude, on the ground as well as in an epiphytic manner. Six species of *Microsorum* are found in French Polynesia: *M. scolopendria*, *M. punctatum*, *M. maximum*, *M. commutatum*, *M. membranifolium* and *M. rubidum*. Molecules having a biological activity and belonging to the ecdysteroid family have been found in the *Polypodium* genus (former botanical name for *Microsorum*). A chemical survey of the six species of the *Microsorum* genus from French Polynesia has been done to identify and quantify these active molecules. Samples of the six species have been collected from sites mainly located on the island of Tahiti, and then identified. A fraction rich in ecdysteroids has been obtained for each species from their crude extract. The establishment of a chromatographic profile obtained by High Performance Liquid Chromatography makes it possible to evaluate the quantity of ecdysteroid fraction in each six species, and then determine the proportion of the major ecdysteroids, ecdysone and 20-hydroxyecdysone. The results show that most species of *Microsorum* in French Polynesia constitute an excellent source of phytoecdysteroids, especially of ecdysone, which is rarely present as a major component in plants.

# Research article: Crustacean ecdysone receptor and regeneration: EcR/RXR interactions and potential downstream targets

**DOI:** 10.1673/031.007.1345

**Published:** 2007-03-08

**Authors:** PM Hopkins, DS Durica, F Najar, D Kupfer, H Lai, S Lin, B Roe

**Affiliations:** ^1^ Department of Zoology and; ^2^ Department of Chemistry and Biochemistry, University of Oklahoma, Norman, OK 73019, USA.

We identified cDNA clones that encode isoforms of the ecdysteroid receptor (UpEcR) and the retinoid-X receptor (UpRXR) from the fiddler crab *Uca pugilator*. Several UpRXR cDNA splicing variants occurred in coding regions that could potentially influence function. A five-amino acid (aa) insertion/deletion is located in the “T” box in the hinge region. Another 33-aa insertion/deletion is found inside the ligand-binding domain (LBD), between helix 1 and helix 3. Ribonuclease protection assays (RPA) showed that four different UpRXR transcripts UpRXR(+5+33), UpRXR(-5+33), UpRXR(+5-33) and UpRXR (-5-33) - were present in regenerating limb buds. RACE identified the same RXR isoforms in the cDNA libraries for both *U. puglator* and another *Uca* species. UpRXR(-5+33) is the most abundant isoform transcript present in regenerating limb buds in both early regenerating limb buds (blastemal tissues) and late regeneration stages (hypertrophie proecdysial tissues). Expressed crab nuclear receptors were used in EMSA, GST pull down, conventional binding assays, and protein interaction analysis using surface plasmon resonance technology (SPR). EMSA results showed that the UpEcR/UpRXR(-5+33) heterocomplex bound with a series of hormone response elements (HREs). Binding to an IRper-1 HRE occurred only if the UpRXR partner contained the 33-aa LBD insertion. The results of GST-pull down experiments showed that UpEcR would interact only with UpRXRs that contained the 33-aa insertion. Both EMSA and SPR analyses showed that isoforms lacking the 5-aa and 33-aa inserts could homodimerize and interact with a DR-1G HRE. We compared distributions of ESTs derived from the two distinct stages of limb regeneration. Among the most abundant sequences observed during the late proecdysial hypertrophic phase were sequences involved in muscle differentiation and cuticle deposition, including LIM-related proteins, muscle actins and myosin, cuticle proteins, and phenyl oxidase activating factor. Within the distribution of the most abundant sequences in the blastemal library, however, there were a number of transcripts showing homology to factors involved in NFx-B signaling pathways, including TNFRSF1A modulator protein (retinoid-repressible), TGF-β nuclear protein 1, and a number of proteins linked to apoptosis, such as homologues to macropain and cyclophilin. We hypothesize that a TNF signaling pathway is involved in the regulation of pro- and anti-apoptotic signaling during early blastemal development, perhaps subject to crosstalk with retinoid-signaling pathways. Exogenous retinoids affect early regenerating limb bud organization. HPLC, UV absorption, and GC/MS analyses of early blastemas indicated that several retinoids and/or retinoid metabolites were present in this tissue. EST library analysis and biochemical analysis suggest the presence of both cellular retinoid binding proteins and enzymes involved in retinoid metabolism.

# Research article: Oxadiazolines as gene switch ligands

**DOI:** 10.1673/031.007.1346

**Published:** 2007-03-08

**Authors:** R Hormann, O Chortyk, C Thompson, J Friz, D Cress, B Li

**Affiliations:** RheoGene, Inc., 2650 Eisenhower Ave., Norristown, Pennsylvania, 19403, U.S.A.

Ligand inducible gene expression systems (gene switches) have potential applications in human gene therapy and the production of therapeutic proteins. The oxadiazolines are a new class of gene switch ligands for the ecdysone receptor (EcR). A library of 5,5-disubstituted-oxadiazolines has been prepared by solution phase synthesis and assayed in the EcR-based gene expression systems. Representatives of this class induced marker reporter genes at levels several hundred to thirty thousand fold above background with EC50 values in the range of 0.3–5 micromolar.

# Research article: The effect of ecdysone on locomotor activity of the German cockroach, *Blattella germanica*

**DOI:** 10.1673/031.007.1347

**Published:** 2007-03-08

**Authors:** JH Huang, HJ Lee

**Affiliations:** Department of Entomology, National Taiwan University, Taipei, 106, Taiwan.

Circadian rhythms are fluctuations in physiological and behavioral activities that occur over a period of about 24 hours. The German cockroach, *Blattella germanica* L. is a nocturnal species, and its locomotor activities mainly occur during scotophase. Male adults exhibit free-running rhythm under constant darkness and the locomotor activity is higher during the subjective night. For female, the locomotion does not show a circadian rhythm as in male. The locomotor activity is significantly increased a few days before the formation of ootheca. The locomotor circadian rhythm of females is masked by the development of ovaries (Lin TM, Lee HJ. 1996. Chronobiol Int 13: 81–91). Being removed ovaries in the last instar, female adults show free-running rhythm under constant darkness as in male. Furthermore, juvenile hormone is the major hormone controlling the ovarian development, allatectomy not only decreases the locomotion but also eliminates the masking effect (Lin TM, Lee HJ. 1998. J Insect Physiol 44: 1039–1051). The ovary is the major source of circulating ecdysteroids in the adult female German cockroach (Romana I. et al. 1995. Eur J Entomol 92: 93–103). Therefore, the effect of 20-hydrxyecdysone (20E) on locomotion was determined by injection of 20E into adult cockroaches. 20E increased the locomotor activity in both males and females. In addition, the locomotion occurred day and night without circadian rhythm. The result suggested that ecdysone was a masking factor of locomotor circadian rhythm.

# Research article: Nongenomic action of 20-hydroxyecdysone in programmed cell death of *Bombyx* anterior silk gland

**DOI:** 10.1673/031.007.1348

**Published:** 2007-03-08

**Authors:** M Iga, M Iwami, S Sakurai

**Affiliations:** Division of Life Sciences, Graduate School of Natural Science and Technology, Kanazawa University, Kakumamachi, Kanazawa, Japan.

In animal development, tissues that possess stage-specific functions are eliminated through programmed cell death (PCD) after completing their roles. In holometabolous insects, the larval specific tissues undergo PCD at or shortly after the larval-pupal transformation in response to 20-hydroxyecdysone (20E). Such tissues include the intersegmental muscles (Schwartz LM. 1992. J. Neurobiol. 23: 1312–1326) and motoneurons (Streichert LC et al., 1997. Dev. Biol. 183: 95–107) of *Manduca sexta*, the salivary glands of *Drosophila melanogaster* (von Gaudecker B et al. 1974. Cell Tissue Res. 155: 75–89) and the silk glands of *Bombyx mori* (Chinzei Y. 1975. Appl. Ent. Zool. 10: 36–138). The *B. mori* anterior silk gland (ASG) is the nozzle-like organ to spin silk thread from liquid silk proteins produced in the middle and posterior silk glands. After completion of spinning a cocoon, the entire gland degenerates through PCD in prepupal period, which is induced by 20E *in vivo* as well as *in vitro*. Since 20E acts through binding to a heterodimeric ecdysone receptor, EcR/USP, which serves as a transcription factor, 20E-induced PCD has been considered to begin with *de novo* gene expression, although there was no experimental confirmation. This belief was called into question by the results obtained using α-amanitin, a potent inhibitor of RNA polymerase II. α-amanitin prevents 20E-induced PCD when added to the culture of ASGs with 20E from the beginning of the culture, while its addition 8 h after the exposure to 20E does not do so, indicating that the gene transcription needed for the PCD is accomplished by 8 h. Nevertheless, withdrawal of 20E from the culture medium between 8 and 42 h of the culture interferes the progression of PCD sequence (Terashima J et al. 2000. Dev. Genes Evol. 210: 545–558). Based on these previous results, we supposed an involvement of nongenomic action of 20E until 42 h in addition to its genomic action by 8 h. 20E-induced PCD proceeds sequentially through cell shrinkage, nuclear condensation, DNA fragmentation, nuclear fragmentation to apoptotic body formation. A protein synthesis inhibitor, cycloheximide (CHX, 2 mM) induced a cell death that exhibited only nuclear and DNA fragmentation. But the cell morphology was different from 20E-induced PCD. A concentration of 0.2 mM CHX was ineffective at inducing the cell death when added alone, but in the presence of 20E, a cell death similar to that induced by 2 mM CHX was resulted with accompanying nuclear condensation. Since 2 mM and 0.2 mM CHX inhibited protein synthesis equally, the DNA and nuclear fragmentation appear to be mediated by a nongenomic action of 20E. Calcium ionophore was capable of partly mimicking the 20E action when substituted for 20E after pre-culture with 20E for 18 h. Furthermore, inhibitors of PKC and caspase-3 inhibited 20E and CHX- induced cell death. These results suggest a possible involvement of Ca^2+^-PKC-caspase-3 like protease pathway in the nongenomic action of 20E. Here we show that 20E-induced PCD is accomplished through the integration of genomic and nongenomic actions.

# Research article: Interaction of genomic and nongenomic actions of 20-hydroxyecdysone in 20E-dependent development events

**DOI:** 10.1673/031.007.1349

**Published:** 2007-03-08

**Authors:** M Iga, M Sekimoto, M Elmogy, M Iwami, S Sakurai

**Affiliations:** Division of Life Sciences, Graduate School of Natural Science and Technology, Kanazawa University, Kakumamachi, Kanazawa, Japan.

The *Bombyx mori* anterior silk gland (ASG) begin to degenerate through programmed cell death (PCD) in prepupal period in response to 20E. Since 20E acts through binding to a heterodimeric ecdysone receptor, EcR/USP, de novo gene expression has been considered to be the necessary and sufficient condition for inducing PCD. This belief was called into question by the results obtained using α-amanitin, a RNA polymerase II inhibitor. A differential addition of α-amanitin to the culture of ASGs with 20E showed that α-amanitin prevented the PCD progression when added before 8 h of the culture with 20E but not when added at and after 8 h, indicating that the gene transcription needed for the PCD was accomplished by 8 h. Nevertheless, 20E was required for 42 h for PCD completion (Terashima J. et al. 2000. Dev. Genes Evol. 210: 545–558). Based on these previous results, we supposed an involvement of nongenomic action of 20E until 42 h in addition to its genomic action by 8 h. In the non-genomic action of steroid such as estrogen, there are at least two pathways, one through membrane receptor and the other through a nuclear receptor locating beneath or attached to plasma membrane. We showed the presence of membrane-bound ecdysone receptor (Elmogy M et al. 2004. European Journal of Biochemistry 271: 3171–3179), and probably this receptor might be involved in 20E signalling (Elmogy et al.,2006), although its function in the PCD is unclear. Pharmacological studies showed that a possible involvement of Ca^2+^-PKC-caspase-3 like protease pathway in the 20E-induced cell death. These results indicate the single cellular response to 20E, PCD, is accomplished through the cross talk of genomic and non-genomic actions of 20E. Experimental evidence also indicated the presence of a similar non-genomic action of 20E in the 20E-dependent cell cycle in the wing disc cells after pupal commitment in early 5 instar. Accordingly, the non-genomic action of 20E may be widely associated with the 20E action in various 20E-dependent events in insect growth and metamorphosis.

# Research article: Fungal ecdysteroid-22-oxidase: a new tool to control ecdysteroid signaling and insect development

**DOI:** 10.1673/031.007.1350

**Published:** 2007-03-08

**Authors:** M Kamimura, H Saito, Y Kanamori, S Shimura, M Kiuchi

**Affiliations:** National Institute of Agrobiological Sciences, Tsukuba, Ibaraki 305–8634, Japan.

An entomogenous fungus *Nomuraea rileyi* secretes a specific enzyme, ecdysteroid-22-oxidase (E22O), that oxidizes the hydroxyl group at position C22 of ecdysteroids and prevents molting of host insects (Kiuchi M et al. 2003. Arch. Insect Biochem. Physiol. 52: 35–44). We characterized E22O. E22O protein was purified from culture medium of *N. rileyi* using three chromatographic steps by HPLC. Purified E22O had a molecular weight of approximately 76 kDa by gel-filtration and SDS-PAGE analyses, suggesting that E22O is a monomeric protein. The Km values for ecdysone and 20-hydroxyecdysone (20E) were equally about 5 µM, but the Vmax value for ecdysone was 16.5 pmol/min, which was three times greater than that for 20E (5.1 pmol/min). E22O thereby catalyses ecdysone faster that 20E. E22O cDNA was then cloned by RT-PCR and RACE techniques using primers designed based on its partial amino acid sequences. The cDNA encoded a secreted protein and blast search showed that it is a novel FAD-dependent oxidoreductase. Recombinant E22O protein expressed by baculovirus converted ecdysone into 22-dehydroecdysone, indicating that this cDNA truly encoded E22O. When E22O was expressed in *Bombyx* cell lines, they lost responsiveness to 20E monitored by a reporter gene assay using a 20E responsive reporter plasmid. Injection of recombinant E22O protein into the penultimate instar larvae of the silkworm disrupted molting into the last instar. Thus, E22O could be used for control of ecdysteroid signalling and insect development. We are now making transgenic insects expressing E22O. This work was supported by PROBRAIN, Japan.

# Research article: Insight into the ecdysone signaling pathway in the apterygote silverfish and in two primitive endopterygotes

**DOI:** 10.1673/031.007.1351

**Published:** 2007-03-08

**Authors:** B Konopova, M Jindra

**Affiliations:** ^1^ Department of Molecular Biology, University of South Bohemia.; ^2^ Biology Center CAS, Branisovska 31, Ceske Budejovice 37005, Czech Republic.

Our knowledge on function of genes involved in insect ecdysone signaling is mostly based on studies done in the fly *Drosophila* and in lepidopterans, both endopterygote groups that are positioned very close in the evolutionary tree. To understand which components of the ecdysone signaling pathway have been functionally conserved and which have changed roles during insect evolution (e.g. together with the emergence of metamorphosis), it is necessary to study species with more diverse types of development. We chose the primitive apterygote silverfish *Thermobia domestica* (Zygentoma), an insect with ametabolous (ametamorphic) development, and two endopterygotes: the lacewing *Chrysopa perla* (Neuroptera), which belongs to one of the most primitive orders displaying holometaboly, and the red flour beetle *Tribolium castaneum* (Coleoptera). Despite developmental differences, well-conserved components of the ecdysone signaling pathway can be found in these insects. We isolated partial cDNA clones (600–800 bp in length) for the *ecdysone receptor* (*EcR*), *ultraspiracle* (*usp*), *E75* and *broad*-*complex* (*BR*-*C*) genes from *Chrysopa,* and *EcR*, *usp*, *E75*, *BR*-*C*, *ftz*-*fi* and the hormone receptor genes *HR4* and *HR38* from *Thermobia. Tribolium EcR* and usp clones were obtained from Francois Bonneton, *BR*-*C* and *HR38* cDNAs were cloned based on the sequenced genome. We will present data on mRNA expression as well as results of testing each species' susceptibility to RNAi knock down for selected genes. For instance, injection of early *Chrysopa* larvae with double-stranded RNA (dsRNA) against either of the components of the ecdysone receptor complex (EcR or Usp) caused developmental arrest and death of larvae. In contrast, RNAi targeting of *BR*-*C*, which is necessary for pupal development in both *Drosophila* (Diptera) and the silkworm *Bombyx mori* (Lepidoptera), caused no anomalies until the onset of metamorphosis, when the animals were unable to molt into the pupal stage and in more severe cases also failed to spin the cocoon. Preliminary results showed that larval molting could also be disrupted by injection of *EcR*, *usp* or *E75* dsRNA into second to fourth instar *Thermobia* larvae, while *BR*-*C* RNAi allowed development of adults. These data suggest that BR-C is causally linked with metamorphosis as early as in the most primitive holometabolans but that it may play another role in ametamorphic insects. Supported by grant Asooysos from the Czech Academy of Sciences.

# Research article: 20E inhibits lipid peroxidation and maintains membrane fluidity in the brain of *Pyrrhocoris apterus* subjected to oxidative stress

**DOI:** 10.1673/031.007.1352

**Published:** 2007-03-08

**Authors:** N Krishnan, J Vecera, D Kodrík, F Sehnal

**Affiliations:** Institute of Entomology, Czech Academy of Sciences, and Faculty of Biological Sciences, University of South Bohemia, Ceské Budejovice, Czech Republic.

All living organisms harbour defence mechanisms against the challenge of oxidative radicals. In addition to specific enzymes, antioxidant compounds of diverse primary functions are employed. It has been reported that the estrogens of vertebrates and the ecdysteroids of insects also scavenge reactive oxygen species but a role of these hormones in the prevention of tissue damage caused by the radicals has not been documented. We chose to examine effect of 20-hydroxyecdysone (20E) on the brain of adult bugs *Pyrrhocoris apterus* that were injected with the herbicide 1,1′-dimethyl-4,4′-bypyridilium (Paraquat). Paraquat undergoes cyclic redox reactions in the microsomes and mitochondria associated with generation of the superoxide, singlet oxygen, hydroxyl radicals, hydrogen peroxide, lipid peroxides and disulfides. Oxidative insult caused in the bugs by the injection of 38 pmol of paraquat was manifested by a significant elevation of protein carbonyls and of a lipid peroxidation product, the µαλΟνδιαλδεηψδε in brain microsomal fraction. A consequence of lipid peroxidation was a change in the properties of microsomal membranes. Polarisation anisotropy of the membranes examined with the fluorescent hydrocarbon probe 1,6-diphenyl-1,3,5-hexatriene revealed a significant decline in the fluidity of membranes from the brains of bugs subjected to oxidative stress. Another manifestation of the oxidative stress caused by the paraquat was a depletion of the pool of glutathione that is an important component of the antioxidant system. The activity of γ-glutamyl transpeptidase, a key enzyme in glutathione metabolism, was sharply reduced. All these effects indicative of oxidative damage were counteracted by the injection of 20E (1 pmol). The hormone had a profound inhibitory effect on lipid peroxidation and ameliorated changes in the biophysical membrane properties. The formation of protein carbonyls was suppressed, the activity of γ-glutamyl transpeptidase up-regulated, and the level of reduced glutathione enhanced. All these effects were examined in the brain that was chosen because it does not undergo changes in size during the reproductive cycle and in response to ageing. In subsequent experiments we demonstrated a systemic protective effect of 20E against the oxidative stress. We found that paraquat injection caused a slight change in the profile of hemolymph proteins that reflect a sum of metabolic activities of the gut, fat body, gonads, and to lesser extent of other organs. No change in the protein profile occurred in the insect injected with both paraquat and 20E. The role of 20E as a protective agent is therefore not restricted to the brain. The antioxidant function is an important addition to the known roles of ecdysteroids but the mode of this particular 20E action is unknown. The work was supported by grant 522/06/1591 obtained from the Grant Agency of the Czech Republic.

# Research article: Metabolism of [1α,2α-^3^H]-20-hydroxyecdysone in mice

**DOI:** 10.1673/031.007.1353

**Published:** 2007-03-08

**Authors:** S Kumpun, JP Girault, C Blais, A Maria, C Dauphin-Villemant, BE Yingyongnarongkul, A Suksamrarn, R Lafont

**Affiliations:** ^1^ Université Pierre et Marie Curie, Laboratoire Protéines: Biochimie Structurale et Fonctionnelle, CNRS FRE 2852, 7 Quai St. Bernard, 75252 Paris 05, France.; ^2^ Department of Chemistry, Faculty of Science, Ramkhamhaeng University, Bangkok 10240, Thailand; ^3^ Université Paris V - René Descartes, Laboratoire de Chimie et Biochimie Pharmacologiques et Toxicologiques, CNRS UMR 8601, 45 rue des Saints-Pères, 75270 Paris Cedex 06, France.

Ecdysteroids display a lot of pharmacological effects on mammals and humans, most of which appear beneficial (Lafont R, Dinan L. 2003. J. Insect Sci. 3.7 http://www.insectscience.org/3.7/ (http://http://www.insectscience.org/3.7/ ); Klein R. 2004. J. Am. Herbalists Guild, fall/winter 2004, 18–28; Báthori M, Pongrácz Z. 2005. Current Med. Chem. 12: 153–172), but their mechanism of action is far from understood. Whether they act directly and/or through the formation of metabolites is still an open question. In order to investigate the metabolic fate of ecdysteroids in mice, [1α,2α-3H]-20-hydroxyecdysone was prepared and either given orally or injected intraperitoneally to mice. Their urine+feces were collected and the different tritiated metabolites were isolated and identified. The pattern of metabolites is very complex, but does not contain conjugates, as is usual for the less polar vertebrate steroid hormones. Primary reactions involve dehydroxylation at C-14 and side-chain cleavage between C20–C22, thus giving 14-deoxy-20-hydroxyecdysone, poststerone and 14-deoxy-poststerone. These metabolites then undergo several reduction reactions involving in particular the 6-keto-group. The structure of the different metabolites formed will be discussed in the light of the various effects of ecdysteroids already demonstrated on vertebrates.

# Research article: Ecdysteroids from *Chenopodium quinoa*, an ancient Andine crop of high nutritional value

**DOI:** 10.1673/031.007.1354

**Published:** 2007-03-08

**Authors:** S Kumpun, A Maria, N Evrard-Todeschi, JP Girault, R Lafont

**Affiliations:** ^1^ Université Pierre et Marie Curie, Laboratoire Protéines: Biochimie Structurale et Fonctionnelle, CNRS FRE 2852, 7 Quai St. Bernard, 75252 Paris 05, France.; ^2^ Department of Chemistry, Faculty of Science, Ramkhamhaeng University, Bangkok 10240, Thailand; ^3^ Université Paris V, René Descartes, Laboratoire de Chimie et Biochimie Pharmacologiques et Toxicologiques, CNRS UMR 8601, 45 rue des Saints-Pères, 75270 Paris Cedex 06, France

*Chenopodium quinoa* (= quinoa) is a plant cultivated by Inca people from ancient times. The seeds of quinoa have a high protein content with large amounts of essential aminoacids, they are devoid of gluten, and their use is rapidly increasing. The seeds can be eaten after boiling in water instead of e.g. rice, and the flour is used to prepare noodles, muesli. The number of products containing quinoa is in fact rather impressive.

Quinoa belongs to Chenopodiaceae, a family comprising many ecdysteroid-containing species (Dinan L et al. 1998. Biochem. Syst. Ecol. 26: 553–576), among them spinach. Quinoa seeds/flour contain indeed ecdysteroids, among which 20-hydroxyecdysone, makisterone A, 24-epi-makisterone A, 24(28)-dehydro-makisterone A and 20,26-dihydroxyecdysone (Zhu N et al. 2001. J. Agric. Food Chem. 49: 2576–2578) and also a new ecdysteroid, kancollosterone (Dini I et al. 2005. Food Chemistry 92: 125–132). We have performed a detailed analysis of quinoa seeds and flour from various sources, and isolated a lot of minor ecdysteroids together with large amounts of 20-hydroxyecdysone, the latter being present in the range of 250 µg/g seeds. Ecdysteroids remain within seeds after boiling in water, thus eating a portion (50 g) of quinoa provides significant amounts of ecdysteroids [ca. 10 mg of 20-hydroxy-ecdysone, i.e. an amount identical to that of 2 “Ekdysten” pills (Lafont R, Dinan L. 2003. J. Insect Sci. 3/7 http://www.insectscience.org/3.7/ (http://http://www.insectscience.org/3.7/) )].

# Research article: Towards the elucidation of ecdysteroid receptor ligand-binding interactions

**DOI:** 10.1673/031.007.1355

**Published:** 2007-03-08

**Authors:** S Lapenna, RE Hermann, L Dinan

**Affiliations:** ^1^ Department of Biological Sciences, University of Exeter, Exeter, UK.; ^2^ RheoGene Inc., Norristown, Pennsylvania, USA; ^3^ 30 Hederman Close, Silverton, EX5 4HW, UK

Ecdysteroids exert their effects by binding to a specific nuclear receptor complex, formed from the ecdysteroid receptor (EcR) and the ultraspiracle (USP) proteins. Ecdysteroids are good candidates for the regulation of transfected target genes in mammalian systems (gene-switch systems) with potential applications in gene therapy. For such application, non-toxicity of all gene-switch components is an essential requirement. Preliminary toxicity studies have indicated that ecdysteroids are compounds of very low toxicity in mammalian systems and that the search for highly potent ecdysteroids would contribute to limiting any potential toxic effects. Gaining knowledge of which are the key ligand-receptor binding interactions is crucial for designing potent inducers for gene-switch technology. In this work, the binding roles of the various ecdysteroid hydroxyl groups have been investigated by synthesis of a variety of 20-hydroxyecdysone (20E) and ponasterone A (PoA) methyl ether analogues and assessment of their biological activities using the *Drosophila melanogaster* BII bioassay (Clément CY. et al. 1993. Insect Biochemistry and Molecular Biology, 23: 187–193). Since methyl ether derivatives are able to take part in H-bond interactions only as H-bond acceptors, but not as H-bond donors, the resulting changes in biological activity were used to indicate the H-bond donor/H-bond acceptor role of each particular hydroxyl group derivatised. It was found that methylation at 2-, 3-, 14- or 25-OH decreased activity to various extents, with respect to the parent compound, while 20E 22-methyl ether retained activity. The ecdysteroid methyl ethers were used to generate an improved quantitative structure-activity relationship (QSAR), using Comparative Molecular Field Analysis (CoMFA) and Comparative Molecular Similarity Indices Analysis (CoMSIA), which benefits from the availability of an ecdysteroid dataset including 167 compounds, obtained by homogeneous activity assessment by the D. melanogaster BII bioassay and modelled conformations derived from the *Heliothis virescens* EcR-bound PoA crystal structure (Billas IML et al. 2003. Nature 426: 91–96). A model based on CoMFA steric and CoMSIA H-bond acceptor fields could be generated with high predictive power tested on an independent test set of 20 compounds (training set q2=0.515, test set r2=0.689). The generated QSAR model is based on the most extensive ecdysteroid dataset so far collected and its results will be discussed in the light of the experimental activity measures available and hypotheses of binding roles of the ecdysteroid hydroxyl groups will be proposed.

# Research article: Functional characterization and bioinformatics analysis of the *BhC4-1* ring gland enhancer in transgenic *Drosophila*

**DOI:** 10.1673/031.007.1356

**Published:** 2007-03-08

**Authors:** TM Malta, JC Ruiz, N Monesi

**Affiliations:** ^1^ DACTB, FCFRP, University of São Paulo, Ribeirão Preto, SP, Brazil.; ^2^ DBCMBP, FMRP, University of São Paulo, Ribeirão Preto, SP, Brazil.

DNA puffs are formed in sciarid salivary gland polytene chromosomes late in the fourth larval instar, before the pupal molt. DNA puff genes are amplified and transcribed in the salivary gland in a developmentally regulated manner, during the fourth larval instar, as a late response to the hormone ecdysone. We are currently characterizing the mechanisms which control the expression of the *BhC4-1* gene from DNA puff C4 of the sciarid *Bradysia hygida*. At present it is not possible to transform sciarids, and functional studies of sciarid genes are performed in *Drosophila*. The mechanisms that control *BhC4-1* expression in the prepupal salivary glands are conserved in *Drosophila.* The analysis in transgenesis revealed that the *BhC4-1* gene is also expressed in a developmentally regulated manner in the ring gland. The *cis*-regulatory elements which drive gene expression in the salivary gland are located in a 129 bp (-1867/-58) fragment and are separable from those controlling expression in the ring gland, which are located in a 67 bp (-253/-187) fragment. To extend the characterization of the ring gland enhancer, five constructs (MUT 1–5), containing each a distinct 10 bp cluster of mutated sequences in the 67 bp fragment, were cloned upstream the *Fbp1* minimal promoter, in the pCaSper-AUG-β-gal vector. At least five independent transgenic lines were obtained for each construct. Promoter activity was assayed employing a histochemical assay for β-galactosidase in embryos, first instar larvae (L1) and third instar larvae (L3). In embryos and L1, the mutations MUT 4 and MUT 5 abolish gene expression in the ring gland, whereas expression is detected in the ring gland of the MUT 1, MUT 2 or MUT 3 series at these developmental times. In L3, the mutations MUT 3, MUT 4 and MUT 5 abolish reporter gene expression in the ring gland, whereas the mutations MUT 1 and MUT 2 lead to an apparent decrease in the levels of reporter gene expression, when compared to the wild type activator. These results indicate that the sequences mutated in constructs MUT 4 and MUT 5 are essential for the gene activation in the ring gland and that the sequences mutated in constructs MUT 1 and MUT 2 apparently promote correct levels of expression, without interfering with the developmental pattern of expression of the gene. The detection of expression only in embryos and Li in the MUT 3 series may be attributed to the fact the ring gland cells grow only by size, without proliferating, which could result in the dilution of the reporter protein in the ring gland cells of L3. A bioinformatics approach was undertaken in order to screen the *D. melanogaster* genome for sequences similar to the 67 bp (-2537/-187) enhancer. This analysis identified in the *D. melanogaster* genome 67 sequences 90–100 % identical to sequences contained in the ring gland enhancer, which are mainly located in *D. melanogaster* regulatory and intronic regions. It is possible that some of these *Drosophila* genes are under the same regulatory network as the *BhC4*-*1* gene and are also expressed in the ring gland. Consistent with the functional studies, the majority (96%) of the identified *D. melanogaster* sequences align to a 45 bp (-2317/-187) region, which comprises the regions mutated in constructs MUT3 (-2227-213), MUT4 (-210/-201) and MUT5 (-198–189), indicating that the distribution of the alignments throughout the ring gland enhancer is not random. Financial support FAPESP and PRP-USP.

# Research article: Role of host presence induced ecdysone in oogenesis of a synovigenic parasitoid wasp

**DOI:** 10.1673/031.007.1357

**Published:** 2007-03-08

**Authors:** N Mandon, F Vannier, A Bodin, JP Delbecque, B Jaloux, JP Monge, N Mondy

**Affiliations:** ^1^ Université François-Rabelais, IRBI UMR CNRS 6035, Parc Grandmont, 37200 Tours, France.; ^2^ Université de Bordeaux 1, LNR UMR CNRS 5816, Avenue des Facultés, 33405 Talence, France; * Current address: UMR CNRS 5023 Ecologie des hydrosystèmes fluviaux, Université Claude Bernard Lyon 1, 43 Bd du 11 Novembre 1918, 69622 Villeurbanne Cedex, France.

Insects have evolved a diversity of reproductive strategies in response to their environment. Parasitoids lay their eggs in or on the bodies of their hosts and feed off them as they develop. The so-called pro-ovigenic parasitoids are born with their entire complement of eggs. Synovigenic parasitoids, on the contrary, are born with a limited number of mature oocytes and are able to produce them during the entire female life in response to host availability. When environmental cues are not favourable to reproduction, females, by stopping their oogenesis, can increase their survival and in consequence increase the probability to find favorable conditions for laying eggs. Therefore reproduction success of a synovigenic parasitic wasp is directly related to an efficient stop-and-go mechanism of its oogenesis. The endocrine mechanisms underlying this system are poorly understood, especially for hymenopteran parasitoids. *Eupelmus vuilleti* (Hymenoptera; Eupelmidae) is a synovigenic solitary parasitoid producing yolk-rich eggs during its imaginai stage. The female oviposits on the third to fourth larval instars of *Callosobruchus maculatus* (Coleoptera; Bruchidae) which develop within pods and seeds of *Vigna unguiculata* (Fabacae). In a first step, the identity and titre of ecdysteroids in reproductively acive and inactive adult female parasitoids were investigated by EIA/HPLC. A large quantity of ecdysone was found in female wasps during their reproductive period compared with inactive females. In a second step, both the secretion of ecdysteroid into the medium of *in vitro* cultured ovaries and the edysteroid content of females reared with or without host was measured by EIA. The presence of the host, which represents both the oviposition site and the nutritional source for the female, induced an active biosynthesis of ecdysone. This synthesis started at a slow rate immediately after host introduction and reached a maximum after 48 hours. Moreover, if hosts were continually available, this synthesis was cyclic and continuous during the entire female lifetime. The involvement of ecdysone in the mechanisms allowing flexibility of oogenesis in synovigenic wasps and the nature of the ovarian steroidogenesis stimuli linked to host presence will be discussed.

# Research article: Functional analysis of the nuclear receptor E75 in the development of the hemimetabolous insect *Blattella germanica*

**DOI:** 10.1673/031.007.1358

**Published:** 2007-03-08

**Authors:** D Mané-Padrós, J Cruz, X Bellés, D Martín

**Affiliations:** Department of Physiology and Molecular Biodiversity, Institut de Biologia Molecular de Barcelona (CID, CSIC), Jordi Girona 18–26, 08034 Barcelona, Spain.

In insects, molting is controlled mainly by ecdysteroid hormones. The more active form of insect ecdysteroids, 20-hydroxyecdysone (20E), is bound by an heterodimer of the nuclear receptors EcR and USP. Downstream genes activated by EcR/USP include other nuclear receptors, clearly indicating that this family of transcription factors plays a crucial role in transducing the 20E signal. Relatively little is known about the ecdysteroid-mediated hierarchy of transcription factors in primitive insects. Here, we report on the cloning and characterization of five isoforms of E75, a member of the nuclear receptor family, in the hemimetabolous insect *Blattella germanica*. The five isoforms (BgE75-A, -B, -C, -D and -E) display specific 20E responsiveness and their mRNAs show clear differences in tissue- and stage-expression patterns. Moreover, we carried out RNAi for BgE75 in last instar nymph animals and observed efficient and reproducible silencing of gene function. Analysis of the knockdown phenotype revealed that BgE75 is essential for normal development of *B. germanica*. A detailed analysis of the BgE75 knock-down phenotype will be discussed.

# Research article: The heterodimer EcR/RXR is essential for germ band formation in the short-germ band insect *Blattella germanica*

**DOI:** 10.1673/031.007.1359

**Published:** 2007-03-08

**Authors:** D Martín, O Maestro, J Cruz, N Pascual, D Mané-Padrós, X Bellés

**Affiliations:** Department of Physiology and Molecular Biodiversity, Institut de Biologia Molecular de Barcelona (CID, CSIC), Jordi Girona 18–26, 08034 Barcelona, Spain.

In insects, major developmental transitions occurring throughout their life cycles are finely regulated by changes in the titre of the ecdysteroidal hormone, 20-hydroxyecdysone (20E). 20E induces these transitions upon binding to its heterodimeric receptor, which is formed by two members of the nuclear receptor superfamily, EcR and the RXR homolog USP. The activated receptor elicits cascades of gene expression that mediate and amplify the ecdysteroidal signal. Extensively analyses have been conducted using *Drosophila melanogaster* and *Manduca sexta* as insect models, which have clarified a number of functions regulated by these hormones during the post-embryonic development. However, the role played by ecdysteroids during the embryogenesis is poorly understood. In *D. melanogaster*, a model of long-germ band embryogenesis, there is only one mid-embryonic pulse of ecdysteroids. Moreover, ecdysteroid signalling is necessary for the relevant morphogenetic movements that shape the larvae, namely, germ band retraction and head involution. Data on the role played by ecdysteroids during the embryogenesis of the more primitive, short-germ band insects is even scarcer, mainly due to the fact that these species are not amenable to genetic analysis. The main goal of our research is to establish the role of ecdysteroids in the development of primitive insects, using the hemimentabolous species *Blattella germanica* as model. A detailed analysis of the ecdysteroid titer and the expression patterns of several genes belonging to the ecdysone-dependent genetic hierarchy throughout embryogenesis of *B. germanica* show that a recurring cascade of ecdysteroids-dependent gene expression occurs at 12%, 35% and 75% of development. Moreover, we have used a parental RNAi *in vivo* approach that allow us to efficient and specifically disrupt the embryonic gene function of the two components of the 20E receptor, EcR and RXR. A detailed analysis of the knockdown embryos shows that both nuclear receptors are required for the correct formation of the germ band. Taken together, our data indicates that in embryogenesis, ecdysteroids are required much earlier in primitive insects than in more derived ones.

# Research article: Characterization of a *Drosophila* prothoracicotropic hormone (PTTH) homologue

**DOI:** 10.1673/031.007.1360

**Published:** 2007-03-08

**Authors:** Z McBrayer, MJ O'Connor, JP Parvy, R Beckstead, JT Warren, C Dauphin- Villemant, C Thumell, LI Gilbert, MB O'Connor

**Affiliations:** ^1^ Dept of Genetics, Cell Biology, Howard Hughes Medical Institute and University of Minnesota, Minneapolis, MN; ^2^ Lab. Endocrinologie Moléculaire et Evolution, Université P. et M. Curie.; ^3^ Department of Genetics, University of Utah, Salt lake City Utah, carl.thummel@genetics.utah.edu; ^4^ Department of Biology, University of North Carolina.

Prothoracicotropic hormone (PTTH) is a neuropeptide hormone produced in the central nervous system of many insects. Release of this hormone from the brain is thought to regulate developmental timing by stimulating the prothoracic gland to synthesize and release ecdysone, a key hormone involved in growth, molting and adult metamorphosis. Although the PTTH amino acid sequence among different species of Lepidoptera (such as *Manduca sexta* and *Bombyx mori*) is fairly divergent, conserved patterns include (1) the number of cysteine residues, (2) the types of inter- and intramolecular bonds they make, and (3) the placement of amino acid types. This information was used to scan the *Drosophila melanogaster* genome, and a possible PTTH homologue (CG13687) was identified. Investigation of the mRNA profile by in-situ hybridization revealed very specific expression in two pairs of neurons in the brain lobes of 3rd instar larvae. In addition, experiments in transgenic fly lines using the UAS-Gal4 system and an HA-tagged “PTTH” revealed that these neurons innervate the prothoracic component of the ring gland in a pattern similar to the PG neurons identified by Siegmund and Korge (2001). Furthermore, targeted ablation of these neurons, using the UAS-Gal4 system to drive the apoptotic gene *grim*, resulted in considerable protraction of the 3rd larval instar. Affected animals did not achieve puparium formation until approximately 10 days after egg laying (5 days later than normal). This protracted 3rd instar caused prolonged feeding and growth, resulting in enlarged larva that formed enlarged pupae and enlarged adults. Animals that survived to eclosion were both viable and fertile. The prolonged larval life is the result of a delay in ecdysone production since it can be abrogated by feeding larvae 20-hydryoxecdysone. During the developmental delay, animals mount an appropriate, albeit belated and asynchronous transcriptional response that directs entry into metamorphosis. We hypothesis that in Drosophila, PTTH is not the primary output of the developmental timer, rather it affects the output levels of, or sensitivity of reception to, the timing signal.

# Research article: Size assessment and growth control: the role of the prothoracic gland and fat body in controlling adult size in *Drosophila*

**DOI:** 10.1673/031.007.1361

**Published:** 2007-03-08

**Authors:** C Mirth, JW Truman, LM Riddiford

**Affiliations:** Department of Biology, University of Washington, Seattle WA, USA.

Size control is a process common to all organisms. The mechanisms involved are two-fold: those that regulate growth rate, and those that assess when a sufficient size has been reached and growth should stop. We have shown that the growth of the prothoracic gland (PG) determines when critical weight has been reached (Mirth C et al. 2005. Curr. Biol. 15: 1796–1807). When PG growth was decreased by suppressing insulin signaling in the PG cells using the Gal 4 driver P0206, larvae delayed development and either metamorphosed as second instar pupae or as large third instar pupae (142% the weight of pharate adult controls). In contrast, enlarging the PG by hyper-activating insulin signaling in these cells with the Gal4 driver *phantom* caused formation of smaller puparia (73–80% of the controls). Rearing larvae under constant light enhanced these effects so that larvae reached critical weight 9 hours earlier and at much smaller sizes (41% of the control). We are currently testing whether DHR4, a repressor of ecdysone-responsive genes that is expressed solely in the PG around the time of critical weight, is involved in the PG-mediated size assessment event. Recently we have also found that reduction of fat body size throughout larval development mimicked the effects of starvation, and produced small, starvation-sensitive adults (83% of the controls) that initiated metamorphosis at normal times. Surprisingly, increasing fat body size after the mid-third instar also resulted in small adults, but these were starvation resistant. Therefore, early in larval life the fat body is likely controlling nutrition-dependent growth rates. Later, it may compete with other tissues for protein and lipid storage, leading to a reduction in size. Colombani J et al. 2005 (*Science* 310, 667–670) showed that eliminating the expression of EcR in the FB increases growth rates and pupal size. Therefore, we are testing whether the FB is a target tissue during PG-mediated size assessment. Supported by the Royalty Research Fund to LMR, Virginia and Prentice Bloedel University Professsorship to LMR and NIH R01NS29971 to JWT and LMR.

# Research article: Positive feedback regulation of prothoracicotropic hormone release by ecdysteroid

**DOI:** 10.1673/031.007.1362

**Published:** 2007-03-08

**Authors:** A Mizoguchi, M Kamimura, H Kataoka

**Affiliations:** ^1^ Division of Biological Science, Graduate school of Science, Nagoya University, Nagoya 464-8602, Japan.; ^2^ National Institute of Agrobiological Sciences, Tsukuba, Ibaraki 305-8634, Japan; ^3^ Department of Integrated Biosciences, Graduate School of Frontier Sciences, The University of Tokyo, Chiba 277-8562, Japan

Last instar larvae of lepidopterans stop feeding and start wandering several days after ecdysis, and these behavioral changes mark the initiation of the preparatory phase of larval-pupal metamorphosis. It is widely believed that this transition from feeding stage to metamorphic development is triggered by the prothoracicotropic hormone, PTTH, secreted by the brain-corpora cardiaca-corpora allata complex. Indeed, in *Bombyx mori*, the PTTH titer in hemolymph abruptly rises on day 5 or 6 of the fifth instar being accompanied by the increase in hemolymph ecdysteroid titer and the start of wandering. What regulates this surge release of PTTH, then? To address this issue, we focused on a small rise in hemolymph ecdysteroid titer that occurs the day before the PTTH surge, and conducted two lines of experiments to examine the role of ecdysteroid in the induction of the PTTH surge. First, a small amount of 20-hydroxyecdysone was injected two days before the expected day of PTTH surge to simulate the small increase in hemolymph ecdysteroid titer on the day before the PTTH surge. This injection led to a precocious surge of PTTH the next day, one day prior to the normal PTTH surge. Next, the hemolymph ecdysteroid titer on the day before the PTTH surge was artificially lowered by injecting ecdysteroid 22-oxidase, which inactivates 20-hydroxyecdysone. After this treatment, the PTTH surge was inhibited in 70% of the animals the next day, while the surge occurred in 90% of control animals. These results indicate that ecdysteroid is necessary to induce the PTTH surge. The mechanisms by which a small rise in hemolymph ecdysteroid titer is brought about and the biological meaning of the regulation of PTTH surge by ecdysteroid will be discussed.

# Research article: Selection and analysis of cell clones of Se4 resistant towards 20-hydroxyecdysone and methoxyfenozide

**DOI:** 10.1673/031.007.1363

**Published:** 2007-03-08

**Authors:** H Mosallanejad, T Soin, G Smagghe

**Affiliations:** Laboratory of Agrozoology, Department of Crop Protection, Faculty of Bioscience Engineering, Ghent University, Coupure links 653, B-9000 Ghent, Belgium.

Over the past few years, the beet armyworm, *Spodoptera exigua* (Hübner) (Lepidoptera: Noctuidae), has become a serious pest that has developed resistance to various groups of insecticides. Methoxyfenozide is one of the newest and the most effective of the non-steroidal ecdysone agonist insecticides that has been commercialized and used against Lepidoptera such as *Spodoptera* species. Cultured insect cells are known to be a valuable system for the study of the resistance process. In this study we obtained methoxyfenozide and 20-hydroxyecdysone (20E) resistant subclones of the Se4 cell line. This cell line originating from embryos of the beet armyworm, is sensitive for ecdysteroid activity stimulated by the insect molting hormone 20E and the dibenzoylhydrazine compounds. The objective of this study was to study possible mechanisms of resistance towards 20E and methoxyfenozide using Se4 cells. Gradually increasing concentrations of 20E and methoxyfenozide were added over a period of about one year. From these cultures, five methoxyfenozide resistant subclones and four 20E resistant ones were selected, showing resistance levels ranging between 100 and 3000 fold towards 20E and methoxyfenozide, respectively. They also displayed cross-resistance towards 20E and methoxyfenozide. It is concluded that this insect cell line has potential evolving high level of resistance to methoxyfenozide in a short time. The information we report can help to better explain the mechanisms behind the occurrence of insecticide resistance. Strategies to manage resistance to a particular insecticide have usually been devised after resistance has evolved. Therefore cell cultures can help to indicate resistance mechanisms towards a new insecticide before they evolve in the field, and so to proactively manage resistance processes in practice.

# Research article: Structure-activity relationship of non-steroidal ecdysone agonists and the prediction of the ligand binding to the ecdysone receptors of *Bombyx mori*

**DOI:** 10.1673/031.007.1364

**Published:** 2007-03-08

**Authors:** Y Nakagawa, CE Wheelock, T Harada, T Soin, G Smagghe, M Akamatsu, K Iatrou, L Swevers

**Affiliations:** ^1^ Graduate School of Agriculture, Kyoto University, Kyoto 606-8502, Japan.; ^2^ Faculty of Bioscience Engineering, Ghent University, Coupure links 653, B-9000 Ghent, Belgium; ^3^ National Center for Scientific Research “Demokritos”, Aghia Paraskevi, 153 10 Athens, Greece

Insect molting is regulated by the steroid hormone, 20-hydroxyecdysone (20E), and the disturbance of the ordinary hormone balance can lead to insect death. Since this hormonal regulation is unique to insects, ecdysone agonists can potentially be used as safe insecticides. To date, four diacylhydrazines (DAHs), tebufenozide, methoxyfenozide, chromafenozide, and halofenozide have been developed as insecticides. It is known that 20E and its agonists including DAHs bind to the ecdysone receptor (EcR). Billas and co-workers solved the three dimensional structures of ecdysone receptor proteins of lepidopteran *Heliothis virecens* with ponasterone A (PonA) and a DAH compound by X-ray crystal structure analysis and demonstrated that their ligand binding cavities are only partially overlapped. Two years later, the X-ray crystal structure of EcR of hemipteran *Bemisia tabaci* is published as PonA-bound form, but not for the DAH-bound form. We previously reported that the linear correlation between the larvicidal activity and the receptor binding in *in vitro* translated proteins was observed for *C. suppressalis*, but not for coleopteran *Leptinotarsa decemlineata*, by synthesizing DAHs with various substituents at benzene rings and measuring their their activity. In this study we constructed the ligand binding domain of the ecdysone receptor (EcR) of *Bombyx mori* from that of *H. virecens* EcR using a protein discovery full automated modelling system (PDFAMS). As a result, steric and hydrophobic interactions were visualized, and the possible hydrogen-bonds were predicted. We also measured the activity of 172 DAH compounds using a cell-based high throughput screening system in *Bombyx mori* cells, and quantitatively analyzed the structure-activity relationship using a 3D QSAR procedure, a comparative molecular field analysis (CoMFA), to derive the steric and electrostatic effects on the activity. Consequently, the proposed ligand-receptor binding model for EcR of *B. mori* is confirmed by CoMFA results. In the further study, we measured the larvicidal activity of DAHs against *B. mori* and compared with their activity in cells.

# Research article: Impact of heterodimerization and ligand on intracellular localization of the ecdysteroid receptor (EcR) receptor

**DOI:** 10.1673/031.007.1365

**Published:** 2007-03-08

**Authors:** C Nieva, M Spindler-Barth, K-D Spindler

**Affiliations:** Department of General Zoology and Endocrinology, University of Ulm, Germany.

Initial nuclear import of the ecdysteroid receptor EcR in vertebrate cells (CHO and COS-7) does not afford a heterodimerization partner. Later on EcR is retained only in the presence of a heterodimerization partner (Nieva C et al. 2005. Biol. Chem. 386: 463–470). Ultraspiracle (USP) is more efficient compared to its vertebrate orthologue RXR and leads to exclusively nuclear localization of EcR even in the absence of ligand. DNA binding conferred by the heterodimerization partner improves retainment of EcR in the nucleus as shown by Usp4 which has lost its DNA binding capability. The C-terminal end of USP (USPΔ205–508) encompassing the C-terminal part of the D-domain and the E- and F-domains of Ecr or Usp are (is) essential for retainment of EcR in the nucleus. In the absence of hormone the ecdysteroid receptor (EcR) is distributed between cytoplasm and nucleus. Addition of muristerone A increases nuclear localization of wild type EcR within 5–10 min. Mutation of M504 situated in helix 5 of the ligand binding domain, which is essential for ligand binding (Grebe et al, 2003. Biol. Chem., 34: 105–116) to alanine, still allows nuclear localization at subnormal levels. Cotransfection with ultraspiracle (USP), the invertebrate orthologue of RXR, leads to exclusively nuclear localization of wild type EcR and EcRM504Aindicating that heterodimerization in the absence of hormone is still possible. EMSA experiments show that the ligand muristerone A enhances binding of wild type EcR, but not of mutated EcRs, to the canonical hsp 27 ecdysone responsive element which is in agreement with transactivation studies. The results indicate that the architecture of the E-domain of EcR is important for nuclear localization even in the absence of a ligand and indicates that the conformation of the ligand binding pocket can be partially rescued by USP.

# Research article: Identification and characterization of the genes functioning in the prothoracic gland: A novel gene *neverland* is essential for ecdysone synthesis and insect growth

**DOI:** 10.1673/031.007.1366

**Published:** 2007-03-08

**Authors:** R Niwa, T Yoshiyama, T Namiki, K Mita, H Kataoka

**Affiliations:** ^1^ Department of Integrated Biosciences, Graduate School of Frontier Sciences, The University of Tokyo, Chiba, Japan.; ^2^ Laboratory of Insect Genome, National Institute of Agrobiological Sciences, Tsukuba, Ibaraki, Japan

During larval and pupal stages of insects, ecdysone is synthesized in the prothoracic gland (PG) and plays a central role in the control of development. Although many studies have revealed the biochemical features of ecdysone synthesis in the PG, many aspects of this pathway have remained unclear at the molecular level. Through a combined biochemical and molecular genetic approach using both the silkworm *Bombyx mori* and the fruit fly *Drosophila melanogaster*, we have identified and characterized genes responsible for ecdysteroidogenesis in the PG, including several cytochrome P450 genes (Niwa R et al. 2004. J. Biol. Chem. 279: 35942–35949; Niwa R et al. 2005. Insect Mol. Biol. 14: 563–571; Namiki T et al. 2005. *BBRC* 337:367–374). In this presentation, we report another novel gene called *neverland* (*nvd*), which encodes an oxygenase-like protein with a Rieske electron carrier domain, *nvd* is expressed specifically in tissues that synthesize ecdysone, such as the PG. We also show that loss of *nvd* function in the PG causes arrest of both molting and growth during *Drosophila* development. Furthermore, the phenotype is rescued by application of 20-hydroxyecdysone or the precursor 7-dehydrocholesterol. Given that the *nvd* family is evolutionally conserved, these results suggest that Nvd is a novel and essential regulator of cholesterol metabolism or trafficking during steroid synthesis across animal phyla. We will also discuss our data from functional analysis of the other PG-specific genes.

# Research article: Structure-activity relationship study of ecdysone agonists against Colorado potato beetle *Leptinotarsa decemlineata*

**DOI:** 10.1673/031.007.1367

**Published:** 2007-03-08

**Authors:** T Ogura, Y Nakagawa, C Minakuchi, G Smagghe, H Miyagawa

**Affiliations:** ^1^ Department of Agriculture, Kyoto University, Kyoto, Japan.; ^2^ Faculty of BioScience Engeneering, Ghent University, Ghent, Belgium

Ecdysone agonists have been expected to be safe insect control agents because the insect development system which is regulated by the molting hormone dose not exist in vertebrates such as human beings. There are various ecdysone agonists, and some of them have steroidal structures like the natural molting hormone 20-hydroxyecdysone (20E), but others have non-steroidal structures. The most famous non-steroidal ecdysone agonists are diacylhydrazines (DAH). Via interaction with the receptor of 20E, a heterodimer of the ecdysone receptor (EcR) and ultraspiracle (USP), DAH shows toxicity to insects in the species-selective manner. Especially, many of them are potent to Lepidoptera. Recently, we demonstrated that the binding affinity of DAH to the *in vitro* translated molting hormone receptor (EcR/USP) is correlated to their larvicidal activity against a lepidopteran insect, the rice stem borer *Chilo suppressalis*. Therefore, it is likely that the selective toxicity of DAH is caused by the difference of the receptor binding affinity among insects. On that account, it is necessary to compare the binding affinity of DAH to EcR/USP of Lepidoptera with insects in other orders. The purpose of our study is to elucidate the selective toxicity of DAH at the molecular level. Here, we report cDNA cloning of EcR and USP of the coleopteran Colorado potato beetle (*Leptinotarsa decemlineata*: LdEcR and LdUSP), and *in vitro* translation of these receptor proteins. After cDNA cloning using RT-PCR with degenerate primers and RACE method, we transcribed and translated cDNAs of LdEcR and LdUSP in a rabbit reticulocyte lysate system. SDS-PAGE of these in *vitro* translation products showed major protein products which have similar molecular weights with those calculated from amino acid sequences of LdEcR and LdUSP, respectively. Gel mobility shift assay using *Drosophila* hsp27 EcRE (ecdysone response element) revealed that in *vitro* translated LdEcR/LdUSP can bind to this EcRE sequence. These results suggested that the in *vitro* translation product is functional as a molting hormone receptor of *L. decemlineata*. As expected, the binding affinity of DAH to these products of *in vitro* translation was very different from that to the receptor of *C. suppressalis*. However, the binding affinity of DAH to LdEcR/LdUSP was not correlated to their larvicidal activity. Therefore, it is suggested that other factors are more important than the receptor binding affinity to the expression of the toxicity of DAH against *L. decemlineata*. A possible factor is the activation of transcription by DAH. To elucidate the effect of transactivation on the toxicity of DAH against *L. decemlineata*, we conducted the reporter gene assay using a *L. decemlineata* cell line. The activity of halofenozide was stronger than that of tebufenozide, although receptor binding affinities of them were almost the same.

# Research article: Two *spook* paralogous genes code for stage-specific components of the ecdysteroid biosynthetic pathway in *Drosophila melanogaster*

**DOI:** 10.1673/031.007.1368

**Published:** 2007-03-08

**Authors:** H Ono, KF Rewitz, JT Warren, LI Gilbert, MB O'Connor

**Affiliations:** ^1^ Department of Genetics, Cell Biology and Development, Howard Hughes Medical Institute, University of Minnesota, Minneapolis, MN, USA.; ^1^ Department of Biology, University of North Carolina, Chapel Hill, NC, USA.

20-hydroxyecdysone (20E), the molting hormone of insects, regulates transition through different developmental stages. Pulses of 20E are required for embryogenesis, larval molting and metamorphosis and 20E is also essential for proper oogenesis. Recently, the genes of the Halloween family that code for P450 enzymes which catalyze the final four steps have been identified in *Drosophila*. Mutations in these genes results in failure of embryonic development and exhibit common characteristic phenotypes such as dorsal closure, head involution and midgut morphogenesis. Of the Halloween P450S, only *spook* (*spo*) remains uncharacterized. We established novel rescue conditions for the Halloween lethal embryos by delivering an external pulse of an ecdysteroid to dechorionated embryos. *Spo* mutant embryos were rescued by 20E and some of them eclosed as adult. However, rescued *spo* mutant females were sterile and their ovaries failed to develop normally. Rescue experiments using intermediates in the ecdysteroid biosynthetic pathway indicate that Spo acts upstream of ketotriol. Spo is expressed in the yolk nuclei and amnioserosa during early embryogenesis and follicle cells in the ovary but is not expressed in the ring gland either during embryogenesis or in larval stages. The fact that Spo activity is not required during larval stages suggest that some other P450 may act in its place to provide ecdysteroid biosynthetic capabilities during post embryonic stage. We identified a spo homolog, dubbed *spookier* (*spok*), localized in heterochromatin. In contrast to *spo*, *spok* is specifically expressed in the ring gland after late embryogenesis. Expression of *spok* is eliminated in larvae carrying mutations in *molting defective* (*mld*), a gene encoding a nuclear zinc finger protein that is required for production of ecdysteroids during larval development. These results suggest that in *Drosophila* two *spook* genes divide their function at different developmental stages and Mld is a key component regulating *spok* expression timing. In contrast to *Drosophila*, we have identified only one spo homolog that is specifically expressed in ring gland in lepidopteran species. Interestingly, *mld* is not found in any species so far investigated other than Drosophilidae. Therefore, the presence of *mld* and closely related pairs of spo genes may indicate that these two events are functionally coupled. These studies suggest that an evolutionary split between Drosophilidae and Lepidoptera in regulation of the ecdysteroid biosynthetic pathway has occurred by gene duplication and divergence events.

# Research article: The role of ßFtz-f1 in *Drosophila* steroidogenesis

**DOI:** 10.1673/031.007.1369

**Published:** 2007-03-08

**Authors:** JP Parvy, E Pondeville, A Maria, C Dauphin-Villemant

**Affiliations:** Equipe Biogenèse des Stéroïdes, FRE2852 CNRS -Université P. et M. Curie, Paris, France.

In arthropods, ecdysteroids play crucial roles by initiating and regulating molting and metamorphosis. Identification of genes coding for cytochrome P450 enzymes involved in *Drosophila* ecdysteroidogenesis, i.e. *phantom* (*phm*), *disembodied* (dib), *shadow* (*sad*) and *shade* (*shd*) has provided new tools to investigate the regulation of insect hormone production. We have recently shown that there is a temporal correlation between dynamics of ecdysone production and expression of genes encoding steroidogenic enzymes, during the third instar, suggesting that the timing of hormone production depends on transcriptional regulation of the biosynthetic enzymes (Parvy JP et al.2005. Developmental Biology 282: 84–94). Using clonal analysis, levels of steroidogenic enzymes were shown to be very reduced in *ftz transcription factor 1* (*ftz-f1*) mutant ring gland cells, further suggesting that this transcription factor is involved in the regulation of *phm*, *dib* and sad expressions, at least in the third instar. In order to gain a better insight into the role of BFtz-f1 on ecdysteroid biosynthesis, interactions between its expression and steroidogenesis were studied at different stages of *Drosophila* development. Since BFtz-f1 is the homolog of the vertebrate steroidogenic factor 1 (SF1), which plays a key role in the differentiation of vertebrate steroidogenic organs through transcriptional regulation of steroidogenic enzymes, a better understanding of BFtz-f1 role in *Drosophila* should allow to precise the parallels between the regulatory mechanisms of steroidogenesis in insects and vertebrates.

# Research article: RNAi of the early gene *Broad* points to key functions during embryogenesis in a hemimetabolous insect

**DOI:** 10.1673/031.007.1370

**Published:** 2007-03-08

**Authors:** MD Piulachs, V Pagone, X Bellés

**Affiliations:** Department of Physiology and Molecular Biodiversity, Institut de Biologia Molecular de Barcelona (CID, CSIC), Jordi Girona 18–26, 08034 Barcelona, Spain.

Broad-Complex (BR-C) is a family of C2H2 type zinc-finger DNA-binding proteins involved in the genetic cascade triggered by 20-hydroxyecdysone. It is involved in metamorphosis, as shown in the fruit fly, *Drosophila melanogaster*, and in the moths *Manduca sexta* and *Bombyx mori*. More recently, BR-C has been shown to be involved in vitellogenesis and oogenesis in *D. melanogaster* and in the mosquito *Aedes aegypti*, both with vitellogenesis regulated by ecdysteroids. We have studied BR-C in the German cockroach, *Blattella germanica* (BgBR-C), a hemimetabolous insect with vitellogenesis regulated by juvenile hormone. By RT-PCR we cloned the cDNA for the full coding region of six isoforms of BR-C, namely Z1, Z2, Z3, and Z4 (which have homologous sequences in other insect species) and two new isoforms that we named Z5 and Z6 (the last one with homologous sequences in the genomes of *Apis mellifera* and *Tribolium castaneum*). BgBR-C organization shares a common amino-terminal BTB domain followed by a core region fused to one of the different C2H2 zinc fingers. In *B. germanica*, we studied the expression patterns of the six isoforms of BgBR-C in ovaries of adult females during the first gonadotrophic cycle as well as during embryogenesis. BR-C mRNA is always present in ovaries and embryos, showing variations often associated with hormonal patterns. RNAi was used to knockdown BgBR-C and to study the functions of BR-C related to reproduction. Double strand RNA was designed to target the core region, which is common to all BgBR-C isoforms (dsBgBR-C-com), and was expected to interfere all isoforms. dsBgBR-C-com was injected into the abdomen of newly emerged adult females, and reduction of BgBR-C mRNA levels was checked in the ovaries 1 and 6 days after the treatment. dsBgBR-C-com treated specimens showed reduced fecundity (20% of the experimental specimens did not form the ootheca). In addition, most of the embryos from those females that formed the ootheca showed morphological defects at different stages of development. The results point to key roles of BR-C in the embryogenesis of *B. germanica*.

# Research article: Evidence for a steroid-based sexual dimorphism in *Anopheles gambiae* adults

**DOI:** 10.1673/031.007.1371

**Published:** 2007-03-08

**Authors:** E Pondeville, A Maria, JC Jacques, C Bourgouin, C Dauphin-Villemant

**Affiliations:** ^1^ Equipe Biogenèse des Stéroïdes, FRE2852 CNRS - Université P. et M. Curie, Paris, France.; ^2^ Institut Pasteur, Paris, France

Ecdysteroids play essential roles by triggering molting and metamorphosis. Several studies have demonstrated that, at least in dipteran insects (mosquitoes and flies), these hormones also govern key events of reproduction (Raikhel AS et al. 2003. In: Henry HL, Norman AW, editors. Encyclopedia of Hormones, 1L 451–459. Academic Press; Swevers et al., 2005, In: Gilbert LI, Iatrou K, Gill SS, editors. Comprehensive Molecular Insect Science, 1: 87–155. Elsevier Pergamon). However, few data are available in the literature for *Anopheles gambiae* although this species is the vector of malaria, the most widespread of all tropical diseases and one of the most lethal (over 1 million deaths per year). We have studied steroidogenic function in adult female and male *A. gambiae*. The recent identification of genes encoding cytochrome P450 enzymes (CYPs) involved in *Drosophila* ecdysteroidogenesis has provided new molecular tools to investigate steroidogenic function in other species. We have established the functional conservation of these enzymes between *Drosophila* and *Anopheles*. Production and identity of gonadal steroids were determined using enzyme immunoassay and HPLC analyses and correlated to the tissue expression of steroidogenic enzymes in both males and females under different physiological conditions. Our results provide a precise basis for the characterization of adult steroidogenic tissues in relation to reproduction.

# Research article: The influence of ecdysteroid containing preparation serpisten on ionic currents of neurons

**DOI:** 10.1673/031.007.1372

**Published:** 2007-03-08

**Authors:** VI Prosheva, AI Vislobokov, VV Volodin

**Affiliations:** ^1^ Institute of Biology, Russian Academy of Sciences, 28 Kommunisticheskaya Street, Syktyvkar, 167982, Russian Federation.; ^2^ I.P. Pavlov State Medical University in Saint-Petersburg, 6/8, Leo. Tolstoy Street, Saint-Petersburg, 197022, Russian Federation

We have investigated the effects of preparation Serpisten, containing natural ecdysteroids mixture from the plant *Serratula coronata* L. (Asteraceae) on potassium, calcium and sodium ionic currents in isolated neurons of the snail *Lymnaea stagnalis*. The voltage-clamp technique was used. Serpisten was applied in 0.01–1000 µg/ml range concentration. It was shown that Serpisten activates non-selectively potassium and calcium currents in all range of studied concentrations (it increases currents amplitude by 2–15 %) and reduces nonspecific membrane leakage currents. The sodium currents at Serpisten action in low concentrations (0.01–10 µg/ml) were also increased in comparison with control but they were reduced by 5–10 *%* at concentrations of 100–1000 µg/ml. The effects were reversible. The kinetics of currents under the influence of Serpisten was not changed. This research was supported by grant from the Program of Presidium of RAS “Fundamental sciences for medicine”.

# Research article: Involvement of the receptor for activated C kinase 1 (RACK1) in the expression of the transcription factor CHR3 induced by 20-hydroxyecdysone in the spruce budworm, *Choristoneurajumiferana*

**DOI:** 10.1673/031.007.1373

**Published:** 2007-03-08

**Authors:** G Quan, D Doucet, PJ Krell, BM Arif, Q Feng

**Affiliations:** ^1^ Great Lakes Forestry Centre, 1219 Queen Street East, Sault Ste. Marie, Ontario, Canada, P6A 2E5.; ^2^ Department of Molecular and Cell Biology, University of Guelph, Ontario, Canada, N1G 2W1.

The ecdysone signal is transducted by nuclear receptors and the ligand-receptor complex to mediate changes in gene expression. Most steroid hormone receptors, such as EcR and USP are phosphorylated. Phosphorylation or dephosphorylation finely regulates receptor functions such as hormone binding, DNA binding, translocation and recognition of the hormone response element. Phosphorylation of the ecdysone receptor components are regulated by 20-hydroxyecdysone (20E). However, it remains unknown which kinase signal transduction components are involved in the 20E-regulated phosphorylation of the transcription factors. We demonstrate here that receptor of activated C kinase 1 (RACK1)/protein kinase C (PKC) are involved in the 20E-induced expression of the molt-associated transcription factor CHR3. A cDNA clone encoding the receptor for activated C kinase 1 was isolated from *Chonstoneura fumiferana* (C*f*RACK1). C*f*RACK1 protein contained seven repeating elements and is a homologous to the guanine nucleotide-binding protein beta subunit (GP-β). Expression of C*f*RACK1 occurred in most insect tissues and developmental stages as tested. High levels of transcripts were also detected in a midgut-derived CF-203 cell line. A GFP-fused C*f*RACK1 protein was found to distribute in the cytosol surrounding the nuclei in stably transformed cells. Double-stranded RNA mediated interference (RNAi) of C*f*RACK1 or inhibition of its binding to PKC with Dequalinium-14; 1,1′ - Decamethylenebis-4-aminoquinaldinium diiodide (DECA), suppressed the induction of CHRs expression by 20E. DEAC also blocked the accumulation of the EcR in the nuclei. These data imply that the RACK1/PKC signal transduction cascade is involved in phosphorylation of the ECR, which may facilitate the import of the receptor into the nuclei of cells.

# Research article: Ecdysteroid gene regulatory hierarchy during mosquito reproduction

**DOI:** 10.1673/031.007.1374

**Published:** 2007-03-08

**Authors:** AS Raikhel, J Zhu

**Affiliations:** Department of Entomology, University of California, Riverside, CA.

In mosquitoes, the steroid hormone 20-hydroxyecdysone (20E) functions as a generalized systemic signal coordinating critical events of reproduction. Newly eclosed female mosquitoes are not competent to respond to this hormone. The βFtz-F1 orphan nuclear receptor acts as a competence factor for the 20E response. It recruits a p160/SRC coactivator of the ecdysone receptor, FISC, to the functional ecdysone receptor in a 20E-dependent manner, leading to enhanced local histone H4 acetylation and robust activation of target genes. The major yolk protein precursor gene *vitellogenin* (*Vg*), expressed in the mosquito fat body, is a 20E effector gene that is dually controlled by the ecdysteroid receptor complex (EcR/USP) and by the products of ecdysteroid early responsive genes (*E74*, *E75*, and *Broad*). RNA interference analyses showed that E74B and Broad Z2 are activators of *Vg* expression, while Br Z1 and Z4 are repressers. Z4 serves as a “timer” coordinating the decline in *Vg* expression. E74B and the ecdysteroid receptor synergistically enhanced 20E-induced transcription of the *Vg* promoter. This action requires E74 binding sites and ecdysone response elements in the *Vg* 5′ regulatory region. Two-hybrid assays and co-immunoprecipitation analyses demonstrated direct interaction between E74B and EcR/USP. Moreover, disruption of this interaction by a dominant negative E74 mutant abolished the enhanced activation of *Vg*. Therefore the cooperative interaction between E74B and the ecdysteroid receptor is required for high-level expression of the *Vg* gene *in vivo*. This is accomplished through 20E-dependent protein-protein interaction on the *Vg* promoter. This study reveals how the direct-indirect regulation of a 20E effector gene is achieved at the molecular level.

# Research article: Diversity of detoxification pathways of ingested ecdysteroids among phytophagous insects

**DOI:** 10.1673/031.007.1375

**Published:** 2007-03-08

**Authors:** K Rharrabe, S Alia, A Maria, F Sayah, R Lafont

**Affiliations:** ^1^ Faculté des Sciences et Techniques, Laboratoire de Biologie Appliquée, P.O Box 416, Tangier, Morocco.; ^2^ Université Pierre et Marie Curie, Laboratoire Protéines: Biochimie Structurale et Fonctionnelle, CNRS FRE 2852, 7 Quai St. Bernard, 75252 Paris 05, France; ^3^ INRA, Laboratoire Physiologie de l'Insecte, Signalisation et Communication, UMR 1272, Route de St Cyr, 78026 Versailles Cedex, France

Detoxification mechanisms of ingested ecdysteroids in insects classically involve either conjugation of secondary alcohol functions (2-, 3-, or/and 22-OH) or epimerization of the 3-OH (Lafont R et al. Rees HH. 2005. In: Gilbert LI, Iatrou K. Gill S, editors, Comprehensive Molecular Insect Science, Elsevier, Vol. 3: 125–196). The already described conjugation reactions include the formation of polar 2/3-phosphates (e.g. in locusts) and possibly sulfates (some Diptera), or of apolar 3-acetates (*Locusta*, *Schistocerca*) or 22-acyl esters (e.g. various larvae of Lepidoptera-*Heliothis*, *Spodoptera* -, house crickets-*Gryllus*). 3-Epiecdysteroids have essentially been described in Lepidoptera (*Manduca*, *Pieris*). The present work has investigated the metabolic pathways of ingested 20-hydroxyecdysone in several insect species. We will describe here data obtained with the aphid *Myzus persicae* and the lepidopteran *Plodia interpunctella*. The former produces mainly a 22-glucoside conjugate, whereas the latter eliminates a mixture of 20E and its 3-oxo-derivative, both in free form and as conjugates with various fatty acids. Although ecdysteroid glucoside formation in insects was early described in *Calliphora* (Heinrich G, Hoffmeister H. 1970. Z. Naturforsch B 25: 358–361), glycosides were then only isolated from *Manduca sexta* eggs (Thompson MJ et al. 1987. Arch. Insect Biochem. Physiol. 4: 1–15), or as the products of an inactivation reaction performed by baculoviruses (O'Reilly DR et al. 1987. Insect Biochem. 21: 795–801). The simultaneous formation of 3-oxo ecdysteroids and of acyl esters has not been reported before. These data point out the great diversity of detoxification mechanisms used by phytophagous insects in order to overcome the potential harmful effects of ecdysteroids present in their food.

# Research article: The effects of 20-hydroxyecdysone on larval development and ultrastructure of the midgut epithelial cells of *Plodia interpunctella*

**DOI:** 10.1673/031.007.1376

**Published:** 2007-03-08

**Authors:** K Rharrabe, N Ghailani, F Sayah

**Affiliations:** ^1^ Faculté des Sciences et Techniques, CEEM. Laboratoire de Biologie Appliquée & Sciences de l'Environnement, BP. 416, Tanger, Maroc.; ^2^ Université Pierre et Marie Curie, Laboratoire Protéines: Biochimie Structurale et Fonctionnelle, CNRS FRE 2852, 7 Quai St. Bernard, 75252 Paris 05, France.

Exogenous administration of 20-hydroxyecdysone (20E) to some insect species has been reported to induce marked developmental disruption (Kubo I et al. 1981. Agric. Biol. Chem. 45: 1925–1927; Tanaka Y, Takeda S. 1993. J. Insect Physiol. 39: 805–809). Certain other insects, however, are able to tolerate 20-hydroxyecdysone or ecdysone without any adverse effects on growth and development (Blackford M et al. 1996. J. Insect Physiol. 42: 931–936; Blackford M, Dinan L. 1997. Insect Biochem. Molec. Biol. 27:167–177). The aim of this study was to determine the effects of ingested 20-hydroxyecdysone on the fourth instar larvae of the phytophagous pest *Plodia interpunctella* (Lepidoptera, Pyralidae). When incorporated into the diet at different doses, 20E disrupts insect development by inducing precocious pupation and adult emergence in a dependent manner. In addition, it caused cannibalism between larvae, weight loss and a significant mortality of larvae. 20E provoked also a reduction in protein and glycogen contents and a decrease of α-amylase activity. Using electron microscopy, we showed that 20E induced a severe cytotoxicity on the epithelial cells of the midgut. Cells exhibited marked vacuolization of the cytoplasm that enclosed numerous multivesicular bodies corresponding to typical autophagic vesicles characterized by their content of more or less degraded organelles. Other prominent features were the occurrence of pycnotic nuclei and fragmented rough endoplasmic reticulum cisternae, the appearance of spherocrystal structures with concentric layers, the disruption of microvilli and the rupture of the plasma membrane leading to the shedding of cytoplasm contents into the gut lumen. These described ultrastructural features triggered by 20-hydroxyedysone in midgut epithelial cells of *Plodia interpunctella* larvae are similar to those observed in apoptotic processes in programmed cell death or at onset of metamorphosis (Komuves L et al. 1985. Cell Tissue Res. 240: 215–221; Lee C.Y et al. 2002. Dev. Biol. 205: 101–111).

# Research article: Ligand binding and ligand induced changes of edysone receptor isoforms

**DOI:** 10.1673/031.007.1377

**Published:** 2007-03-08

**Authors:** H Ruff, A Azoitei, Braun, J Beatty, VC Henrich, M Spindler-Barth

**Affiliations:** ^1^ Department of General Zoology and Endocrinology, University of Ulm, Germany; ^2^ Institute for Health, Science, and Society; 209 Forney Buildiung, University of North Carolina Greensboro, Greensboro, NC 27402-6170 USA.

The minimal amino acid sequence capable of ligand binding encompasses the C- terminal part of the D-domain and the E-domain of *Drosophila* ecdysteroid receptor (EcR) (Grebe M et al. 2004. Biol. Chem. 384: 105–116). The influence of intracellular interactions between receptor domains of EcR and the intermolecular influence of Ultraspiracle (Usp) domains on ligand binding were examined. The interaction with ecre's was studied by EMSA. The results show that DNA binding varies between EcR isoforms and is modified in a complex manner by heterodimerization and hormone treatment. Transactivation potency varies not in parallel to DNA binding but seems to be regulated separately. The comparison of receptor isoform activity requires careful determination of the receptor concentration. Different methods of normalization on receptor content are compared. The influence of comodulators and cell cycle on receptor activity is discussed.

# Research article: Molecular mechanisms of the cellular control by ecdysteroids and JH in a *Plodia interpunctella* cell line

**DOI:** 10.1673/031.007.1378

**Published:** 2007-03-08

**Authors:** D Siaussat, F Bozzolan, P Porcheron, S Debernard

**Affiliations:** UMR 1272 Insect Physiology: Signaling and Communication, Pierre and Marie Curie University, Paris, FRANCE.

The process of growth and cellular differentiation are under control of 20-hydroxyecdysone (20E) and juvenile hormone (JH) during the post-embryonic development of the Lepidopteran. The 20E induces the processes of molt whereas JH conducted, by its presence, to a larval molt and, by its absence, to a nymphal or adult molt. Results put in evidence a control of the cell cycle by these hormones. At the beginning of the last larval instar, the imaginai wing disks are less sensitive to JH and accelerate their growth under the 20E control. At the end of stage, the increase in the 20E hemolymphatics concentrations entails an arrest of proliferation and cellular differentiation preceding the process of metamorphosis. The IAL-PID2 cell line of the imaginal wing disks of the last larval instar of *Plodia interpunctella* identicaly respond to a 20E physiological treatment by a blocking in G2/M. We undertook to identify the molecular mechanisms involved in the reception and integration of the hormonal signal leading to this proliferative arrest then cellular differentiation, and the possible modulations of these events by the JH. The temporal organization of the molecular actors of the 20E cascade begins with the induction of PiEcR-B1 mRNA (20E receptor). Hormonal response is followed by a high induction of the early gene, *PiE75*-*B,* then of early-late gene, *PHR3*. A 20E treatment of the cells during a window of sensitivity located in the S/G2 transition entails a decrease of expression of *Plodia* cycline B implicated in the mitosis control, and the arrest of the cells in G2/M. The inhibition of the PiEcR-B1 mRNA by the RNA interference method disrupts the 20E's cascade, allows the maintenance of expression of the cyclin B and prevents the proliferative arrest. In this model, the JH inhibits the anti-proliferative effects of 20E and would be susceptible to maintain the growth of imaginal cells.

# Research article: The effect of the anti-ecdysteroidal fungicide fenarimol on the crustacean *Daphnia magna* using a molting, life-stage and energy related microarray

**DOI:** 10.1673/031.007.1379

**Published:** 2007-03-08

**Authors:** A. Soetaert, T. Vandenbrouck, P. van Remortel, W.M. De Coen

**Affiliations:** ^1^ Laboratory for Ecophysiology, Biochemistry and Toxicology, Department of Biology, University of Antwerp, Belgium.; ^2^ Intelligent System Lab, Department of Mathematics and Informatics, University of Antwerp, Belgium.

In the present study, a molting related cDNA library for the crustacean *Daphnia magna* was constructed. As there is still little genome information on *Daphnia magna* available, the suppression subtractive hybridization (SSH) method was used to isolate molting related genes. This PCR based method allows the selective amplification of genes differentially expressed between two RNA populations. Daphnids were exposed to the molting hormone 20-hydroxyecdysone and pooled RNA from, respectively, the exposed versus control juveniles was used to perform an SSH. A total of 800 clones were picked and a selection of 188 fragments was sequenced and subjected to blast homology. Fragments belonging to different pathways such as cuticle-chitin metabolism, proteolytic enzymes and development, were present. Moreover, at the organismal level molting of the exposed daphnids was significant different from the controls after 24h which was comparable with the effect on molting found by Bodar (1990) at the same concentrations. The molting related genes -together with life-stage and energy metabolism related genes - were applied on a glass slide for the construction of a *Daphnia magna* microarray. In order to evaluate the usefulness of this custom microarray for toxicant characterization, gene expression profiles were generated from neonates exposed during 48h and 96h to three concentrations of the anti-ecdysteroidal fungicide fenarimol (0.5, 0.75,1 µg/ml). In total, 59 non-redundant genes were differentially expressed, of which more genes were down- than up-regulated. The gene expression data indicated mainly an effect on molting specific pathways. A set of proteolytic enzymes - including different serine proteases and carboxypeptidases - were induced whereas different cuticula proteins were down-regulated (48h 1µg/ml). Moreover, embryo developmental effects were not only demonstrated on the gene expression level but also on the organismal level. Two embryo development related genes, i.e. vitellogenin and homeobox (Xvent-1), were differentially expressed after 96h of exposure followed by a significant increase in embryo abnormalities in the offspring. These results suggest that this *Daphnia magna* microarray is of great further value for the elucidation of molecular mechanisms of toxicity and for the future development of specific biomarkers for hazard characterization. This project was funded by the IWT and the EU-project NoMiracle.

# Research article: The effect of three commercial ecdysone agonists on the moulting of the mysid shrimp, *Neomysis integer*

**DOI:** 10.1673/031.007.1380

**Published:** 2007-03-08

**Authors:** T Soin, A Ghekiere, CR Janssen, T Verslycke, G Smagghe

**Affiliations:** ^1^ Department of Crop Protection, Faculty of Bioscience Engineering, Ghent University, Ghent, Belgium.; ^2^ Laboratrory for Environmental Toxicology, Department of Applied Ecology and Environmental Biology, Faculty of Bioscience Engineering, Ghent University, Ghent, Belgium.; ^3^ Woods Hole Oceanographic Institution, MA, USA. Correspondencetim@whoi.edu

During the past decade, a large number of studies have reported interactions of some environmental contaminants with the development and functioning of endocrine systems in animals and humans. Invertebrates account for roughly 95% of all animals, yet in comparison with vertebrates, very little is known about their endocrinology and the possible effects of these endocrine disrupting substances. Mysid shrimps may serve as a viable surrogate for many crustaceans and have been proposed as suitable test organisms for evaluation of endocrine disruption by several researchers and regulatory bodies (e.g., the U.S. Environmental Protection Agency). Despite the longstanding use of mysids in ecotoxicity testing, little information exists on their endocrinology. In this project, the acute toxicity and sublethal effects of three non-steroidal ecdysone agonists in the estuarine mysid *Neomysis integer* were investigated. The tested ecdysone agonists are commercially used to control pest Lepidoptera in agriculture, horticulture and forests.

# Research article: Purification, kinetic characterization, and molecular cloning of a novel enzyme, ecdysteroid 22-kinase

**DOI:** 10.1673/031.007.1381

**Published:** 2007-03-08

**Authors:** H Sonobe, T Ohira, K Ieki, M Ajimura, K Mita, MN Wilder

**Affiliations:** ^1^ Department of Biology, Konan University, Kobe, Japan.; ^2^ Japan International Research Center for Agricultural Sciences, Tsukuba, Japan; ^3^ Genome Research Department, National Institute of Agrobiological Sciences, Tsukuba, Japan.

Although glucosides, fatty acyl esters and sulfate esters of ecdysteroids are detected as minor products of phase II reaction in several insect species, the major products of the phase II reaction in most insect species are phosphate esters. The ovaries of most insect species have the capacity to accumulate ecdysteroid phosphates, which are physiologically inactive (Makka T et al. 2002. Arch. Insect Biochem. Physiol. 51:111–120), in addition to the capacity to synthesize free ecdysteroids *de novo*. It has been suggested that the ecdystreoid phosphates that are accumulated in the ovaries are transferred to the eggs, and function as a source of free active ecdysteroid before the prothoracic glands differentiate during embryonic development. Recently, in *B. mori* eggs, a novel enzyme ecdysteroid-phosphate phosphatase (EPPase), which is specifically involved in dephosphorylation of ecdysteroid phosphates, was isolated, characterized and revealed to be a vital enzyme that may control the “on/off switch” of embryonic development (Yamada R, Sonobe H. 2003. J. Biol. Chem. 278: 26365–26373). In this study, ecdysteroid 22-kinase (EcKinase) was purified from the cytosol of the silkworm *Bombyx mori* ovaries to about1,900-fold homogeneity in six steps of column chromatography, and the biochemically characterized. Results obtained indicated that the reciprocal conversion of free ecdysteroids and ecdysteroid 22-phosphates by two enzymes, EcKinase and EPPase, plays an important role in ecdysteroid economy of the ovary-egg system of *B. mori*. On the basis of the partial amino acid sequence obtained from purified EcKinase, the nucleotide sequence of the cDNA encoding EcKinase was determined from an expression sequence tag (EST) clone prepared from *B. mori* eggs. The full-length cDNA of EcKinase was composed of 1,850 bp with an open reading frame encoding a protein of 386 amino acid residues. A database search showed that EcKinase has an amino acid sequence characteristic of phosphotransferases such as harboring Brenner's motif and putative ATP binding sites, but there are no functional proteins that share high identity with the amino acid sequence of EcKinase.

# Research article: EcR gene switches for regulated expression of transgenes in plants

**DOI:** 10.1673/031.007.1382

**Published:** 2007-03-08

**Authors:** VS Tavva, SR Palli, RD Dinkins, GB Collins

**Affiliations:** ^1^ Plant and Soil Sciences Department,; ^2^ Department of Entomology, University of Kentucky, Lexington, KY USA; ^3^ USDA-ARS-FAPRU, Lexington, KY, USA.

Genetic engineering of plants through transgene technology is being used to enhance agronomic performance or improve quality traits in a wide variety of plant species, and has become a fundamental tool for basic research in plant biotechnology. Constitutive promoters are presently the primary means used to express transgenes in plants. Metabolic energy waste, negative pleiotropic effects and potential gene escape are some of the disadvantages associated with the use of constitutive promoters. Inducible gene regulation systems based on specific chemicals have many potential applications in agriculture and in the basic understanding of gene function. As a result several gene switches have been developed. However, the properties of the chemicals used in these switches make their use limited to research purposes. An ecdysone receptor gene switch is one of the best inducible gene regulation systems available, because the chemical, methoxyfenozide required for its regulation is registered for field use. An EcR gene switch with a potential for use in large-scale field applications and its applicability to a variety of plant species has been developed by adopting a two-hybrid format. In a two-hybrid switch format, the GAL4 DNA binding domain (GAL4 DBD) was fused to the ligand binding domain (LBD) of the *Choristoneura fumiferana* ecdysone receptor (CfEcR); and, the VP16 activation domain (VP16 AD) was fused to LBD of *Locust migratoria* retinoid x receptor (LmRXR) or *Homo sapiens* retinoid x receptor (HsRXR). Upon application of methoxyfenozide, the heterodimer of these two fusion proteins transactivates the luciferase reporter gene placed under the control of multiple copies of cis acting elements and a minimal 35S promoter. The sensitivity of the CfEcR gene switch was improved from micromolar to nanomolar concentrations of ligand by using the CfEcR:LmRXR two-hybrid combination and a reduction in the background expression levels was achieved by using the CfEcR:HsRXR two-hybrid combination. The performance of EcR gene switch was improved further using Hs-LmRXR chimeras and/or CfEcR mutants. The efficiency of EcR gene switches in inducing the target gene expression was also tested in functional genomic studies by regulating the expression of a Superman-like single zinc finger protein 11 (ZFP11) gene in both Arabidopsis and tobacco plants. In addition, determination of pleiotropic effects of switch components and ligands is also a prerequisite for wide-spread use of these gene regulation systems in research and field applications. Therefore, we have also carried out the microarray analysis of the gene switch Arabidopsis plants to determine if there are pleiotropic effects caused by the introduction of a methoxyfenozide-inducible ecdysone receptor-based gene regulation system. The development of a highly sensitive and tightly regulated EcR gene switch along with other desirable properties such as availability of safe and field registered ligand should provide widespread use for this system.

# Research article: Effects of ingested phytoecdysteroids on the caterpillars of different polyphagous insects

**DOI:** 10.1673/031.007.1383

**Published:** 2007-03-08

**Authors:** K Ufimtsev, T Shirshova, V Volodin

**Affiliations:** Institute of Biology, Russian Academy of Sciences, 28 Kommunisticheskaya Str., Syktyvkar, 167982, Russian Federation.

The influence of the ecdysteroid-containing diet (the leaves of the plant *Serratula coronata* L., Asteraceae; natural ecdysteroids mixture isolated from this plant; individual ecdysteroids: 20-hydroxyecdysone, 25S-inokosterone and ecdysone) on the behaviour and the development of the caterpillars of three species of lepidopterous: *Mamestra brassicae* L. (Lepidoptera: Noctuidae), *Ostrinia nubilalis* Hb. (Lepidoptera: Pyralidae) and *Spodoptera littoralis* Boisd. (Lepidoptera: Noctuidae) and also on the fecundity of imago is studied. It is shown that introduction of leaves of the plant *Serratula coronata* or phytoecdysteroids into the artificial media leads to the mass migration of the first instar larvae of *Mamestra brassicae* and *Ostrinia nubilalis* and to their death. In comparison with the first instar larvae the third and 4^th^ instar larvae of *Mamestra brassicae* at first intensively fed on the media with different ecdysteroids content however after that they had rejected food. It results in outburst of cannibalism and total death of the caterpillars. Caterpillars remained feeding on ecdysteroid containing diet had formed abnormal pupa. Only solitary emergence of imago was observed. Caterpillars of *Spodoptera littoralis* are thought to be tolerant towards high ecdysteroids concentration. We established the fact that phytoecdysteroids indeed do not have antifedant effect but they caused significant reducing the numbers of eggs in egg mass. We are grateful to Prof. Frantisek Sehnal for the possibility to conduct the experiments with *Spodoptera littoralis* in his laboratory. This research was supported by a grant from the Program of collaboration between Ural and Siberian Divisions of Russian Academy of Sciences, Project N 151.

# Research article: Inhibitory effect of the ecdysteroid signaling by the juvenile hormone analogues, pyriproxyfen, kinoprene and fenoxycarb, in a dipteran (S2) and lepidopteran (Bm5) cell line

**DOI:** 10.1673/031.007.1384

**Published:** 2007-03-08

**Authors:** K. Van Loocke, R. Efrose, V. Labropoulou, L. Swevers, K. Iatrou, G. Smagghe

**Affiliations:** ^1^ Laboratory of Agrozoology, Faculty of Bioscience Engineering, Ghent University, Coupure Links 653, 9000 Ghent, Belgium.; ^2^ Institute of Biology, National Centre for Scientific Research “Demokritos,” P. O. Box 60228, Aghia Paraskevi Attikis, 153 10 Athens, Greece

At the molecular level, the activity of the juvenile hormone (JH) and its analogues (JHA) is not well understood. To analyze the possibility that JHIII and JHA can interact with the ecdysone receptor (EcR), a cell-based reporter assay was developed containing an ecdysone-dependent reporter construct in dipteran (S2) and lepidopteran (Bm5) cells, respectively. Tests to analyze the responsiveness of the reporter construct by JHIII and JHA showed no agonistic activity. On the contrary, the responsiveness of the reporter construct by 20E (500 nM) was significantly reduced in addition of the JHAs pyriproxifen, kinoprene and fenoxycarb. In Bm5 cells, fenoxycarb, kinoprene and pyriproxifen reached IC50 values of 1.0 10^-5^, 1.3 10^-5^and 3.5 10^-5^M, respectively, whereas the IC50 values of the JHA in S2 cells amounted 3.0 10^-7^, 5.2 10^-6^and 2.0 10^-6^M, respectively. Additionally, both cell lines were tested on cell toxicity caused by the JHA. Bm5 cells showed no cell toxicity in addition of kinoprene whereas fenoxycarb and pyriproxifen were toxic when adding 10^-4^-10^-7^and 10^-4^M, respectively. In S2 cells, only 10^-4^M of the JHAs caused cell toxicity. These results suggest that JHA in both Bm5 and S2 cells can interfere in the ecdysteroid response. Taking into account both responsiveness and cell toxicity, kinoprene followed by pyriproxifen showed the highest inhibitory effect in the ecdysteroid signal of Bm5 cells. Fenoxycarb showed no specific ecdysteroid inhibitory activity in this cell line owing to its general toxicity. Concerning S2 cells, fenoxycarb, kinoprene and pyriproxyfen showed in order of appearance the largest interference in the ecdysteroid signaling pathway.

# Research article: Saponins inhibit cotton leafworm, *Spodoptera littoralis*, development, possibly by reducing ecdysteroid receptor responsiveness

**DOI:** 10.1673/031.007.1385

**Published:** 2007-03-08

**Authors:** K Van Loocke, D Geelen, L Swevers, K Iatrou, G Smagghe

**Affiliations:** ^1^ Laboratory of Agrozoology and; ^2^ Department of Plant Production, Faculty of Bioscience Engineering, Ghent University, Coupure Links 653, 9000 Ghent, Belgium.; ^3^ Institute of Biology, National Centre for Scientific Research “Demokritos,” P. O. Box 60228, Aghia Paraskevi Attikis, 153 10 Athens, Greece.

Saponins are a group of steroidal or triterpenoidal secondary plant metabolites that have divergent biological activities. Because they are structurally reminiscent to steroids that activate the molting process in insects, we investigated saponin toxicity against insects. Here we analysed the impact of saponin on the growth of larvae of the cotton leafworm, *Spodoptera littoralis*. After 9 days *in vivo* feeding, larvae showed 57 to 70% mortality at doses ranging from 3 to 5.75%. A dose-dependent developmental arrest of third instar larvae of *S. littoralis* were also observed, pointing to the possibility that saponins interfere with molting. Therefore, we investigated the responsiveness of the molting receptor using the ecdysteroid receptor (EcR) reporter system in *Drosophila* S2 and *Bombyx* Bm5 cells. Application of saponins to these cells, significantly reduced the inducible expression of the EcR reporter. 20-hydroxyecdysone (20E) and ponasteroneA (ponA) mediated induction were both equally reduced by saponins. A similar inhibitory action was scored against the non-steroidal ecdysteroid agonist, tebufenozide. The *in vitro* assays suggest that saponin toxicity toward *Spodoptera* larvae is mediated by competitive inhibition of ecdysteroid signaling.

# Research article: Structure-Activity Relationship of brassinosteroids (BRs) and BR-hybrids in ecdysteroid signaling in a lepidopteran (Bm5) and dipteran (S2) cell line

**DOI:** 10.1673/031.007.1386

**Published:** 2007-03-08

**Authors:** K Van Loocke, Y Nakagawa, B Watanabe, L Swevers, K Iatrou, D Geelen, D Reheul, G Smagghe

**Affiliations:** ^1^ Laboratory of Agrozoology,; ^2^ Department of Plant Production, Faculty of Bioscience Engineering, Ghent University, Coupure Links 653, 9000 Ghent, Belgium.; ^3^ Division of Applied Life Sciences, Kyoto University; Kyoto 606-8502, Japan.; ^4^ Institute of Biology, National Centre for Scientific Research “Demokritos,” P. O. Box 60228, Aghia Paraskevi Attikis, 153 10 Athens, Greece

Brassinosteroids (BRs) are a group of steroidal plant hormones showing large similarities with the insect molting hormone, 20-hydroxyecdysone (20E), and its close analog, ponasterone A (ponA). Recent findings suggest that BRs and their hybrids can influence the insect molting process by interfering in the ecdysteroid signaling. To analyze these implications, BRs and BR-hybrids were tested on transfected cells of the dipteran, *Drosophila melanogaster*, (Schneider 2 or S2 cells) and the lepidopteran, *Bombyx mori*, (Bm5 cells) to reveal differences in ecdysone receptor (EcR)-dependent luciferase/GFP reporter gene induction caused by 20E and PonA, respectively. Based on the absence or presence of a 2-OH group and changes in the side chain at position 17, differences between the activities of BRs and BR-hybrids could be observed in the two cell lines. The two tested BRs, 24-epi-brassinolide (24BL) and 24-epi-castasterone (24CS), showed no agonistic or antagonistic activity in both cell lines. Concerning tests with BR-hybrids, both (22R)-CS/PonA and (22S)-CS/PonA showed a dose-dependent induction of the EcR-dependent reporter construct in S2 cells. In Bm5 cells, on the other hand, only (22R)-CS/PonA was able to induce the EcR-dependent reporter construct. No induction of the reporter construct was found for the other BR-hybrids indicating that they do not interfere in the ecdysteroid signaling pathway in an agonistic manner. For antagonistic experiments with BR-hybrids, (20R,22R)-20,22-dihydroxycholesterol, 20-epi-6-deoxoteasterone, (20R,22S)-20,22-dihydroxycholesterol were able to reduce the induction of the EcR-dependent reporter construct by PonA in Bm5 cells. However, no reduction in responsiveness of the reporter construct by 20E was found when using BR-hybrids in S2 cells. The results indicated that the two tested BRs show no activity on both S2 and Bm5 cell lines whereas BR-hybrids were found to show differential agonistic/antagonistic effects dependent on the origin of insect cell line (dipteran versus lepidopteran). Because S2 cells seemed less sensitive for BR-hybrid than Bm5 cells, it is suggested that BR-hybrids interfere better in the ecdysteroid signaling pathway of the lepidopteran cell line than in those of dipteran cell line.

# Research article: Microarray profiles of organophosphate pesticides in *Daphnia magna*

**DOI:** 10.1673/031.007.1387

**Published:** 2007-03-08

**Authors:** T Vandenbrouck, A Soetaert, P van Remortel, WM De Coen

**Affiliations:** ^1^ Laboratory for Ecophysiology, Biochemistry and Toxicology, Department of Biology, University of Antwerp, Belgium.; ^2^ Intelligent System Lab, Department of Mathematics and Informatics, University of Antwerp, Belgium

The freshwater flea *Daphnia magna* is frequently used as a standard organism in laboratory toxicity testing in order to evaluate the potential hazards of chemicals to the aquatic environment. Moreover, this organism has been shown to be sensitive to a wide range of toxicants. Using differential gene expression assessments, more specifically, the recent developed custom made *Daphnia magna* array (Soetaert A et al. 2006) consisting of gene fragments related to the molting, energy metabolism and life-stage specific process enables the characterization of different classes of chemicals. As a semi-high throughput technique, the microarray offers a fast way to evaluate thousands of genes and possible endpoints simultaneously. The aim of this study was to investigate the mode of action of different pesticides. Organophosphate pesticides are known to be acutely toxic and specifically designed to inhibit the activity of the neurotransmitter acetyl cholinesterase. Thereby, they cause interference with the cholinergic nerve transmission system, which eventually can lead to convulsion and death of the organisms. In practice, *Daphnia* were exposed for 48 and 96h to chlorpyrifos and diazinon and the gene expression profiles of the individual compounds were determined. Several molting related genes (e.g. cuticle proteins) showed significant up- or down regulation after exposure to the different treatments. Real-time PCR was used to validate these gene expression results. In future experiments, examining effects on the protein expression as well as on the population level will enable us to link different levels of biological organization and select genes or proteins as possible biomarkers. This study was financed by the EU project, NoMIRACLE.

# Research article: Dual control of ecdysteroidogenesis in the migratory locust, *Locusta migratoria*

**DOI:** 10.1673/031.007.1388

**Published:** 2007-03-08

**Authors:** T Vandersmissen, A De Loof, SH Gu

**Affiliations:** ^1^ Laboratory of Developmental Physiology, Genomics & Proteomics, Zoological Institute, K.U.Leuven, Belgium.; ^2^ Department of Zoology, National Museum of Natural Science, Taichung, Taiwan.

Ecdysteroidogenesis by the prothoracic glands is activated by the neuropeptide prothoracicotropic hormone (PTTH). In the silkworm, *Bombyx mori*, not only PTTH but also an as yet unidentified autocrine factor (AF) activate ecdysteroid secretion in vitro. To find out whether or not this dual control exists in other species, in particular in heterometabolous ones, we applied similar methods as were used to discover AF in *Bombyx* to the locust *Locusta migratoria*. The results showed that either decreasing the incubation volume from 50 to 5 µl, or increasing the number of glands incubated per drop (50µl) from 1 to 8 significantly increased ecdysteroid secretion by the glands of day 7 last instar nymphs. Ecdysteroidogenesis was also activated, in a dose-dependent manner, by prothoracic gland-conditioned medium. Co-incubation of the prothoracic glands with brains stimulated ecdysteroid secretion, indicating the existence and action of an as yet unidentified locust PTTH. When PTTH and AF were combined, ecdysteroid secretion was higher as compared to stimulation by either PTTH or AF alone, indicating that PTTH and AF act through different receptors. During the last nymphal instar, differences in responsiveness to PTTH and AF were observed. The functions of AF *in vivo*, as well as its chemical identity remain to be elucidated.

# Research article: Endocrine disruption in crustaceans: chasing the insect field

**DOI:** 10.1673/031.007.1389

**Published:** 2007-03-08

**Authors:** T Verslycke

**Affiliations:** Woods Hole Oceanographic Institution, Biology Department MS#32, Woods Hole, MA 02543, USA.

Existing screening and testing programs for endocrine disrupters (EDs) focus on vertebrates and a small number of nuclear receptors (i.e. estrogen, androgen, and thyroid). The physiological and developmental roles of most invertebrate receptors and their ligands have diversified so thoroughly that endpoints used as indicators of EDs based on function in mammals will likely be inappropriate for invertebrates. At least nine ecdysozoan genomes have been partly or completely sequenced and the number of invertebrate genome projects is rapidly increasing. Insects, crustaceans, spiders, nematodes play major roles in terrestrial and aquatic food webs, and some species are of considerable economic and cultural importance. Application of numerous ecdysozoan species in regulatory toxicity testing has been standardized. Specifically, crustaceans have been used extensively in standard toxicity testing and are also being considered as invertebrate models for ED testing by different regulatory instances. Yet, basic knowledge of crustacean hormones and their functions is limited compared to what is known for some insects. The issue of environmental endocrine disruption, and specifically the issue of non-target effects of insect growth regulators (IGR) in crustaceans, has revived the field of crustacean endocrinology. Rapid progress in the -omics field provides exciting opportunities that could assist our understanding of hormonal regulation and its potential disruption in crustaceans. This talk will give an overview of recent advances in crustacean ED research with a focus on IGR effects. Recent projects that focus on lobster shell disease, marine copepod diapause, and mysid hormone receptor identification, highlight the need for stronger interactions between insect endocrinologists and toxicologists that have pursued the issue of endocrine disruption in invertebrates by using established crustacean models.

# Research article: Phytoecdysteroids: fundamental and applied studies

**DOI:** 10.1673/031.007.1390

**Published:** 2007-03-08

**Authors:** VV Volodin, IF Chadin, S Volodina, L Dinan

**Affiliations:** ^1^ Institute of Biology, Russian Academy of Sciences, 28 Kommunisticheskaya Str., Syktyvkar, 167982, Russian Federation.; ^2^ Insect Biochemistry Group, Hatherly Laboratories, Biological Sciences, University of Exeter, Exeter, Devon UK

Our strategy for the screening of plants in regional floras for ecdysteroid presence based on chemotaxonomic principles has been extended. Correlations between the distribution of ecdysteroids and the phylogenetic classification of plant species are identified for species with high ecdysteroid content (positive in the BII bioassay). Trace amounts of ecdysteroids (positive in ecdysteroid-specific RIA, but negative in the Bu bioassay) are found in young tissues of most vascular plants independently of their systematic position. Wide screening of the flora of European North East of Russia and selected screening of floras of Southern Ural, Northern Caucasus, Russian Far East and China has been carried out. It is established that ecdysteroids are characteristic to the plants of southern latitudes and polyzonal groups. The contribution of ecological and geographical factors on the content and composition of major and minor ecdysteroids in plants of the genus *Serratula* (Asteraceae; *S. coronata*, *S. inermis*, *S. gmelinii*, *S. radiata*, *S. quinquefolia*) has been investigated. It is shown that the distribution of ecdysteroids within the plants is determined by the bio-morphological peculiarities of plants. The contribution of ecdysteroids as a deterrent strategy in *Serratula coronata* is revealed. Particular features of ecdysteroid biosynthesis in cell cultures obtained from intact plants with high (*Serratula coronata*), moderate (*Rhaponticum carthamoides*) and low (*Ajuga reptans*) ecdysteroid levels have been studied. It is shown that introduction of methyl jasmonate into nutrient media leads to an increase in total ecdysteroid content in cell cultures. The composition of the new ecdysteroid-containing nutritional supplement *“Serpisten”*, which is based on the leaves of the plant *Serratula coronata*, and the technology of its production will be presented. This preparation has strong anti-ischaemic and anti-diabetic effects. The data obtained testhy to the perspectives for further pharmacological study of phytoecdysteroids as adaptogenic and metabolic remedies. We thank the Interregional Centre “Adaptogen” in Saint-Petersburg for the pharmacological study of the preparation *Serpisten.* This research was supported by grants from the INTAS (96–1291), the Programme of Collaboration between Ural and Far East Divisions of Russian Academy of Sciences (Project N 48a) and the Programme of the Presidium of RAS “Fundamental sciences for medicine”

# Research article: FXPRL-amide peptides induce ecdysteroidogenesis through the activation of their receptor expressed in the prothoracic gland *of Bombyx mori*

**DOI:** 10.1673/031.007.1391

**Published:** 2007-03-08

**Authors:** K Watanabe, T Yaginuma, S Matsumoto, K Imai, H Kataoka

**Affiliations:** ^1^ Department of Integrated Biosciences, Graduate School of Frontier Sciences, The University of Tokyo, Chiba 277–8562, Japan.; ^2^ Graduate School of Bioagricultural Sciences, Nagoya University, Nagoya 464–8601, Japan; ^3^ The Institute of Physical and Chemical Research (RIKEN), Saitama 351–0198, Japan; ^4^ Faculty of Bioresources, Mie University, Tsu 514–0008, Japan

FXPRL-amide peptide family (pyrokinin/PBAN family) consists of the insect peptides that have the conserved motif in C-terminal structure, and these peptides broadly function in insect life processes. In the silkworm, *Bombyx mori*, five FXPRL-amide peptides are produced by a single gene (*DH*-*PBAN* gene). It is known that *Bombyx* FXPRL-amide peptides regulate activation of pheromone biosynthesis and induction of embryonic diapause in females. However, *DH-PBAN* gene is also expressed in the SOG of male larvae and pupae, which suggests that these peptides have unknown functions independent of sex during post-embryonic development. Recently, two FXPRL-amide peptides receptors (BmPBANR and BmDHR) were identified in *B. mori* (Hull J et al. 2003. J. Biol. Chem. 279: 51500–51507; Homma T et al. 2007. Biochem. Biophys. Res. Commun, in press.), which would help revealing as-yet-unknown functions of FXPRL-amide peptides. In this study, we performed reverse transcriptase-polymerase chain reaction analyses of these receptors with various tissues from wandering fifth instar larvae and found the expression of *BmDHR* in prothoracic gland (PG). Activation of BmDHR increases intracellular Ca^2+^concentration and cAMP content and induces ecdysteroidogenesis in PG at the late stage of fifth instar. These results suggest that FXPRL-amide peptides regulate ecdysteroidogenesis *in vivo* and have an essential role in controlling molting and metamorphosis.

# Research article: Identification of FMRFamide-related peptides as novel prothoracicostatic factors

**DOI:** 10.1673/031.007.1392

**Published:** 2007-03-08

**Authors:** N Yamanaka, D Zitnan, A Mizoguchi, Y Tanaka, H Kataoka

**Affiliations:** ^1^ Department of Integrated Biosciences, Graduate School of Frontier Sciences, The University of Tokyo, Chiba 277–8562, Japan.; ^2^ Institute of Zoology, Slovak Academy of Sciences, Dúbravská cesta 9, 84206 Bratislava, Slovakia; ^3^ Division of Biological Science, Graduate School of Science, Nagoya University, Nagoya 464–8602, Japan.; ^4^ National Institute of Agrobiological Science, Ibaraki 305–8634, Japan

FMRFamide and its related peptides are among the most extensively studied neuropeptides in animals. The tetramer FMRFamide was originally isolated from the clam *Macrocallista nimbosa* as a cardioexcitatory neuropeptide. Using antisera raised against the peptide, it was shown later that the FMRFamide-related peptides (FaRPs) exist throughout invertebrates including insects, usually with extended N-termini. Here we present the identification of FaRPs as prothoracicostatic factors in the silkworm *Bombyx mori*. We first identified Bommo-myosuppressin (BMS), a member of the FaRPs, as a novel prothoracicostatic factor (Yamanaka N et al. 2005. J. Biol. Chem. 280: 14684–14690). The BMS receptor (BMSR) is highly expressed in the prothoracic gland (PG), consistent with the inhibitory action of BMS on the gland. Interestingly, BMSR responded to two other FaRPs from another lepidopteran species (*Manduca sexta*), which also exert inhibitory effects on *Bombyx* PGs. These results suggested that some other endogenous FaRPs in *Bombyx* may also act as prothoracicostatic factors. Based on the above results, we next purified and identified four *Bombyx* extended FMRFamides (Bommo-FMRFamides, BRFa) as novel prothoracicostatic factors. These neuropeptides are encoded by the same gene. We further showed that BRFa are produced in the CNS neurons which suppress the PG activity by direct innervation. Although the importance of the PG innervating neurons in the control of ecdysteroidogenesis has been well-documented, studies revealing molecular basis of the PG regulation have been restricted to hormonal substances throughout the last century. This is, to our knowledge, the first report of peptides controlling ecdysteroidogenesis by direct innervation, which may shed light on the novel aspect of the regulatory mechanism of insect development (Yamanaka, N. et al., *Proc. Natl. Acad. Sd. USA*, in press). Recent results obtained during the search for other regulators of the PG activity will also be presented.

# Research article: Transcriptional regulation of DIAP1 levels provides competence for ecdysone-triggered salivary gland cell death

**DOI:** 10.1673/031.007.1393

**Published:** 2007-03-08

**Authors:** VP Yin, A Bashirullah, CS Thummel

**Affiliations:** Department of Human Genetics, Howard Hughes Medical Institute, University of Utah School of Medicine, 15 North 2030 East Room 5100, Salt Lake City, UT 84112–5331, USA.

Sequential pulses of ecdysone direct the massive destruction of obsolete larval tissues during *Drosophila* metamorphosis, providing a model system for defining the molecular mechanisms of steroid-regulated programmed cell death. In *Drosophila*, death activators such as *reaper* and *hid* trigger programmed cell death by relieving Inhibitor of Apoptosis Protein (IAP)-mediated inactivation of ubiquitous caspases. Here we show that the levels of *Drosophila* IAP 1 (DIAP1) are critical for the proper temporal patterns of ecdysone-triggered cell death. During larval stages, high levels of DIAP1 block caspase activation in salivary glands, even in the presence of ectopic death activator overexpression. The transcriptional down-regulation of *diap1* prior to metamorphosis is necessary to sensitize salivary glands for their subsequent destruction in response to ecdysone-induced *reaper* and *hid* expression. This switch in *diap1* expression is mediated by CBP in an ecdysone-dependent manner during the mid-third instar transition. Steroid-triggered cell death is thus a two-step temporally-regulated response, with the first step providing competence for death and the later step driving tissue execution.

# Research article: Functional analysis of two evolutionally conserved genes, *neverland* and *CRABP*, which are predominantly expressed in the prothoracic gland

**DOI:** 10.1673/031.007.1394

**Published:** 2007-03-08

**Authors:** T Yoshiyama, T Namiki, K Mita, H Kataoka, R NiWa

**Affiliations:** ^1^ Department of Integrated Biosciences, Graduate School of Frontier Sciences, The University of Tokyo, Chiba, Japan.; ^2^ Laboratory of Insect Genome, National Institute of Agrobiological Sciences, Tsukuba, Ibaraki, Japan.

During larval and pupal development of insects, ecdysone is synthesized in the prothoracic gland (PG). However, many aspects of the ecdysteroid synthesis pathway in the PG have still remained unclear at molecular level. Here, we report the identification and characterization of genes highly expressed in the PG through a combined approach using molecular and genetic analysis. One of the genes we have identified is *neverland* (*nvd*), an evolutionally conserved Rieske-like oxygenase. *nvd* genes of both the silkworm *Bombyx mori* and the fruit fly *Drosophila melanogaster* were expressed specifically in tissues that synthesize ecdysone, such as the PG. We have shown that loss of *nvd* function in the PG causes arrest of both molting and growth during *Drosophila* development. Furthermore, the phenotype was rescued by application of 20-hydroxyecdysone or the precursor 7-dehydrocholesterol, suggesting that Nvd is a novel and essential regulator for cholesterol metabolism or trafficking in steroid synthesis. We have also identified the other gene predominantly expressed in the PG, *Bombyx* ortholog of *cellular retinoic acid binding protein* (*BmCRABP*). Mammalian CRABPs are known to bind to small chemicals such as retinoic acid. We will present data on the biochemical characterization of BmCRABP and the role of a family of insect CRABPs in ecdysone biosynthesis in the PG.

# Research article: Microarray analysis of the molting process in the spruce budworm, *Choristoneura fumiferana*.

**DOI:** 10.1673/031.007.1395

**Published:** 2007-03-08

**Authors:** D Zhang, T Ladd, S Zheng, L Li, D Buhlers, P Krell, BM Arif, Q Feng, D Doucet

**Affiliations:** ^1^ Great Lakes Forestry Centre, Canadian Forest Service, Natural Resources Canada, 1219 Queen Street East, Sault Ste. Marie, Ontario, Canada, P6A 2E5.; ^2^ Department of Microbiology, University of Guelph, Guelph, Ontario, Canada, N1G 2W1

Molting is a complex process of development that involves shedding the old cuticle and producing a new exoskeleton for the life stage. Insect molting process is controlled by a cascade of gene expression that is initiated by the insect molting hormone 20-hydroxyecdysone (20E). To understand the larval molting events in the spruce budworm *Choristoneura fumiferana* at the genome level, a cDNA-based microarray containing 3,000 PCR elements represented by ∼15,000 ESTs from *C. fumiferana*, which is one of the most widely distributed and destructive defoliators in Canada, has been constructed and used to analyze gene expression profiles in larvae molting from 5^th^ to 6^th^instar stages. Genes represented by 332 unigenes showed at least three-fold difference in the expression level between molting and intermolting larvae. These genes are involved in several biological processes such as cuticle synthesis and degradation, chitin synthesis and degradation, cuticle pigmentation, myogenesis, transcription and translation regulation, sensory system development, metabolism pathways in digestion, cell proliferation and death etc. According to the expression patterns, these genes can be clustered into four major groups in a clustergram. The genes in these different groups response to 20E differently. Microarray data of the expression profiles was confirmed using quantitative reverse transcription PCR. The relationships between molting and gene expression patterns as well as some novel genes that have potential to be used in pest control are discussed.

# Research article: 20-Hydroxyecdysone accumulation in *Silene* species (*Caryophyllaceae*) without apex

**DOI:** 10.1673/031.007.1396

**Published:** 2007-03-08

**Authors:** L Zibareva, N Ivanova, V Yeryomina, Yu Doropheeva

**Affiliations:** Tomsk State University, Siberian Botanical Garden, Russia.

*Silene frivaldszkyana* and *Silene linicola* are very perspective sources of various phytoecdysteroids (Zibareva 2000. Archives of Insect Biochemistry and Physiology. 43. P. 1–8). These species grow in the West Europe and are studied in culture in conditions of West Siberia for many years. Synthesis high levels various ecdysteroids is characteristic for these plants (Mamadalieva NZ et. al., 2002. Khimiya Prirodnykh Soedinenii 3: 225–227). Overground part *S*.*frivaldszkyana* contains 3–4 % 20-hydroxyecdysone in the beginning of vegetation and 7–8 % in reproductive organs, *Silene linicola -* 0.9 % in flowering and 1.6 % in flowers. The greatest concentrations of ecdysteroids as in annual and perennial species are accumulated in young developing organs. The gradient of concentrations is observed in all organs in development. Removal apical part for plants *Silene linicola* and *S*.*frivaldszkyana* results in delay of growth to high at 1.5–1.8 time and to formation a lateral shoots. In plants without apex there is a redistribution of the contents 20-hydroxyecdysone, it accumulate in growing lateral shoots. Synthesis ecdysteroids in these samples is accelerated. The quantityof 20-hydroxyecdysone increases 1.7 times at overground part of *Silene linicola*. Thus, removal apex influences both biology of development of plants, and on ecdysteroids distribution, quantity, that can testify to the important physiological role phytoecdysteroids in a plant organism.

# Research article: Comparison of phytoecdysteroids in various sections of the genus *Silene* (Caryophyllaceae)

**DOI:** 10.1673/031.007.1397

**Published:** 2007-03-08

**Authors:** L Zibareva, R Lafont, L Dinan, D Puk

**Affiliations:** ^1^ Tomsk State University, Siberian Botanical Garden, Russia.; ^2^ Université Pierre & Marie Curie, 7 Quai St. Bernard, F-75252 Paris 05, France; ^3^ Insect Biochemistry Group, Hatherly Laboratories, Biological Sciences, University of Exeter, Exeter, Devon UK

*Silene* is one of the richest ecdysteroid-containing genera so far detected in the plant world. Ecdysteroids have been recognised in more than 120 species of the genus. Commonly, the ecdysteroid-positive species possess a rich ecdysteroid composition: high levels of 20-hydroxyecdysone, accompanied by 2-deoxy-20-hydroxyecdysone, 2-deoxyecdysone, polypodine B and integristerone A, are characteristic for the majority of *Silene* species. However, many novel phytoecdysteroids could be also isolated from members of this genus and many analogues have, so far, only been found in plants of this genus. There are certain differences in ecdysteroid variety and levels between species, which indicates that ecdysteroid profiles could be used for chemotaxonomic purposes. 20-Hydroxyecdysone and polypodine B are characteristic for plants of the section Siphonomorpha. Ecdysone, integristerone A and 2-deoxyintegristerone A are characteristic for the section Silene. However, for the section Otites, 20-hydroxyecdysone, ecdysone, 2-deoxy-20-hydroxyecdysone, 2-deoxyecdysone, 2-deoxyintegristerone A, 2-deoxy-21-hydroxyecdysone and sidisterone are typical. From *S. gigantea* (sect. Siphonomorpha), we isolated the characteristic ecdysteroids for this section. However, in *S. frivaldszkyana* (sect. Sclerocalycinae [Zibareva L. 2000. Archives of Insect Biochemistry and Physiology 43: 1–8]), in addition to the expected ecdysteroids, the 26-hydroxy derivatives of 20-hydroxyecdysone and polypodine B were detected. The composition of the ecdysteroid mixtures is characteristic for the sections of the genus *Silene*. Data regarding the detailed ecdysteroid profiles and contents of *Silene frivaldskyana* and *Silene gigantea* will be presented. The ecdysteroid pattern of the former species is similar to that of *Silene nutans* and, as such, it represents an excellent source for the rare ecdysteroid 20,26-dihydroxyecdysone.
